# Comparative Analysis of the Donor Properties of Isomeric Pyrrolyl Phosphine Ligands

**DOI:** 10.1021/acs.organomet.3c00467

**Published:** 2023-12-22

**Authors:** Vicky A. Osenga, Nolan C. Sykes, Sopheak Pa, Michael K. Bambha, Nathan D. Schley, Miles W. Johnson

**Affiliations:** † Department of Chemistry, University of Richmond, Richmond, Virginia 23173, United States; ‡ Department of Chemistry, Vanderbilt University, Nashville, Tennessee 37235, United States

## Abstract

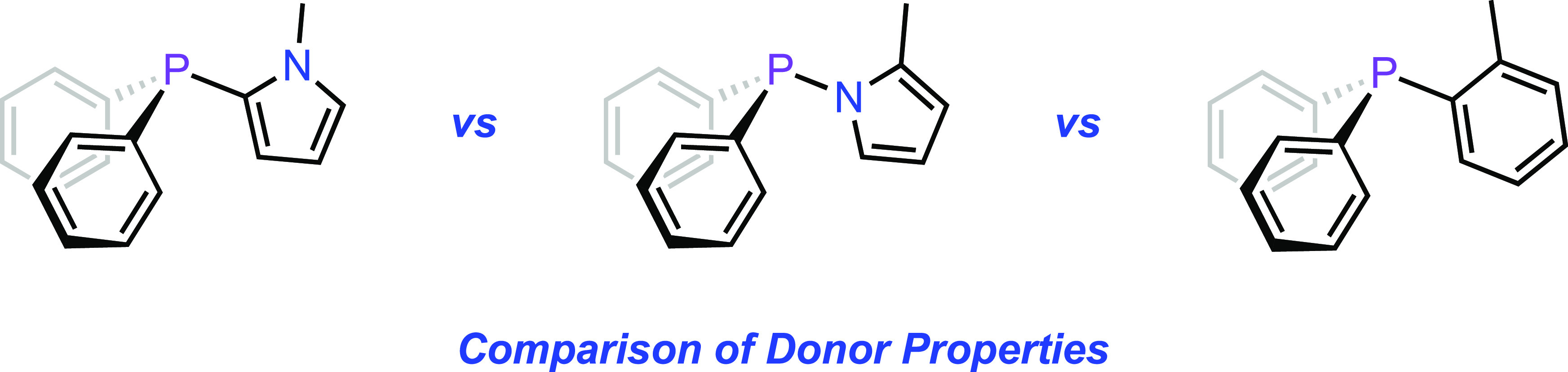

Understanding the net donor and electronic properties of pyrrole-based phosphines is critical for guiding their use as ligands. In this study, we compare two isomeric 1- and 2-(diphenylphosphino)methylpyrroles (**L1** and **L2**, respectively) to determine the degree to which *N*-(phosphino)pyrroles are distinct from aryl- and 2-pyrrolyl phosphines. Ruthenium, rhodium, platinum, and gold complexes as well as selenide derivatives of these ligands are examined using NMR and IR spectroscopy, X-ray crystallography, and cyclic voltammetry. Ligand **L2** exhibits net donor properties similar to those of the *o*-tolyl analogue **L3**, while **L1** shows attenuated electron donation ability. Additionally, a model nickel-catalyzed Kumada coupling reaction using these three ligands was investigated.

## Introduction

**Figure 1:**
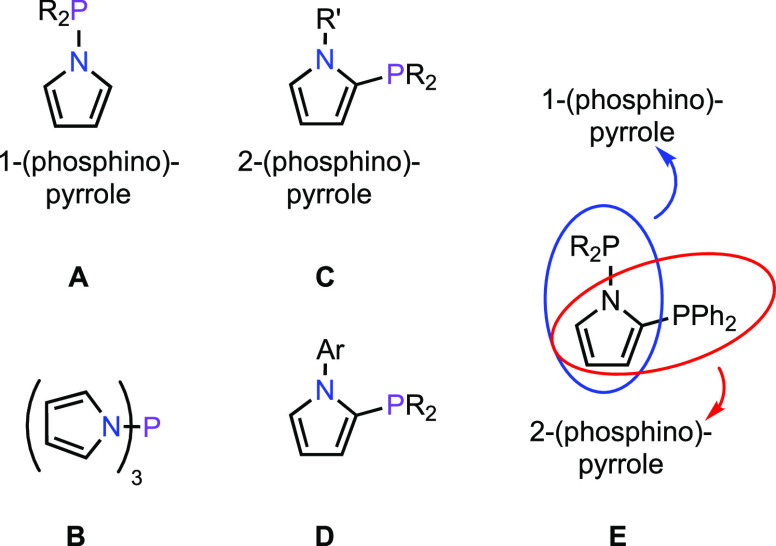
Representative examples of phosphinopyrrole ligands.

The pyrrole moiety offers a versatile foundation from which to construct a variety of ligands for transition metals. Phosphinopyrroles (Figure 1) are a notable subclass of pyrrole-derived ligands because of their myriad applications in coordination chemistry and catalysis. *N*-pyrrolyl phosphine ligands (Figure 1, **A**), which are strongly π-accepting and were first studied extensively by Moloy and Petersen,^1^ are used to promote hydroformylation,^2,3^ oligomerization,^4^ fast-initiating olefin metathesis,^5^ and enantioselective^6^ reactions, and have been analyzed in depth.^7^ Tris(*N*-pyrrolyl)phosphine^8^ (**B**) has been particularly valuable in these and related studies. 1-(Phosphino)pyrroles are also components of novel caged phosphine^9^ and pincer ligands.^10^ 2-(Phosphino)pyrroles (**C**), the first example of which was prepared and studied by Allen and co-workers,^11^ offer yet more ligand architectures and new avenues for reactivity. This class of ligand has been employed in carbonylation,^12^ C–H functionalization,^13^ hydrogenation,^14^ and ethanol upgrading reactions.^15^ Perhaps the most notable 2-(phosphino)pyrroles are the highly modular *N*-aryl 2-(phosphino)pyrrole cataCXium ligands (**D**), which enable a variety of efficient cross-coupling reactions.^16,17^ 2-(Phosphino)pyrroles have also been used as bridging ligands in multimetallic complexes^18−20^ and as components of pincer ligands.^21,22^ 3-(Phosphino)pyrrole-based ligands have received far less attention, but their exceptionally strong donor ability has been recently investigated.^23^

Despite the importance of phosphinopyrroles in coordination chemistry and catalysis, to the best of our knowledge, a comprehensive and comparative analysis of the donor properties of 1- and 2-(phosphino)pyrroles and their impact on their metal complexes has yet to be undertaken. Comparisons can be made between these two substitution patterns by looking closely at over 40 years of the literature; however, there is little opportunity for direct comparison between an *N*- and 2-(phosphino)pyrrole in which convoluting variables are controlled. A comparison of isomeric phosphinopyrroles is needed to definitively distinguish the properties distinctive to each of these molecules as ligands. Quantitative data concerning ligand properties can inform researchers in the rational design, parametrization, and selection of ligands for catalytic reactions.^24−26^ This study is motivated in part by the need to decouple the donor properties of the two phosphorus centers present in the 1,2-bis(phosphino)pyrroles developed in our own laboratory (Figure 1, **E**).^27^ Herein, we elucidate the net donor properties of model 1- and 2-(phosphino)pyrrolyl phosphine ligands and their impact on a series of transition-metal complexes.

## Results and Discussion

**Scheme 1:**
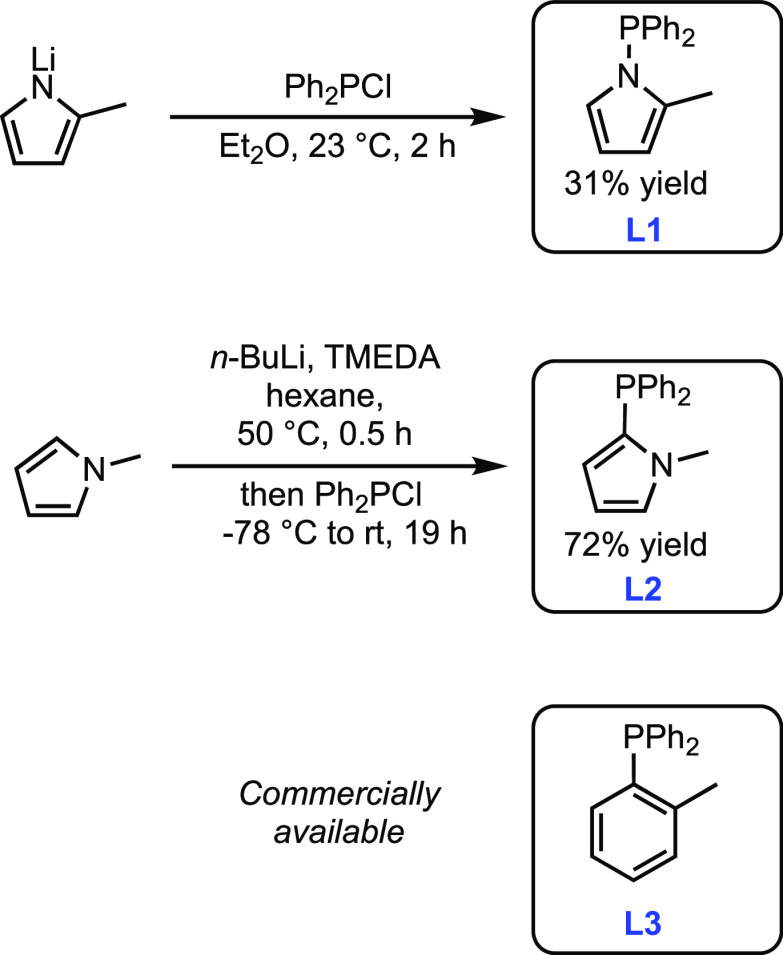
Synthesis of Model Phosphines

**Figure 2:**
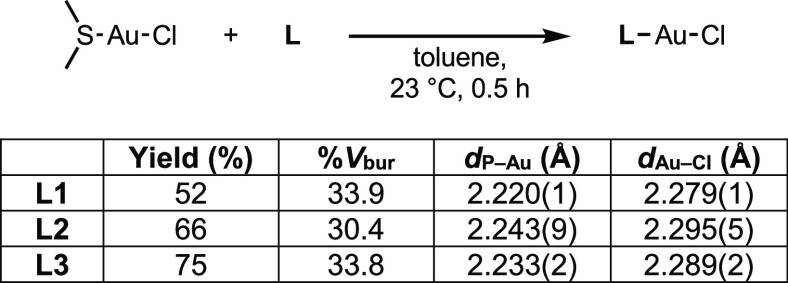
Synthesis of **L**AuCl complexes and key metrics.

**Figure 3:**
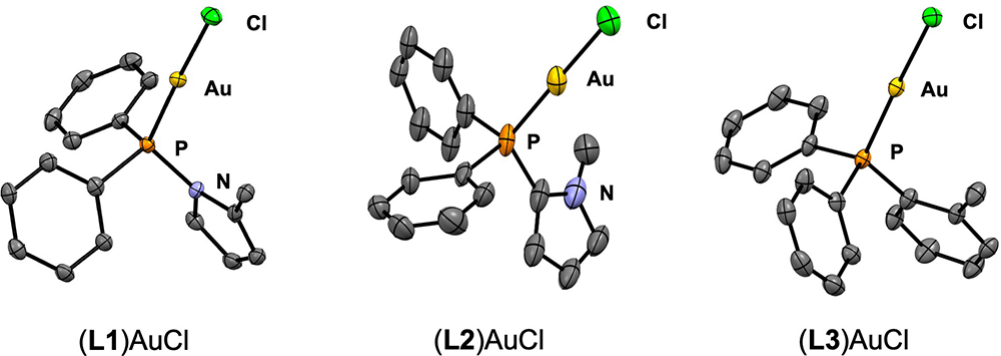
Solid-state structures of **L**AuCl. Thermal ellipsoids are shown at 50% probability. Disorder, solvents, and all hydrogen atoms have been omitted for clarity.

1-(Diphenylphosphino)-2-methyl-pyrrole (**L1**), 2-(diphenylphosphino)-1-methyl-pyrrole (**L2**), and diphenyl(*o*-tolyl)phosphine (**L3**) were selected as model systems for this study (Scheme 1). These compounds were chosen because **L1** and **L2** are isosteric isomers that cannot engage in hydrogen bonding or be easily deprotonated to form bidentate or bridging ligands.^28^ The similarity in steric profiles of **L1**, **L2**, and **L3** is demonstrated by the cone angles of triphenylphosphine and P(NC_4_H_4_)_3_^29^ as well as the similar minimum percent buried volumes (% *V*_bur_) of CyJohnPhos and cataCXium PCy.^30^**L1** was synthesized by treatment of lithium 2-methyl pyrrolide with Ph_2_PCl in diethyl ether, and ligand **L2** was prepared by a modification of a literature procedure.^11^ The low yield of **L1** can be attributed to challenges in purification (see the Supporting Information). Each ligand was metalated with DMSAuCl (DMS = dimethylsulfide) to yield the corresponding two-coordinate gold(I) complex (Figure 2), and each complex was characterized by X-ray crystallography (Figure 3). The % *V*_bur_ of each complex was then calculated based on crystallographic data using SambVca 2.1.^31^ The buried volumes of **L1** and **L3** were found to be similar (33.9 and 33.8%, respectively) when examined with a standard sphere radius of 3.5 Å. The value for **L2** was lower (30.4%), potentially because the methyl group is angled outside of the 3.5 Å radius (see the Supporting Information).

**Figure 4:**
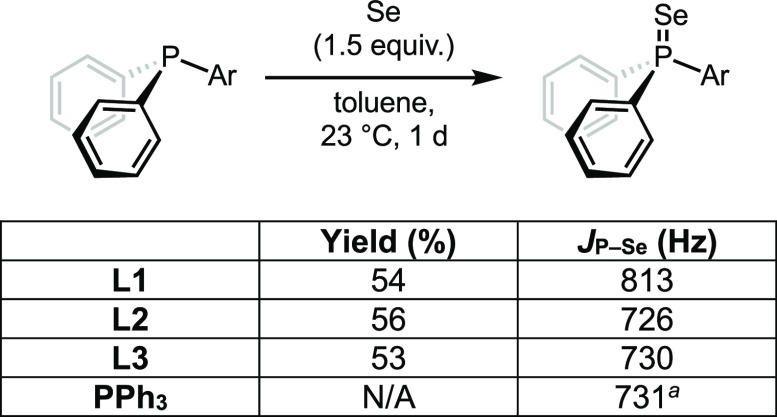
Synthesis of phosphine selenides and the *J*_P–Se_ coupling constants. ^a^Literature value.^26^

The electronic properties of the model phosphines were first examined by synthesizing their corresponding phosphine selenides. These compounds were prepared by treating the appropriate phosphine with selenium (Figure 4). **L2**^**Se**^ (*J*_P–Se_ = 726 Hz) and **L3**^**Se**^ (*J*_P–Se_ = 730 Hz) have similar coupling constants, and the values were in agreement with those of the literature (728 and 730 Hz, respectively)^32^ and markedly lower than that of **L1**^**Se**^ (*J*_P–Se_ = 813 Hz). The P–Se coupling constants for **L2**^**Se**^ and **L3**^**Se**^ are also similar to that of triphenylphosphine selenide (*J*_P–Se_ = 731 Hz). It can be concluded from these data that **L1** has greater s-character in the P–Se bond, which in turn can be attributed to the more electronegative nitrogen bound to phosphorus,^32^ consistent with Bent’s rule.^33^ Importantly, this comparison is not complicated by differences in the NMR solvent,^34^ intramolecular hydrogen bonding,^35^ or difference in steric bulk,^36^ the last of which may perturb the extent of pyramidalization at phosphorus and thus s-character. These data suggest that **L1** is a weaker base and electron donor than the other two ligands^35^ and are in agreement with computed methyl cation affinities for similar phosphines.^37^ In addition to the above implications of these data, it should be noted that *J*_P–Se_ coupling constants have also been used to predict the ligand performance in cross-coupling reactions.^26^

**Figure 5:**
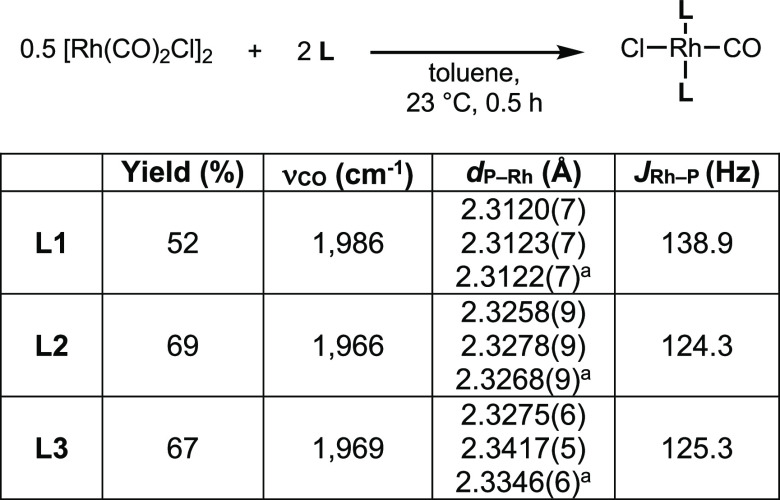
Synthesis of *trans*-(**L**)_2_Rh(CO)Cl complexes, ν_CO_ values, and key metrics. ^a^Average P–Rh bond length.

**Figure 6:**
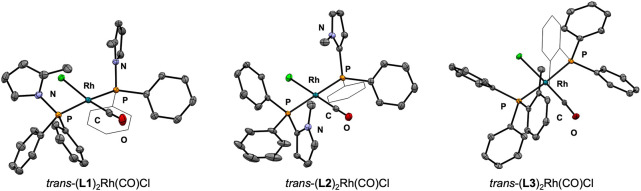
Solid-state structures of *trans*-(**L**)_2_Rh(CO)Cl with thermal ellipsoids are shown at 50% probability. Disorder, solvents, and all hydrogen atoms are omitted for clarity.

**Figure 7:**
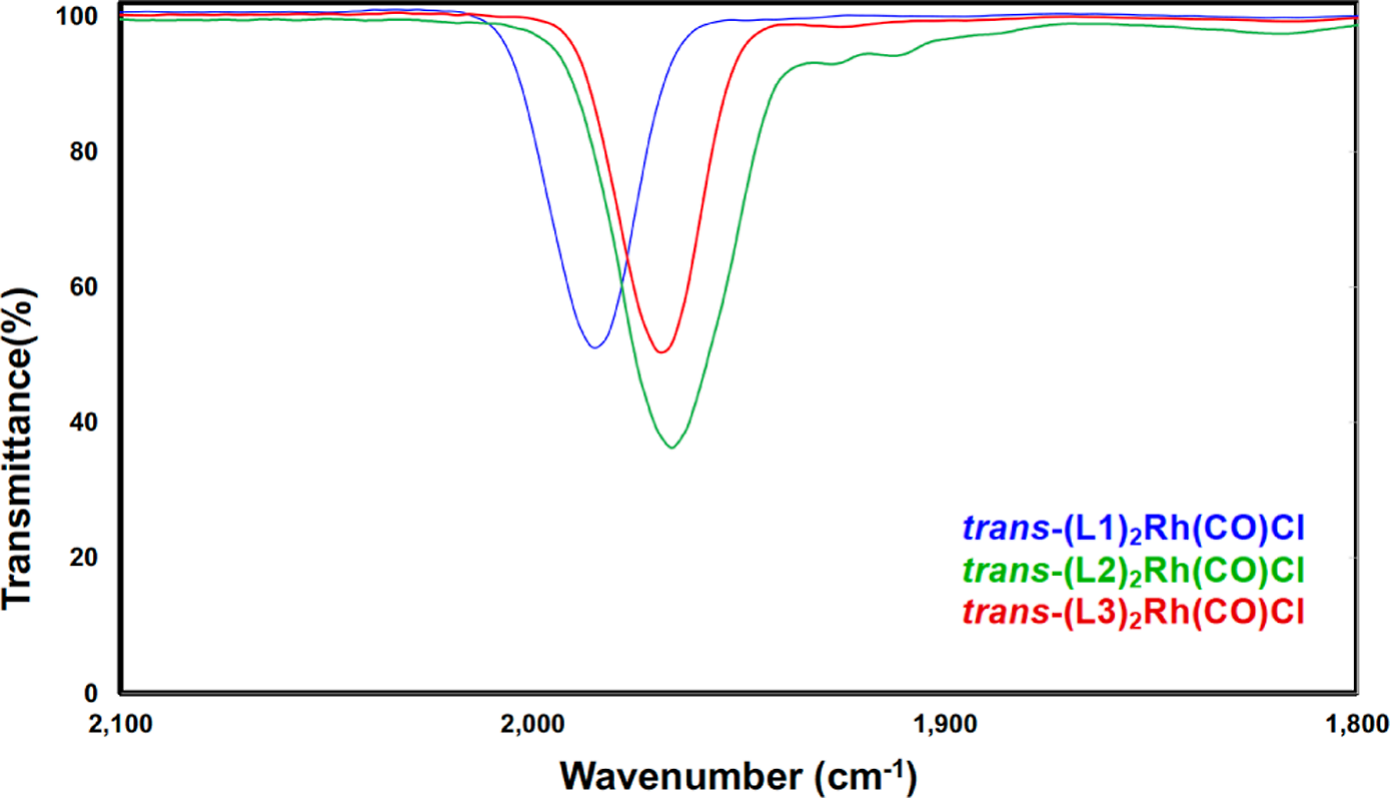
Thin-film ATR–IR spectra of *trans*-(**L**)_2_Rh(CO)Cl.

Vaska-type complexes *trans*-(**L**)_2_Rh(CO)Cl were prepared to determine the donor properties of each phosphine based on the extent of backbonding between rhodium and the carbonyl ligand.^38^ Treatment of [Rh(CO)_2_Cl]_2_ with 2 equiv of phosphine resulted in the formation of the desired complexes (Figure 5), all of which were confirmed by X-ray crystallography (Figure 6). Once again, **L2** and **L3** prove to be similar to one another in donating ability; the CO stretching frequencies for their complexes (1966 and 1969 cm^–1^, respectively) are lower than that for **L1** (1986 cm^–1^), indicating that the rhodium center of *trans*-(**L1**)_2_Rh(CO)Cl is not as electron-rich and therefore less able to engage in backbonding than that in the other two complexes (Figure 7). The shorter Rh–P bonds and higher coupling constants of *trans*-(**L1**)_2_Rh(CO)Cl relative to its two analogues are consistent with previous findings with tris(pyrrolyl)phosphines and pincer ligands with *N*-(phosphino)pyrrole donors.^1,39^ These shorter bonds may be attributed to the abovementioned s-character of the orbital of the phosphorus lone pair of **L1** and/or the ligand’s π-accepting ability^40^ and thus do not necessarily signify stronger bonds, a phenomenon that has been extensively studied.^41−43^

**Figure 8:**
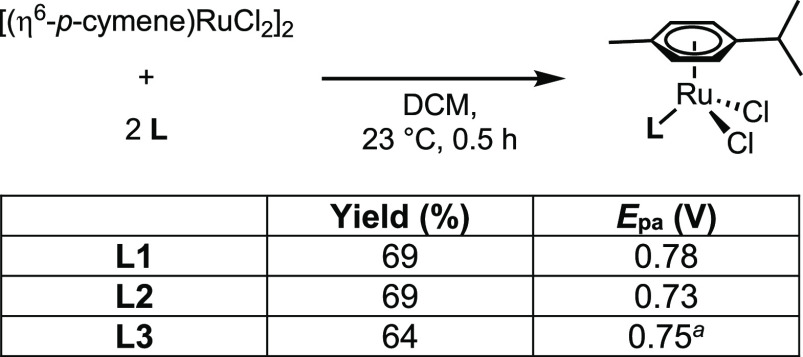
Synthesis of (η^6^-*p*-cymene)Ru(**L**)Cl_2_ complexes and their *E*_pa_. ^a^*E*_1/2_ = 0.70 V.

**Figure 9:**
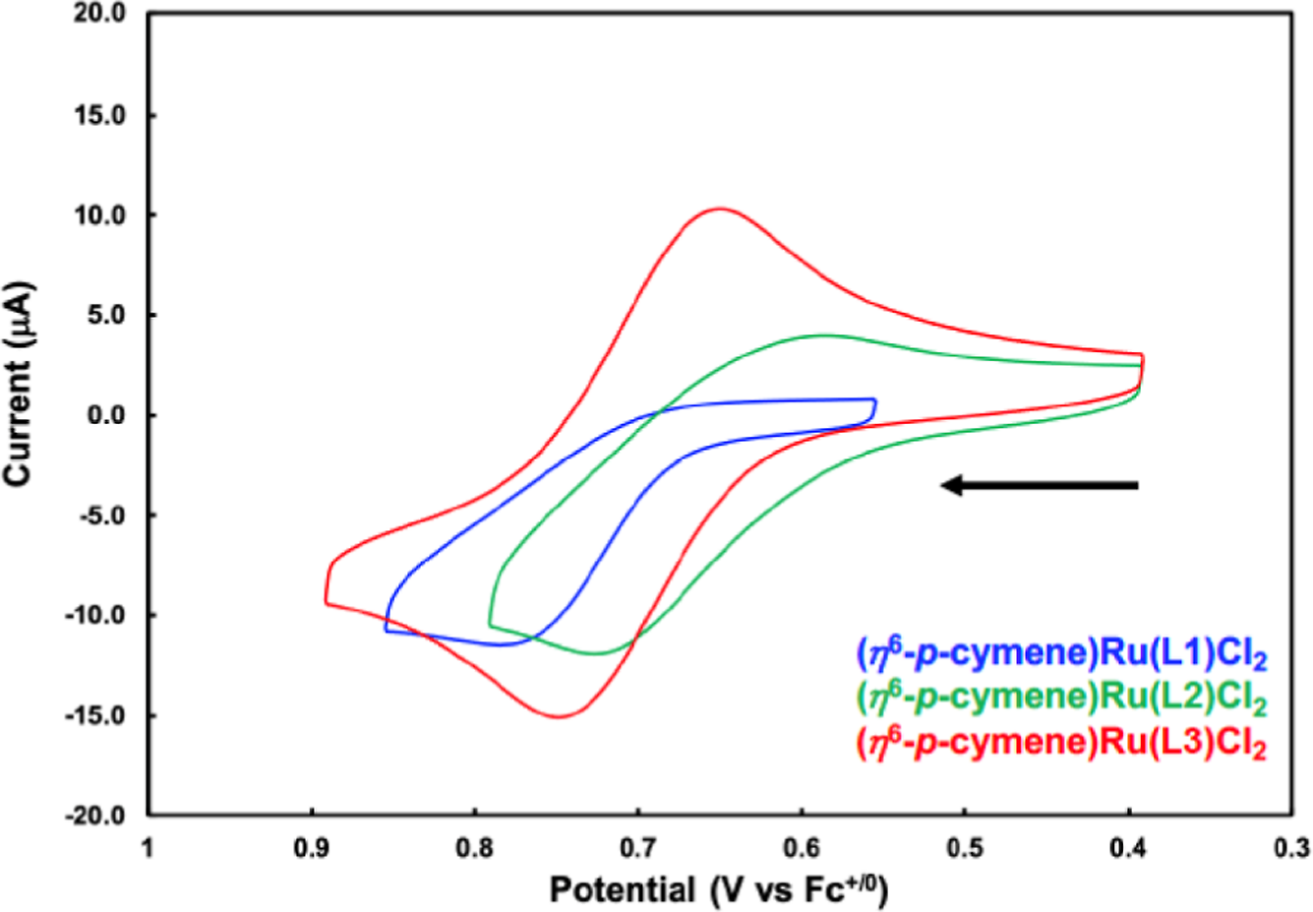
Cyclic voltammograms of (η^6^-*p*-cymene)Ru(**L**)Cl_2_ in CH_2_Cl_2_. Conditions: scan rate: 100 mV/s; supporting electrolyte: 0.1 M [*n*-Bu_4_N][PF_6_]; working electrode: glassy carbon; auxiliary electrode: platinum wire; reference: silver wire referenced to the Fc/Fc^+^ couple.

The impact of phosphine identity on the redox properties of the series of complexes (η^6^-*p*-cymene)Ru(**L**)Cl_2_ was examined by cyclic voltammetry. These complexes were prepared by the treatment of [(η^6^-*p*-cymene)RuCl_2_]_2_ with the corresponding phosphine and purified by crystallization (Figure 8). Oxidation of the three complexes occurs in a 50 mV window with (η^6^-*p*-cymene)Ru(**L1**)Cl_2_ requiring the most positive potential (0.78 V vs Fc/Fc^+^), consistent with **L1** being the least donating of the three ligands, followed by its **L3** (0.75 V) and **L2** (0.73 V) analogues; however, this comparative analysis is complicated by the irreversibility of the oxidation of **L1** and **L2**. It is well documented that increasing the number of additional N-linked pyrroles to phosphorus [e.g., Ph_2_P(NC_4_H_4_) vs P(NC_4_H_4_)_3_] shifts oxidation events to more positive potentials dramatically.^44,45^ Also, it is noteworthy that the reversibility of the redox couples decreases **L3** > **L2** > **L1** (Figure 9), potentially due to the susceptibility of pyrrole toward oxidation and, in the case of **L1**, decreased ligand binding affinity resulting from reduced π-backbonding as the metal center is oxidized.^1,46^ These results suggest that a phosphine bearing a single 1-pyrrolyl substituent imparts a relatively small shift in the oxidation potential of a complex relative to aryl and 2-pyrrolyl substituents but could have implications for additional routes for oxidative decomposition. Indeed, reversibility can be conserved while making subtle changes to redox properties in a related system by employing p-substituted triphenylphosphines.^47^

**Figure 10:**
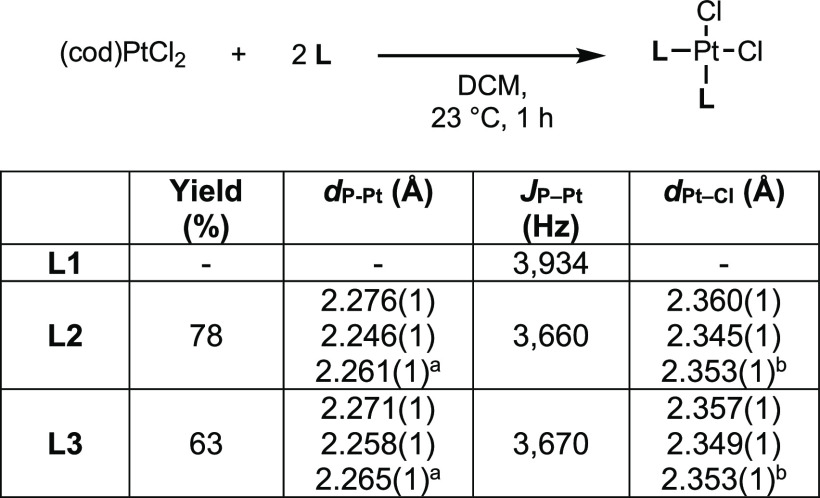
Synthesis of platinum complexes *cis*-(**L2**)_2_PtCl_2_ and their Pt–Cl bond lengths. ^a^Average P–Pt bond length. ^b^Average Pt–Cl bond length.

**Figure 11:**
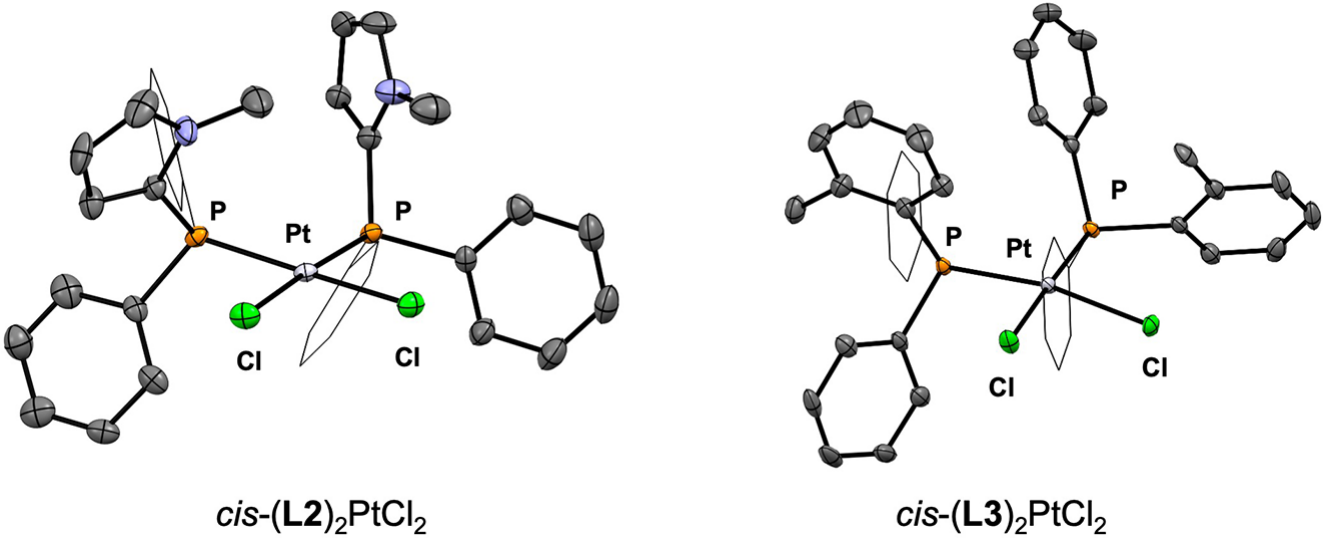
Solid-state structures of *cis*-(**L2**)_2_PtCl_2_ and *cis*-(**L3**)_2_PtCl_2_ with thermal ellipsoids shown at 50% probability. Solvents and all hydrogen atoms are omitted for clarity.

The trans influence^48^ of a ligand plays a critical role in the structure of coordination complexes and on the reactivity of intermediates in catalysis,^49,50^ but, to the best of our knowledge, this property has not been examined with phosphinopyrrole ligands. We first undertook the synthesis of complexes of the type *cis*-**L**_2_PtCl_2_ (Figure 10) to examine the length of the Pt–Cl bonds because the trans influence has been studied extensively in related complexes;^51^ however, synthesis and crystallization of the complete triad proved difficult. *cis*-(**L2**)_2_PtCl_2_ and *cis*-(**L3**)_2_PtCl_2_ were isolated and characterized by X-ray crystallography (Figure 11), but the synthesis of *cis*-(**L1**)_2_PtCl_2_ gave impure mixtures that could not be purified through fractional crystallization. The M–P coupling constants and available bond lengths for this series track with those seen in the *trans*-(**L**)_2_Rh(CO)Cl series, but crystallographic data concerning the Pt–Cl bond for all complexes were not obtained. Fortunately, a trans influence series has been previously examined with linear gold(I) complexes, which has been validated both experimentally^52,53^ and computationally,^54,55^ encouraging us to compare our own series of gold complexes (Figure 2). The Au–Cl bonds of (**L2**)AuCl and (**L3**)AuCl are comparable in length [2.243(9) and 2.233(2) Å, respectively] while that of (**L1**)AuCl (2.220(1) Å) is shorter. These data suggest that **L2** and **L3** impart a similar trans influence that is greater than that of **L1**.

**Figure 12:**
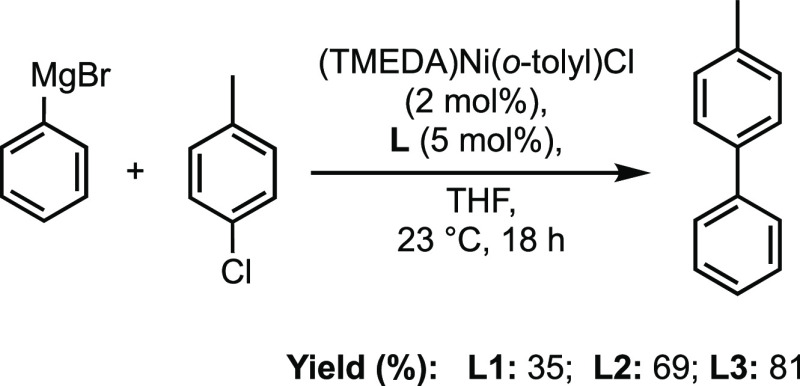
Performance of model ligands in a nickel-catalyzed Kumada cross-coupling reaction. Reported yields are an average of duplicate runs.

The final goal in this investigation was to evaluate potential differences in the catalytic performance for our three model ligands, resulting from their distinct properties. We selected a nickel-catalyzed Kumada cross-coupling reaction,^56^ in which 4-methylbiphenyl is prepared from phenyl magnesium bromide and 4-chlorotoluene (Figure 12). Ligand **L3** showed the highest performance (81% yield) followed by **L2** (69%) and **L1** (35%). Though this model reaction is not extrapolative to all catalytic reactions, these results are consistent with our earlier findings. **L2** and **L3** possess similar steric and electron-donor properties; however, **L3** and catalytic intermediates bearing **L3** may be less susceptible to oxidative degradation. The poor performance of **L1** may stem from its weaker donor ability, but the reactivity of the P–N bond may also be an issue. Indeed, analysis of a catalytic reaction mixture using **L1** shows the formation of PPh_3_, presumably due to the displacement of the pyrrole by the Grignard reagent. Similar reactivity of *N*-(phosphino)pyrroles with strong nucleophiles is documented,^8^ and hydrolysis has been observed with 2-acyl variants.^57^ This observation highlights the need for caution when selecting *N*-(phosphino)pyrrole ligands in processes in which P–N cleavage is possible.

## Conclusions

Experimental examination of a pair of isomeric 1- and 2-(phosphino)pyrrole ligands and their hydrocarbyl congener yielded insights into the impact of these three ligands on the electronic and structural properties of transition-metal complexes. The data are consistent with **L2** being a slightly stronger donor than **L3** and significantly more strongly donating than **L1**. The extent of metal-to-ligand backbonding in rhodium carbonyl complexes and the ease of oxidation of ruthenium cymene complexes are consistent with this conclusion. In all cases, **L2** and **L3** form shorter bonds to metals than **L1** based on solid-state measurements, which are corroborated by M–L coupling constants with spin-active platinum and rhodium nuclei. These phenomena might be attributed to the known π-acidity of *N*-(phosphino)pyrroles and/or the greater s-character in the lone pair orbital of the phosphorus center of **L1**, as determined by the phosphorus–selenium coupling constant of the phosphine selenide. **L2** and **L3** exert stronger trans influences than **L1**, which is also consistent with their comparable donor ability that exceeds that of **L1**. The conclusions drawn from these analyses help rationalize the superior performance of **L2** and **L3** in a model reaction. Furthermore, the lability of the P–N bond of **L1** highlights the need to be cognizant of the reactivity of the ligands themselves as well as the properties that they confer to metals when selecting them for a myriad applications. Though some of the above conclusions may seem intuitive, the preceding study offers an unambiguous comparison that may aid in developing new phosphine ligands and deciding which phosphorus substituents are best suited for a given application.

## Supporting Information Available

Synthesis and characterization, NMR spectra, IR spectra, cyclic voltammograms, catalytic studies, and X-ray crystallographic data (PDF)
