# Supplementary material for: Comparative Analysis of the Donor Properties of Isomeric Pyrrolyl Phosphine Ligands
Source: Organometallics. 2023 Dec 22;43(1):14–20. doi: 10.1021/acs.organomet.3c00467 (PMC10777409; doi:10.1021/acs.organomet.3c00467)
Supplement: Supplementary file 1 — om3c00467_si_001.pdf [file om3c00467_si_001.pdf]

Supporting Information for:

Comparative Analysis of the Donor Properties of Isomeric Pyrrolyl Phosphine Ligands

Vicky A. Osenga,[a] Nolan C. Sykes,[a] Sopheak Pa,[a] Michael K. Bambha,[a] Nathan D. Schley,[b] and Miles W. Johnson*[a]

[a] *Department of Chemistry, University of Richmond, Richmond, Virginia 23173, United States*
[b] *Department of Chemistry, Vanderbilt University, Nashville, Tennessee 37235, United States*

Table of Contents

I. General Information S-2
II. Synthesis and Characterization of Lithium 2-Methylpyrrolide S-4
III. Synthesis and Characterization of Phosphines S-5
IV. Synthesis and Characterization of Phosphine Selenides S-6
V. Synthesis and Characterization of Complexes (η^6-*p*-cymene)Ru(**L**)Cl_2 .... S-8
VI. Synthesis and Characterization of Complexes *trans*-(**L**)$_2$Rh(CO)Cl S-10
VII. Synthesis and Characterization of Complexes *cis*-(**L**)$_2$$PtCl_2$ S-12
VIII. Synthesis and Characterization of Complexes (**L**)AuCl S-14
IX. Ligand Performance in a Model Cross-Coupling Reaction S-16
X. Infrared Spectra of Complexes *trans*-(**L**)$_2$Rh(CO)Cl S-17
XI. Cyclic Voltammograms of (η^6-*p*-cymene)Ru(**L**)Cl_2 .... S-19
XII. Buried Volume Calculation Details for (**L**)AuCl S-21
XIII. Summary of Comparative Data S-23
XIV. NMR Spectra of Lithium 2-Methylpyrrolide S-24
XV. NMR Spectra of Phosphines S-25
XVI. NMR Spectra of Phosphine Selenides S-29
XVII. NMR Spectra of Complexes (η^6-*p*-cymene)Ru(**L**)Cl_2 .... S-35
XVIII. NMR Spectra of Complexes *trans*-(**L**)$_2$Rh(CO)Cl S-41
XIX. NMR Spectra of Complexes *cis*-(**L**)$_2$$PtCl_2$ S-47
XX. NMR Spectra of Complexes (**L**)AuCl S-53
XXI. X-Ray Crystallographic Data S-59
XXII. References S-68

I. General Information

General Considerations. All reactions were carried out in a nitrogen-filled MBraun LABstar Pro glovebox unless otherwise stated. All glassware was oven-dried overnight at greater than 110 °C and cooled under vacuum prior to use. Silica was dried at 150 °C and *ca.* 40 mtorr for 5 h. Anhydrous hexane and pentane were purchased from Sigma and stored over 3 Å molecular sieves prior to use. All other reaction solvents were collected from a Glass Contour Solvent Purification System, degassed, and stored over 3 Å molecular sieves in a glovebox. Dichloromethane-d_2 (CD_2Cl_2), benzene-d_6 (C_6D_6), tetrahydrofuran-d_8 (THF-d_8), and chloroform-*d* ($CDCl_3$) were purchased from Cambridge Isotope Laboratories and stored over 3 Å molecular sieves in a glovebox following degassing. Filtrations were performed using PTFE syringe filters with 0.45 μm pores or glass fiber filters. 2-Methylpyrrole (Ambeed), *N*,*N*,*N'*,*N'*-tetramethylethylenediamine (TMEDA, VWR), and *N*-methylpyrrole (Sigma) were distilled from CaH_2 prior to use. Ph_2PCl (Fisher and Sigma) was distilled under vacuum prior to use. 4-chlorotoluene (VWR) was passed through silica and degassed prior to use. $Pt(cod)Cl_2$ (Strem), $[Rh(CO)_2Cl]_2$ (Strem), [(*p*-cymene)Ru(Cl)(μ-Cl)]$_2$ (Strem), (DMS)AuCl (Strem), diphenyl(*o*-tolyl)phosphine (Ambeed), PhMgCl (Sigma), (TMEDA)Ni(*o*-tolyl)Cl (Strem), and selenium black (powder, Sigma) were used as received. All synthesized metal complexes and selenides can be handled open to air.

NMR Spectroscopy. ^1H, $^{13}C\{^1H\}$, and $^{31}P\{^1H\}$ spectra were collected on Bruker AV-400 and AV-500 NMR spectrometers at ambient temperature. ^1H NMR chemical shifts (δ) are reported in parts per million (ppm) relative to the solvent (5.32 ppm for CD_2Cl_2, 7.26 for $CDCl_3$, 7.16 for C_6D_6, 3.58 and 1.72 for THF-d_8). ^{13}C NMR spectra were referenced relative to the solvent signal (53.84 ppm for CD_2Cl_2, 77.17 for $CDCl_3$, 128.06 for C_6D_6, 67.21 and 25.31 for THF-d_8). $^{31}P\{H\}$ chemical shifts were referenced using the absolute reference function of the Mnova 14.1.0 NMR software package. All spectra were visualized with the same program. Multiplicities are reported as follows: s (singlet), d (doublet), t (triplet), v (virtual), and m (multiplet). Non-centrosymmetric multiplets are reported as ranges.

IR Spectroscopy. Infrared spectra were recorded on a Nicolet iS10 FTIR spectrometer.

X-ray Crystallography. X-ray crystallographic data were collected on a Rigaku Oxford Diffraction Supernova diffractometer. Crystal samples were handled under immersion oil and quickly transferred to a cold nitrogen stream.

Electrochemistry. Electrochemical experiments were performed in a nitrogen-filled glovebox in dichloromethane (DCM) with 0.1 M [*n*-Bu_4N][PF_6] and 1 mM analyte. A CH Instruments 660E potentiostat was used with a 3-mm glassy carbon working electrode, a platinum wire auxiliary electrode, and a silver wire pseudoreference electrode. All potentials were referenced to the ferrocene couple (Fc/Fc^+).[1]

Elemental Analysis. Elemental analyses were performed by Atlantic Microlab (Norcross, GA, USA). Results provided are of samples for which the yield was reported and are the best values obtained to date. The spectroscopic purity of all samples was confirmed by ^1H and ^{31}P NMR spectroscopy before shipment for analysis.

Gas Chromatography and Mass Spectrometry. We wish to thank the UCI Mass Spectrometry Facility for accurate mass measurements. Data were collected on a Waters LCT Premier by flow injection analysis (FIA). Additional high resolution mass spectrometric data were collected using a Shimadzu IT-TOF at the University of Richmond. GC-MS data were acquired using a Shimadzu Nexis GC-2030. The response factor for catalysis studies was determined in triplicate using dodecance and 4-methyltoluene.

II. Synthesis and Characterization of Lithium 2-Methylpyrrolide

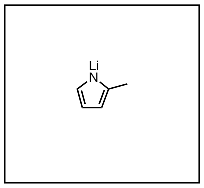

Lithium 2-methylpyrrolide. To a 50-mL round bottom flask was added hexane (15 mL) and 2-methylpyrrole (0.50 mL, 5.8 mmol, 1.0 equiv), and the resulting solution was chilled to -78 °C in a glovebox cold well. *n*-BuLi (3.8 mL of 1.6 M solution in hexane, 6.1 mmol, 1.1 equiv) was then added dropwise via syringe to the chilled solution, resulting in an off-white precipitate. The heterogeneous reaction mixture was stirred at ambient temperature for 1.5 h. The colorless solid was then isolated on a medium porosity frit, and residual solvent was removed under vacuum (383 mg, 4.40 mmol, 76 %). **^{1}H NMR** (500 MHz, THF-d_8): δ 6.47 (br s, 1H, pyrrole), 5.79 (t, J = 2.3 Hz, 1H, pyrrole), 5.61 (m, 1H, pyrrole), 2.22 (s, 3H, CH_3); **^{13}C{^{1}H} NMR** (126 MHz, THF-d_8): δ 134.9, 125.4, 106.7, 104.7, 17.7.

III. Synthesis and Characterization of Phosphines

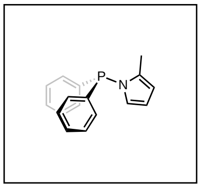

***N*-(Diphenylphosphino)-2-methyl-1*H*-pyrrole (L1)**. Lithium 2-methylpyrrolide (122 mg, 1.40 mmol, 1.0 equiv) was suspended in diethyl ether (10 mL). Ph_2PCl (307 mg, 0.25 mL, 1.4 mmol, 1.0 equiv) was added to the stirred solution via syringe at room temperature, resulting in a turbid reaction mixture. The reaction mixture was stirred for 2 h and then passed through a syringe filter to remove lithium chloride. The ether solution was concentrated to an oil. The oil was dissolved in hexane (10 mL) and passed through a pad of silica (approx. 1-cm thick). Hexane was removed *in vacuo* to yield a colorless oil. Pentane (~ 1 mL) was added to the oil and allowed to stand at -35 °C overnight. Following decantation of the pentane and exposure to vacuum, a colorless solid was isolated (114 mg, 0.430 mmol, 31% yield). *Note:* Higher yields were obtained with thinner pads of silica; however, impurities totaling up to 5% of ^{31}P content remained. Use of the analogous sodium and potassium pyrrolides and other purification methods resulted in similar outcomes. Filtration through silica must be conducted under an inert atmosphere. **^{1}H NMR** (500 MHz, C_6D_6): δ 7.28 – 7.18 (m, 4H, C_6H_5), 7.05 – 6.96 (overlapping resonances, 6H, C_6H_5), 6.51 (m, 1H, pyrrole), 6.31 (m, 1H, pyrrole), 6.17 (m, 1H, pyrrole), 2.42 (t, *J* = 1.2 Hz, 3H, CH_3); **^{13}C{^{1}H} NMR** (126 MHz, C_6D_6): δ 137.6 (d, *J* = 13.8 Hz), 134.9 (d, *J* = 20.3 Hz), 132.5 (d, *J* = 21.1 Hz), 129.7, 128.9 (d, *J* = 6.4 Hz), 123.9 (d, *J* = 5.1 Hz), 111.6, 110.4 (d, *J* = 2.6 Hz), 13.6 (d, *J* = 17.3 Hz); **^{31}P{^{1}H} NMR** (202 MHz, C_6D_6): δ 36.7; **HRMS** (ESI, TOF): exact mass calculated for $C_{17}H_{17}NP$ $[M+H]^+$ 266.1093, found 266.1046.

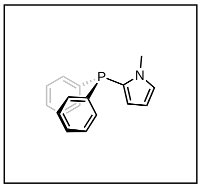

2-(Diphenylphosphino)-1-methyl-1*H*-pyrrole (**L2**). The title compound was prepared using a modified version of literature procedures.[2,3] A 50-mL Schlenk flask under nitrogen on a Schlenk line was sequentially charged with *N*-methylpyrrole (1.30 mL, 14.6 mmol, 1.2 equiv), TMEDA (1.9 mL, 12.7 mmol, 1.0 equiv), and *n*-BuLi (8 mL of 1.6 M solution in hexane, 12.8 mmol, 1.0 equiv). The reaction mixture changed from colorless to yellow upon addition of *n*-BuLi. The solution was heated at 50 °C for 30 min. The flask was then cooled to -78 °C and Ph_2PCl (2.2 mL, 12.3 mmol, 1.0 equiv) was added dropwise. The resulting viscous, orange reaction mixture was warmed to ambient temperature and stirred for 19 h. The reaction mixture was opened to air and filtered through a thin pad of silica, which was washed with diethyl ether (2 × 10 mL) to yield a pale yellow filtrate. Solvent was removed by rotary evaporation and the resulting oil was distilled by Kugelrohr distillation (145 °C, approx. 100 mtorr) to yield a pale yellow oil that solidifies upon standing (2.349 g, 8.85 mmol, 72% yield). *Note*: Full NMR characterization data were not previously reported in the literature and so are provided here. The yield is not optimized. **^{1}H NMR** (400 MHz, $CDCl_3$): δ 7.37 – 7.31 (overlapping resonances, 10H, C_6H_5), 6.88 (m, 1H, pyrrole), 6.19 (m, 1H, pyrrole), 5.91 (m, 1H, pyrrole), 3.65 (s, 3H, CH_3); **^{13}C{^{1}H} NMR** (101 MHz, $CDCl_3$): δ 137.0 (d, *J* = 6.5 Hz), 133.4 (d, *J* = 19.5 Hz), 128.7, 128.6 (d, *J* = 7.0 Hz), 127.5, 126.8 (d, *J* = 2.5 Hz), 118.6 (d, *J* = 3.2 Hz), 108.6 (d, *J* = 2.8 Hz), 35.4 (d, *J* = 13.5 Hz) (*Note*: The resonances at 128.7 and 128.6 were distinguished by acquiring spectral data at 101 and 126 MHz); **^{31}P{^{1}H} NMR** (162 MHz, $CDCl_3$): δ -29.9; **HRMS** (ESI, TOF): exact mass calculated for $C_{17}H_{17}NP$ $[M+H]^+$ 266.1093, found 266.1067.

IV. Synthesis and Characterization of Phosphine Selenides

Selenium (12.2 mg, 0.155 mmol, 1.6 equiv) and phosphine (0.100 mmol, 1.0 equiv) were stirred in toluene (3 mL) at ambient temperature for the indicated time in a 20-mL scintillation vial. The reaction mixture was then filtered through a syringe filter to remove unreacted selenium, and the resulting solution was concentrated under vacuum. The crude material was crystallized by layering a toluene solution of the product with pentane in the glovebox to yield colorless crystals in all cases.

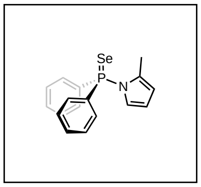

L1^{Se}. The general procedure was followed using *N*-(diphenylphosphino)-2-methyl-1*H*-pyrrole (26.5 mg, 0.100 mmol, 1.0 equiv) and the reaction mixture was stirred for 22 h. The product was isolated as colorless crystals (18.6 mg, 0.054 mmol, 54% yield). **^{1}H NMR** (500 MHz, CD_2Cl_2) δ 7.80 – 7.71 (m, 4H, C_6H_5), 7.63 – 7.57 (m, 2H, C_6H_5), 7.55 – 7.48 (m, 4H, C_6H_5), 6.20 (ddd, *J* = 4.3, 3.0, 1.5 Hz, 1H, pyrrole), 6.13 – 6.02 (overlapping resonances, 2H, pyrrole), 2.17 (s, 3H, CH_3); **^{13}C{^{1}H} NMR** (126 MHz, CD_2Cl_2): δ 135.1 (d, *J* = 4.8 Hz), 133.0 (d, *J* = 3.2 Hz), 132.8 (d, *J* = 11.9 Hz), 132.2 (d, *J* = 90.6 Hz), 129.3 (d, *J* = 13.5 Hz), 125.3 (d, *J* = 5.1 Hz), 114.4 (d, *J* = 6.4 Hz), 110.7 (d, *J* = 8.1 Hz), 15.5 (d, *J* = 2.3 Hz); **^{31}P{^{1}H} NMR** (202 MHz, CD_2Cl_2): δ 54.8 ($J_{P–Se}$ = 813 Hz); **HRMS** (ESI, TOF): exact mass calculated for $C_{17}H_{16}NPSeNa$ $[M+Na]^+$ 368.0084, found 368.0085.

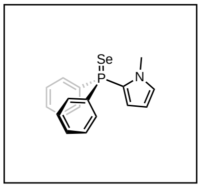

L2^{Se}.[4] The general procedure was followed using 2-(diphenylphosphino)-1-methyl-1*H*-pyrrole (26.5 mg, 0.100 mmol, 1.0 equiv) and the reaction mixture was stirred for 25 h. The product was isolated as colorless crystals (19.2 mg, 0.056 mmol, 56% yield). *Note*: Full NMR characterization data for this compound were previously not in the literature and so are provided here. **^{1}H NMR** (400 MHz, CD_2Cl_2): δ 7.83 – 7.74 (m, 4H, C_6H_5), 7.58 – 7.43 (overlapping resonances, 6H, C_6H_5), 6.95 (m, 1H, pyrrole), 6.10 (m, 1H, pyrrole), 5.83 (td, *J* = 3.7, 1.7 Hz, 1H, pyrrole), 3.70 (s, 3H, CH_3); **^{13}C{^{1}H} NMR** (101 MHz, CD_2Cl_2): δ 132.9 (d, *J* = 11.4 Hz), 132.2 (d, *J* = 3.2 Hz), 132.0 (d, *J* = 80.6 Hz), 131.2 (d, *J* = 6.9 Hz), 128.9 (d, *J* = 12.8 Hz), 122.0 (d, *J* = 14.3 Hz), 120.3 (d, *J* = 103.5 Hz), 108.3 (d, *J* = 11.9 Hz), 36.9 (d, *J* = 2.0 Hz); **^{31}P{^{1}H} NMR** (162 MHz, CD_2Cl_2): δ 16.8 ($J_{P–Se}$ = 726 Hz); **HRMS** (ESI, TOF): exact mass calculated for $C_{17}H_{16}NPSeNa$ $[M+Na]^+$ 368.0084, found 368.0072.

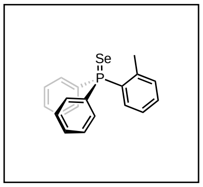

L3^{Se}.[5] The general procedure was followed using diphenyl(*o*-tolyl)phosphine (27.5 mg, 0.100 mmol, 1.0 equiv) and the reaction mixture was stirred for 22 h. The product was isolated as colorless crystals (18.7 mg, 0.053 mmol, 53% yield). *Note*: Full NMR characterization data for this compound were not previously reported in the literature and so are provided here. **^{1}H NMR** (400 MHz, CD_2Cl_2): δ 7.89 – 7.77 (m, 4H, C_6H_5), 7.57 – 7.44 (overlapping resonances, 6H, C_6H_5), 7.41 (tt, *J* = 7.5, 1.7 Hz, 1H, C_6H_4Me), 7.29 (m, 1H, C_6H_4Me), 7.14 (m, 1H, C_6H_4Me), 6.92 (ddd, *J* = 14.9, 7.8, 1.4 Hz, 1H, C_6H_4Me), 2.36 (s, 3H, CH_3); **^{13}C{^{1}H} NMR** (126 MHz, CD_2Cl_2): δ 142.9 (d, *J* = 9.7 Hz), 133.2 (d, *J* = 10.9 Hz), 133.1 (d, *J* = 10.7 Hz), 133.0 (d, *J* = 11.3 Hz), 132.1 (d, *J* = 3.0 Hz), 132.0 (d, *J* = 3.1 Hz), 131.5 (d, *J* = 75.4 Hz), 130.9 (d, *J* = 76.9 Hz), 129.0 (d, *J* = 12.4 Hz), 126.0 (d, *J* = 12.4 Hz), 22.3 (d, *J* = 5.6 Hz); **^{31}P{^{1}H} NMR** (162 MHz, CD_2Cl_2): δ 31.9 ($J_{P–Se}$

= 730 Hz); **HRMS** (ESI, TOF): exact mass calculated for $C_{19}H_{17}PSeNa$ $[M+Na]^+$ 379.0126, found 379.0120.

V. Synthesis and Characterization of Complexes (η^6-*p*-cymene)Ru(L)Cl_2

General procedure for the synthesis of (η^6-*p*-cymene)Ru(L)Cl_2

[(*p*-cymene)Ru(Cl)(μ-Cl)]$_2$ (30.6 mg, 0.050 mmol, 1.0 equiv) was dissolved in DCM (2 mL) in a scintillation vial. A solution of the phosphine (0.10 mmol, 1.0 equiv) in DCM (1 mL) was added, and the resulting solution was stirred for 1 h at 23 °C. The reaction mixture was removed from the glovebox, filtered, reduced in volume, and then layered with pentane. The resulting crystals were washed with pentane (~1 mL) and residual solvent was removed under vacuum to yield the desired product.

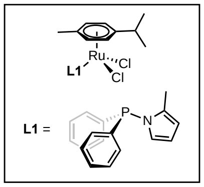

The general procedure was followed using *N*-(diphenylphosphino)-2-methyl-1*H*-pyrrole (26.5 mg, 0.100 mmol). The product was isolated as dark red crystals (39.2 mg, 0.069 mmol, 69% yield). **^{1}H NMR** (500 MHz, CD_2Cl_2): δ 7.71 – 7.65 (m, 4H, C_6H_5), 7.47 – 7.34 (overlapping resonances, 6H, C_6H_5), 7.09 (m, 1H, pyrrole), 6.24 (m, 1H, pyrrole), 6.17 (m, 1H, pyrrole), 5.38 (d, *J* = 6.2 Hz, 2H, *p*-cymene), 4.98 (d, *J* = 5.3 Hz, 2H, *p*-cymene), 2.88 (sept, *J* = 6.9 Hz, 1H, C*H*(CH_3)$_2$), 1.90 (s, 3H, C*H*$_3$), 1.74 (d, *J* = 1.0 Hz, 3H, C*H*$_3$), 1.27 (d, *J* = 7.0 Hz, 6H, CH(C*H*$_3$)$_2$); **^{13}C{^{1}H} NMR** (126 MHz, CD_2Cl_2): δ 134.9, 133.7, 133.4 (d, *J* = 10.3 Hz), 131.2 (d, *J* = 2.7 Hz), 129.5 (d, *J* = 10.8 Hz), 128.2 (d, *J* = 10.5 Hz), 114.4 (d, *J* = 3.7 Hz), 112.8 (d, *J* = 4.7 Hz), 110.0 (d, *J* = 7.9 Hz), 99.7, 89.4 (d, *J* = 5.2 Hz), 88.6 (d, *J* = 2.9 Hz), 31.0, 22.3, 18.2, 15.7 (d, *J* = 1.6 Hz); **^{31}P{^{1}H} NMR** (202 MHz, CD_2Cl_2): δ 68.1; **EA**: Anal. Calcd. for $C_{27}H_{30}Cl_2NPRu$: C, 56.75; H, 5.29; N: 2.45. Found: C, 56.47; H, 5.16; N, 2.45.

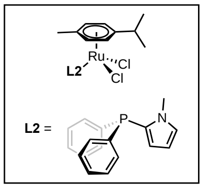

The general procedure was followed using 2-(diphenylphosphino)-1-methyl-1*H*-pyrrole (26.5 mg, 0.100 mmol). The product was isolated as dark red crystals (38.8 mg, 0.069 mmol, 69% yield). **^{1}H NMR** (500 MHz, CD_2Cl_2): δ 7.73 (ddd, *J* = 11.1, 8.4, 1.4 Hz, 4H, C_6H_5), 7.42 – 7.29 (overlapping resonances, 6H, C_6H_5), 7.01 (m, 1H, pyrrole), 6.39 (m, 1H, pyrrole), 6.25 (m, 1H, pyrrole), 5.35 (d, *J* = 6.0 Hz, 2H, *p*-cymene), 4.91 (d, *J* = 6.2, 2H, *p*-cymene), 3.44 (s, 3H, C*H*$_3$), 2.92 (sept, *J* = 6.9 Hz, 1H, C*H*(CH_3)$_2$), 1.81 (s, 3H, C*H*$_3$), 1.28 (d, *J* = 6.9 Hz, 6H, CH(C*H*$_3$)$_2$); **^{13}C{^{1}H} NMR** (126 MHz, CD_2Cl_2): δ 134.8 (d, *J* = 9.8 Hz), 132.7 (d, *J* = 48.2 Hz), 130.6 (d, *J* = 2.7 Hz), 130.1 (d, *J* = 4.3 Hz), 128.1 (d, *J* = 10.3 Hz), 124.8 (d, *J* = 59.9 Hz), 123.2 (d, *J* = 12.4 Hz), 113.1 (d, *J* = 5.4 Hz), 108.7 (d, *J* = 9.2 Hz), 98.9, 88.5 (d, *J* = 5.1 Hz), 87.0 (d, *J* = 2.4 Hz), 37.9 (d, *J* = 1.7 Hz), 30.9, 22.2, 17.9; **^{31}P{^{1}H} NMR** (202 MHz, CD_2Cl_2): δ 11.9; **EA**: Anal. Calcd. for $C_{27}H_{30}Cl_2NPRu$: C, 56.75; H, 5.29; N: 2.45. Found: C, 55.20; H, 5.19; N, 2.46. *Note:* NMR data support the purity and identity of this complex despite unsatisfactory combustion data.

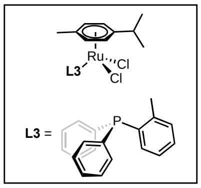

The general procedure was followed using diphenyl(*o*-tolyl)phosphine (27.6 mg, 0.100 mmol). The product was isolated as dark red crystals with 0.5 equiv of co-crystallized DCM (40.0 mg, 0.064, 64% yield). **^{1}H NMR** (500 MHz, CD_2Cl_2): δ 7.71 – 7.62 (m, 4H, aromatic), 7.53 – 7.44 (overlapping resonances, 2H, aromatic), 7.42 – 7.25 (overlapping resonances, 8H, aromatic), 5.31 (d, *J* = 6.1 Hz, 2H, *p*-cymene), 4.82 (d, *J* = 5.0 Hz, 2H, *p*-cymene), 2.94 (sept, *J* = 6.9 Hz, 1H, C*H*(CH_3)$_2$), 2.13 (s, 3H, C*H*$_3$), 1.82 (s, 3H, C*H*$_3$), 1.28 (d, *J* = 7.0 Hz, 6H, CH(C*H*$_3$)$_2$); **^{13}C{^{1}H} NMR** (126 MHz, CD_2Cl_2): δ 143.60 (d, *J* = 6.9 Hz), 136.61 (d, *J* = 13.1 Hz), 134.95 (d, *J* = 9.2

Hz), 132.92 (d, J = 42.0 Hz), 132.61 (d, J = 7.4 Hz), 132.25 (d, J = 45.4 Hz), 131.48 (d, J = 2.3 Hz), 130.35 (d, J = 2.6 Hz), 128.02 (d, J = 10.1 Hz), 125.82 (d, J = 10.5 Hz), 111.88 (d, J = 4.9 Hz), 98.55, 88.60 (d, J = 4.8 Hz), 87.42 (d, J = 2.6 Hz), 30.95, 23.80 (d, J = 4.5 Hz), 22.30, 18.12; **$^{31}P\{^1H\}$ NMR** (202 MHz, CD_2Cl_2): δ 28.83; **EA**: Anal. Calcd. for $C_{29}H_{31}Cl_2PRu$•$0.5CH_2Cl_2$: C, 56.69; H: 5.16; N: 0.00. Found: C, 54.11; H, 4.92; N, 0.00 (*Note*: Co-crystallization of 0.5 equiv DCM was corroborated by ^1H NMR in three independent syntheses. Combustion analysis was consistently low in carbon despite multiple attempts. NMR data support the purity and identity of this complex despite unsatisfactory combustion data.).

VI. Synthesis and Characterization of Complexes *trans*-(L)$_2$Rh(CO)Cl

General procedure for the synthesis of *trans*-L$_2$Rh(CO)Cl

[Rh(CO)$_2$Cl]$_2$ (19.4 mg, 0.050 mmol, 1.0 equiv) was dissolved in toluene (1 mL). To the mixture was added a solution of the ligand (0.20 mmol, 4.0 equiv) in toluene (2 mL). The reaction mixture was stirred at 23 °C for 30 minutes, during which time a yellow precipitate formed. The reaction mixture was then concentrated, and the crude material was crystallized at 23 ºC by layering a DCM solution of the product with pentane.

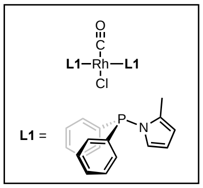

***trans*-(L1)$_2$Rh(CO)Cl.** The general procedure was followed using *N*-(diphenylphosphino)-2-methyl-1*H*-pyrrole (53.0 mg, 0.200 mmol, 4.0 equiv). The product was isolated as yellow crystals (35.9 mg, 0.052 mmol, 52% yield). X-ray quality crystals were grown by layering a DCM solution of the product with pentane at room temperature. **^{1}H NMR** (400 MHz, CD$_2$Cl$_2$): δ 7.74 – 7.65 (overlapping aryl resonances, 8H, C$_6$*H*$_5$), 7.56 – 7.50 (overlapping resonances, 4H, C$_6$*H*$_5$), 7.50 – 7.43 (overlapping resonances, 8H, C$_6$*H*$_5$), 6.49 (p, *J* = 2.1 Hz, 2H, pyrrole), 6.14 – 6.09 (overlapping resonances, 4H, pyrrole), 2.40 (s, 6H, C*H*$_3$); **^{13}C{^{1}H} NMR** (101 MHz, CD$_2$Cl$_2$): δ 134.6 (vt, *J* = 3.1 Hz), 134.1 (vt, *J* = 7.2 Hz), 132.7 (dvt, *J* = 24.5, 1.4 Hz), 131.8, 128.9 (vt, *J* = 5.3 Hz), 126.0 (vt, *J* = 2.3 Hz), 112.9 (vt, *J* = 2.2 Hz), 110.4 (vt, *J* = 3.2 Hz), 16.0 (vt, *J* = 2.5 Hz) (*Note*: The carbonyl resonance was not detected); **^{31}P{^{1}H} NMR** (162 MHz, CD$_2$Cl$_2$): δ 67.8 (d, $J_{P–Rh}$ = 138.9 Hz); **IR** (ATR, cm^{-1}): 1,986; **EA**: Anal. Calcd. for C$_{35}$H$_{32}$ClN$_2$OP$_2$Rh: C, 60.32; H, 4.63; N, 4.02. Found: C, 60.05; H, 4.55; N, 4.00.

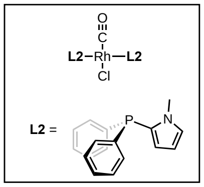

***trans*-(L2)$_2$Rh(CO)Cl.** The general procedure was followed using 2-(diphenylphosphino)-1-methyl-1*H*-pyrrole (53.0 mg, 0.200 mmol, 4.0 mmol). The product was isolated as yellow crystals, which were subsequently dissolved in DCM and exposed to vacuum to remove co-crystallized DCM. (48.2 mg, 0.069 mmol, 69% yield). X-ray quality crystals were grown by layering a DCM solution of the product with hexane at room temperature. **^{1}H NMR** (500 MHz, CD$_2$Cl$_2$): δ 7.79 – 7.71 (overlapping resonances, 8H, C$_6$*H*$_5$), 7.50 – 7.38 (overlapping resonances, 12H, C$_6$*H*$_5$), 6.94 (m, 2H, pyrrole), 6.13 (m, 2H, pyrrole), 5.98 (m, 2H, pyrrole), 3.80 (s, 6H, C*H*$_3$); **^{13}C NMR** (126 MHz, CD$_2$Cl$_2$): δ 134.8 (vt, *J* = 6.6 Hz), 133.1 (vt, *J* = 24.4 Hz), 130.7, 129.0 (vt, *J* = 2.2 Hz, 128.7 (vt, *J* = 5.2 Hz), 122.8 (vt, *J* = 32.8 Hz), 120.9 (vt, *J* = 5.5 Hz), 108.6 (vt, *J* = 4.4 Hz), 37.5 (*Note*: The carbonyl resonance was not detected); **^{31}P NMR** (202 MHz, CD$_2$Cl$_2$): δ 10.6 (d, $J_{P–Rh}$ = 124.3 Hz); **IR** (ATR, cm^{-1}): 1,966; **EA**: Anal. Calcd. for C$_{35}$H$_{32}$ClN$_2$OP$_2$Rh: C, 60.32; H, 4.63; N, 4.02. Found: C, 60.12; H, 4.59; N, 4.00.

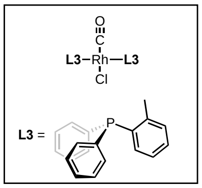

***trans*-(L3)$_2$Rh(CO)Cl.** The general procedure was followed using diphenyl(*o*-tolyl)phosphine (55.3 mg, 0.200 mmol, 4.0 equiv). The product was isolated as yellow crystals with 0.3 equiv of co-crystallized DCM (50.1 mg, 0.070 mmol, 67% yield). *Note*: This compound is reported in the literature but was not fully characterized.[6,7] X-ray quality crystals were grown by layering a DCM solution of the product with pentane at room temperature. **^{1}H NMR** (500 MHz, CD$_2$Cl$_2$): δ 7.82 – 7.72 (overlapping resonances, 8H, C$_6$*H*$_5$), 7.55 – 7.38 (overlapping resonances, 12H, C$_6$*H*$_5$), 7.35 (td, *J* = 7.5, 1.3 Hz, 2H, C$_6$*H*$_4$Me), 7.28 (dd, *J* = 7.7, 2.0 Hz, 2H, C$_6$*H*$_4$Me), 7.11 (t, *J*

= 7.5 Hz, 2H, C_6H_4Me), 6.92 (m, 2H, C_6H_4Me), 2.65 (s, 6H, CH_3); **^{13}C{^{1}H} NMR** (126 MHz, CD_2Cl_2): δ 142.0 (vt, *J* = 6.5 Hz), 135.7 (vt, *J* = 6.5 Hz), 133.1 (vt, *J* = 3.7 Hz), 132.4 (vt, *J* = 22.7 Hz), 131.6 (vt, *J* = 3.8 Hz), 130.7, 130.5, 128.7 (vt, *J* = 5.1 Hz), 125.8 (vt, *J* = 4.0 Hz), 23.72 (vt, *J* = 5.2 Hz) (*Note*: The carbonyl resonance was poorly resolved and one aromatic resonance was not detected); **^{31}P{^{1}H} NMR** (202 MHz, CD_2Cl_2): δ 25.22 (d, $J_{P–Rh}$ = 125.3 Hz); **IR** (ATR, cm^{-1}): 1,969; **EA**: Anal. Calcd. for $C_{39}H_{34}ClOP_2Rh$•$0.3CH_2Cl_2$: C, 63.40; H: 4.68; N. 0.00. Found: C, 62.39; H, 4.70; N, 0.00. (*Note*: Co-crystallization of 0.3 equiv DCM was corroborated by ^{1}H NMR in two independent syntheses and combustion analyses).

VII. Synthesis and Characterization of Complexes *cis*-$(L)_2PtCl_2$

General procedure for the synthesis of *cis*-L_2PtCl_2

Pt(cod)Cl_2 (18.7 mg, 0.050 mmol, 1.0 equiv) was dissolved in DCM (1 mL) in a 20-mL scintillation vial. A solution of the phosphine (0.10 mmol, 2.0 equiv) in DCM (2 mL) was added. The resulting solution was stirred for 1 h at 23 °C. The solvent was removed in vacuo and the resulting crude material was washed with pentane (~1 mL) and crystallized as described below. The resulting crystals were washed with pentane and residual solvent was removed to yield the desired product.

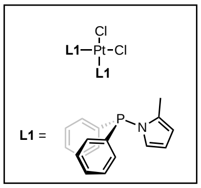

$(L1)_2PtCl_2$. The general procedure was followed using *N*-(diphenylphosphino)-2-methyl-1*H*-pyrrole (26.5 mg, 0.10 mmol, 2.0 equiv). Impure material was always isolated despite attempting multiple purification methods and syntheses of the title compound. *Note*: The NMR data provided are of the purest sample obtained after three crystallizations of a DCM solution layered with pentane. **^{1}H NMR** (400 MHz, CD_2Cl_2): δ 7.50 – 7.40 (m, 4H, C_6H_5), 7.39 – 7.29 (overlapping resonances, 8H, C_6H_5), 7.29 – 7.22 (overlapping resonances, 8H, C_6H_5), 7.16 (m, 2H, pyrrole), 6.14 (q, *J* = 2.9 Hz, 2H, pyrrole), 6.04 (m, 2H, pyrrole), 1.75 (s, 6H, CH_3); **^{13}C{^{1}H}** NMR (101 MHz, CD_2Cl_2): δ 135.0, 134.1 (vt, *J* = 6.0 Hz), 132.2, 129.9 – 129.0 (overlapping resonances), 128.7 (vt, *J* = 6.0 Hz), 114.5 (vt, *J* = 2.7 Hz), 110.8 (vt, *J* = 4.5 Hz), 16.0; **^{31}P{^{1}H}** NMR (162 MHz, CD_2Cl_2): δ 41.2 (s with Pt satellites, $J_{P\text{–}Pt}$ = 3,934 Hz); **EA**: Not performed. Pure material was not isolated.

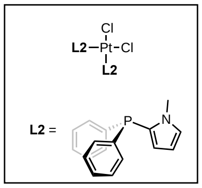

$(L2)_2PtCl_2$. The general procedure was followed using 2-(diphenylphosphino)-1-methyl-1*H*-pyrrole (26.5 mg, 0.10 mmol, 2.0 equiv). The product was isolated as faint pink crystals (31.2 mg, 0.039 mmol, 78% yield) by diffusion of pentane into a chloroform solution of the title compound. X-ray quality crystals were grown by layering a DCM solution of the title compound with pentane at room temperature. *Note*: This complex was previously prepared but not fully characterized.[3] **^{1}H NMR** (500 MHz, CD_2Cl_2): δ 7.47 – 7.33 (overlapping resonances, 12H, C_6H_5), 7.18 (overlapping resonances, 8H, C_6H_5), 6.82 (m, 2H, pyrrole), 6.14 (m, 2H, pyrrole), 6.07 (m, 2H, pyrrole), 3.36 (s, 6H,); **^{13}C{^{1}H} NMR** (126 MHz, CD_2Cl_2): δ 134.6 (vt, *J* = 6 Hz), 131.3, 130.2 (vt, *J* = 2.9 Hz), 129.2 (d, *J* = 68.5 Hz), 128.5 (vt, *J* = 6 Hz), 123.7 (vt, *J* = 8 Hz), 119.2 (d, *J* = 91.0 Hz), 108.5 (vt, *J* = 6 Hz), 37.7; **^{31}P{^{1}H} NMR** (202 MHz, CD_2Cl_2): δ -4.69 (s with Pt satellites, $J_{P\text{–}Pt}$ = 3,660 Hz); **EA**: Anal. Calcd. for $C_{34}H_{32}Cl_2N_2P_2Pt$: C, 51.27; H, 4.05; N, 3.52. Found: C, 49.91; H, 4.03; N, 3.40.

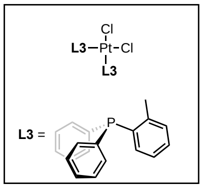

$(L3)_2PtCl_2$. The general procedure was followed using diphenyl(*o*-tolyl)phosphine (27.6 mg, 0.10 mmol, 2.0 equiv). The product was isolated as colorless crystals (25.9 mg, 0.032 mmol, 63% yield) by layering a DCM solution of the title compound with pentane. X-ray quality crystals were grown by diffusion of pentane in chloroform solution of the title compound at -3 °C. **^{1}H NMR** (500 MHz, CD_2Cl_2): δ 7.46 – 7.23 (overlapping resonances, 16H, aryl), 7.19 – 7.12 (m, 10H, aryl), 7.07 (t, *J* = 7.6 Hz, 2H, aryl), 2.30 (s, 6H); **^{13}C{^{1}H} NMR** (126 MHz, CD_2Cl_2): δ 142.6 (t, *J* = 4.4 Hz), 135.4 (t, *J* = 5.3 Hz), 134.9 (*J* = 4.8 Hz), 132.1 (*J* = 4.5 Hz),

131.3, 131.1, 128.3 (J = 5.5 Hz), 125.7 (J = 5.7 Hz), 24.2 (J = 3.3 Hz) (*Note*: Two resonances were not identified due to the low solubility of the title complex); ^{31}**P{^{1}H} NMR** (202 MHz, CD_2Cl_2): δ 9.56 (s with Pt satellites, $J_{P–Pt}$ = 3,670 Hz); **EA**: Anal. Calcd. for $C_{38}H_{34}Cl_2P_2Pt$: C, 55.75; H, 4.19; N, 0.00. Found: C, 55.08; H, 4.16; N, 0.00.

VIII. Synthesis and Characterization of Complexes (L)AuCl

General procedure for the synthesis of (L)AuCl

(DMS)AuCl (23.6 mg, 0.080 mmol, 1.0 equiv) was dissolved in THF (2 mL) in a 20-mL scintillation vial. A solution of the phosphine (0.080 mmol, 1.0 equiv) in THF (2 mL) was added. The resulting solution was stirred for 10 min at 23 °C. The solution was filtered using a syringe filter. The solvent was removed under vacuum and the residue was crystallized by slow diffusion of pentane into toluene. The resulting crystals were washed with pentane, and residual solvent was removed under vacuum to yield the desired product. X-ray quality crystals were grown using the same crystallization conditions at the indicated temperature.

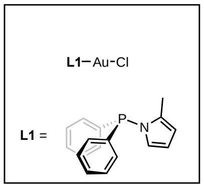

(L1)AuCl. The general procedure was followed using *N*-(diphenylphosphino)-2-methyl-1*H*-pyrrole (21.2 mg, 0.080 mmol, 1.0 equiv). The product was isolated as an off-white solid (20.5 mg, 0.041 mmol, 52% yield). X-ray quality crystals were grown at room temperature. **^{1}H NMR** (500 MHz, $CDCl_3$): δ 7.64 – 7.57 (m, 2H, C_6H_5), 7.56 – 7.43 (overlapping resonances, 8H, C_6H_5), 6.20 – 6.12 (overlapping resonances, 3H, pyrrole), 2.48 (s, 3H, C*H*$_3$); **^{13}C{^{1}H} NMR** (126 MHz, $CDCl_3$): δ 134.3 (d, *J* = 7.5 Hz), 133.4 (d, *J* = 16.2 Hz), 133.2 (d, *J* = 2.5 Hz), 129.7 (d, *J* = 12.5 Hz), 129.2 (d, *J* = 63.8 Hz), 124.6 (d, *J* = 3.0 Hz), 113.8 (d, *J* = 5.4 Hz), 111.8 (d, *J* = 7.2 Hz), 15.4 (d, *J* = 7.6 Hz); **^{31}P{^{1}H}** NMR (202 MHz, $CDCl_3$): δ 62.2. **EA**: Anal. Calcd. for $C_{17}H_{16}AuClNP$: C, 41.03; H, 3.24; N, 2.81. Found: C, 40.95; H, 3.17; N, 2.79.

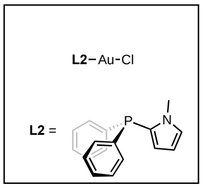

(L2)AuCl. The general procedure was followed using 2-(diphenylphosphino)-1-methyl-1*H*-pyrrole (21.2 mg, 0.080 mmol, 1.0 equiv). The product was isolated as a colorless solid (26.3 mg, 0.052 mmol, 66% yield). X-ray quality crystals were grown at -3 °C. **^{1}H NMR** (500 MHz, $CDCl_3$): δ 7.60 – 7.51 (overlapping resonances, 6H, C_6H_5), 7.51 – 7.43 (m, 4H, C_6H_5), 6.97 (dt, *J* = 4.0, 2.0 Hz, 1H, pyrrole), 6.16 (dt, *J* = 4.6, 2.5 Hz, 1H, pyrrole), 5.89 (td, *J* = 3.5, 1.7 Hz, 1H, pyrrole), 3.77 (d, *J* = 1.1 Hz, 3H, C*H*$_3$); **^{13}C{^{1}H} NMR** (126 MHz, $CDCl_3$): δ 134.1 (d, *J* = 14.5 Hz), 132.3 (d, *J* = 2.7 Hz), 130.9 (d, *J* = 5.5 Hz), 129.4 (d, *J* = 12.4 Hz), 128.5 (d, *J* = 66.7 Hz), 122.8 (d, *J* = 11.4 Hz), 117.1 (d, *J* = 85.6 Hz), 109.2 (d, *J* = 10.4 Hz), 36.8 (d, *J* = 6.5 Hz); **^{31}P{^{1}H}** NMR (202 MHz, $CDCl_3$): δ 11.2. **EA**: Anal. Calcd. for $C_{17}H_{16}AuClNP$: C, 41.03; H, 3.24; N, 2.81. Found: C, 40.15; H, 3.12; N, 2.71.

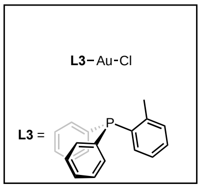

(L3)AuCl.[8] The general procedure was followed using diphenyl(*o*-tolyl)phosphine (22.1 mg, 0.080 mmol, 1.0 equiv). The product was isolated as a colorless solid (30.5 mg, 0.060 mmol, 75% yield). X-ray quality crystals were grown at -3 °C *Note*: The ^{1}H NMR data collected for this complex were in agreement with those in the literature; however, the ^{31}P resonance was previously reported at 32.1 ppm. **^{1}H NMR** (500 MHz, $CDCl_3$): δ 7.61 – 7.46 (overlapping resonances, 10H, C_6H_5), 7.43 (m, 1H, C_6H_4Me), 7.35 – 7.28 (m, 1H, C_6H_4Me), 7.22 – 7.13 (m, 1H, C_6H_4Me), 6.75 (ddd, *J* = 13.0, 7.8, 1.4 Hz, 1H, C_6H_4Me), 2.53 (s, 3H, C*H*$_3$); **^{13}C{^{1}H}** NMR (126 MHz, $CDCl_3$): δ 142.1 (d, *J* = 12.4 Hz), 134.7 (d, *J* = 13.9 Hz), 133.1 (d, *J* = 8.3 Hz), 132.3 (d, *J* = 2.7 Hz), 132.2 (d, *J* = 9.0 Hz), 132.0 (d, *J* = 2.5 Hz), 129.5 (d, *J* = 12.0 Hz), 128.1 (d, *J* = 63.4 Hz), 127.0 (d, *J* = 60.3 Hz), 126.4 (d, *J* = 10.1 Hz), 22.7 (d, *J* = 12.3 Hz); **^{31}P{^{1}H}** NMR (202

MHz, $CDCl_3$): δ 26.4. **EA**: Anal. Calcd. for $C_{19}H_{17}AuClP$: C, 44.86; H, 3.37; N, 0.00. Found: C, 44.92; H, 3.29; N, 0.00.

IX. Ligand Performance in a Model Cross-Coupling Reaction

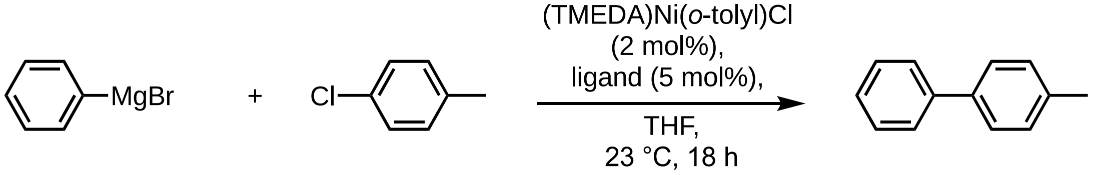

In a glovebox, a 20-mL vial was charged with (TMEDA)Ni(*o*-tolyl)Cl (8.7 mg, 0.029 mmol, 2 mol%). Ligand (0.073 mmol, 5 mol%) was suspended in THF (2 mL) and transferred to the vial. The solution was stirred for 10 minutes at room temperature to promote formation of a nickel phosphine precatalyst. 4-chlorotoluene (173 µL, 1.46 mmol, 1.0 equiv) was added to the stirred solution via syringe at room temperature. PhMgBr (2.20 mL, 2.20 mmol, 1.5 equiv) was added to the solution via syringe at room temperature, resulting in a darkened solution. The reaction mixture was stirred for 18 h and then quenched with EtOH (100 µL) open to air. Dodecane (332 µL, 1.46 mmol, 1 equiv) was added to the reaction mixture followed by EtOAc (5 mL), and the reaction mixture was stirred for 10 seconds. The mixture was filtered through a syringe filter and an aliquot was diluted in EtOAc. Yields were determined by GC-MS using dodecane as an internal standard. *Note:* The commercial (TMEDA)Ni(*o*-tolyl)Cl used was 90% pure by mass, with balance being *o*-chlorotoluene. The mass of catalyst listed is of (TMEDA)Ni(*o*-tolyl)Cl delivered to the reaction vessel.

Table S1. Yield of 4-methylbiphenyl and relative ratios of biphenyl and 4,4'-dimethylbiphenyl.

**Ligand**	**None**		**L1**		**L2**		**L3**	
**Run**	1	2	1	2	1	2	1	2
**Yield**	38	45	29	40	68	70	80	81
**Average**	42		35		69		81	

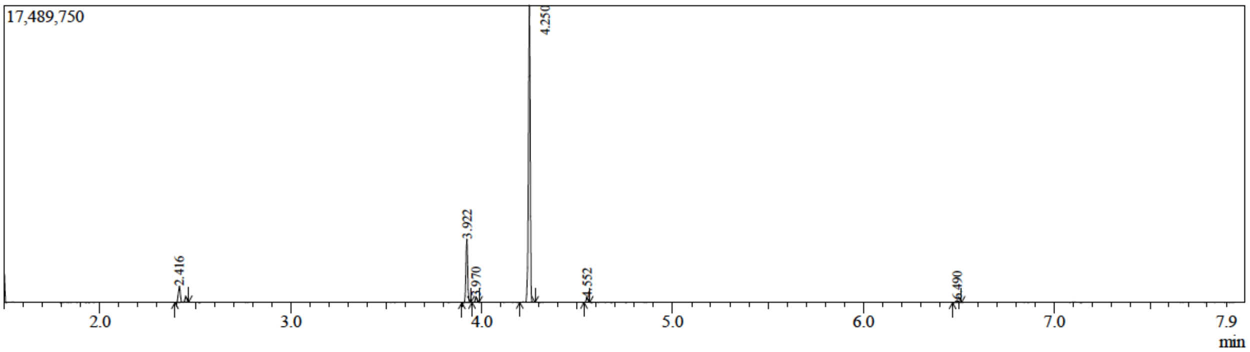

**Compound**	4-chlorotoluene	biphenyl	2-methylbiphenyl	4-methylbiphenyl	4,4'-dimethyl biphenyl	PPh_3
**Retention Time (min)**	2.416	3.922	3.970	4.250	4.552	6.490

Figure S1. Representative gas chromatogram of a catalytic reaction employing **L1** as the phosphine ligand.

X. Infrared Spectra of Complexes *trans*-(L)$_2$Rh(CO)Cl

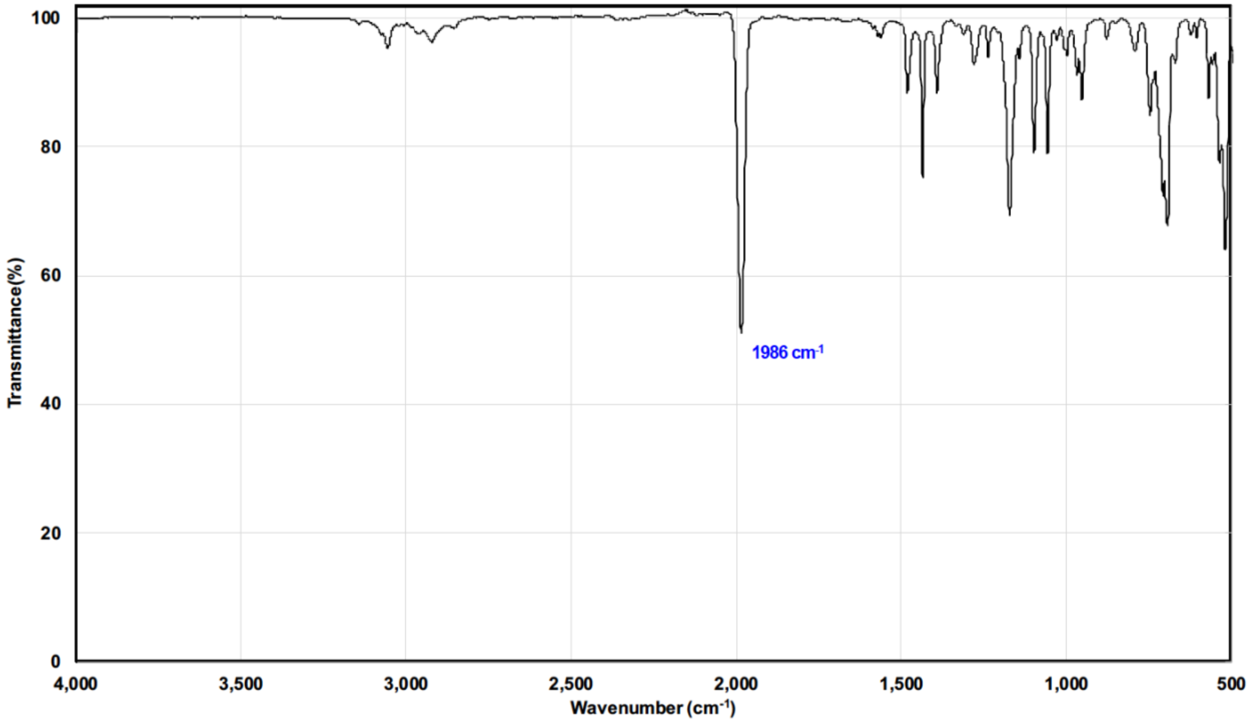

Figure S2. Infrared spectrum of *trans*-(**L1**)$_2$Rh(CO)Cl.

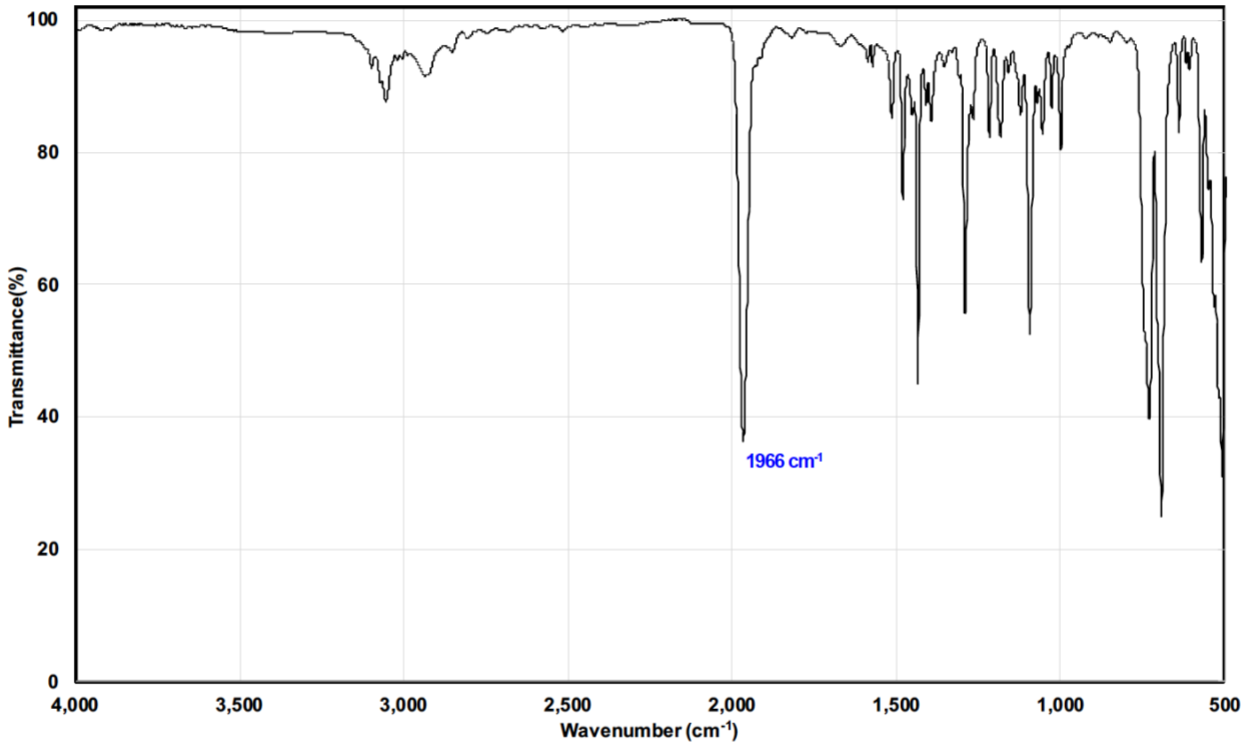

Figure S3. Infrared spectrum of *trans*-(**L2**)$_2$Rh(CO)Cl.

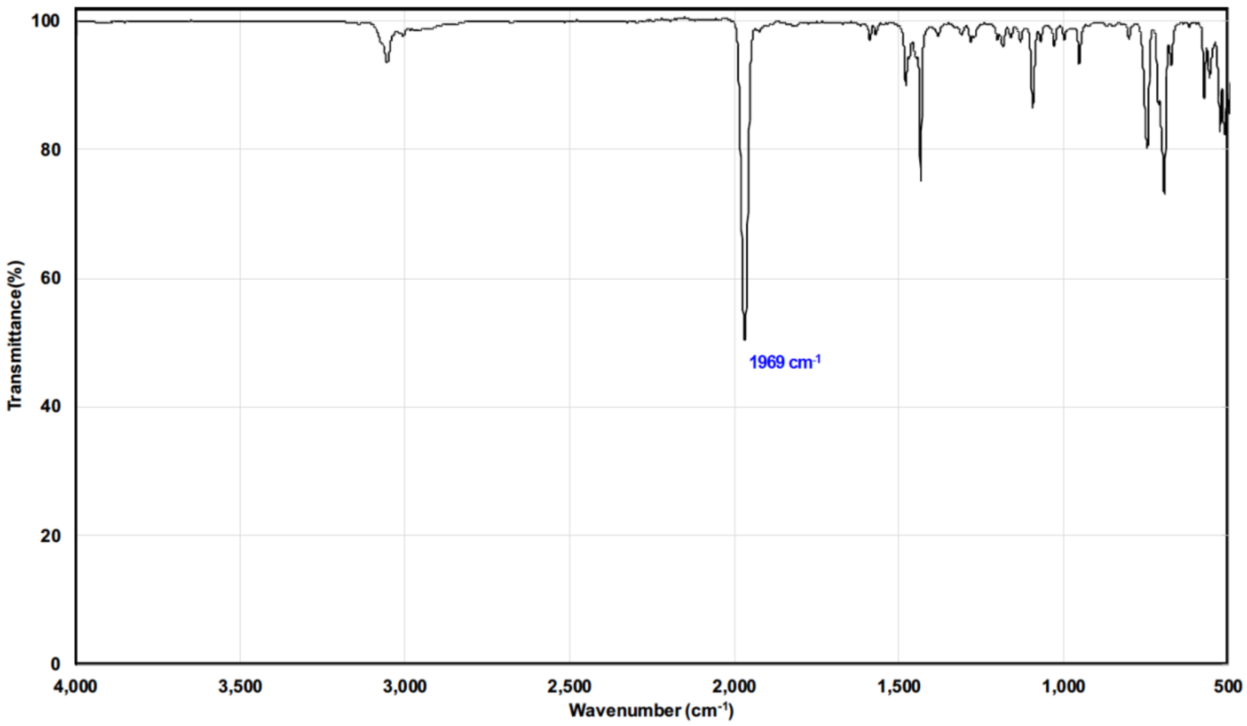

Figure S4. Infrared spectrum of *trans*-(**L3**)$_2$Rh(CO)Cl.

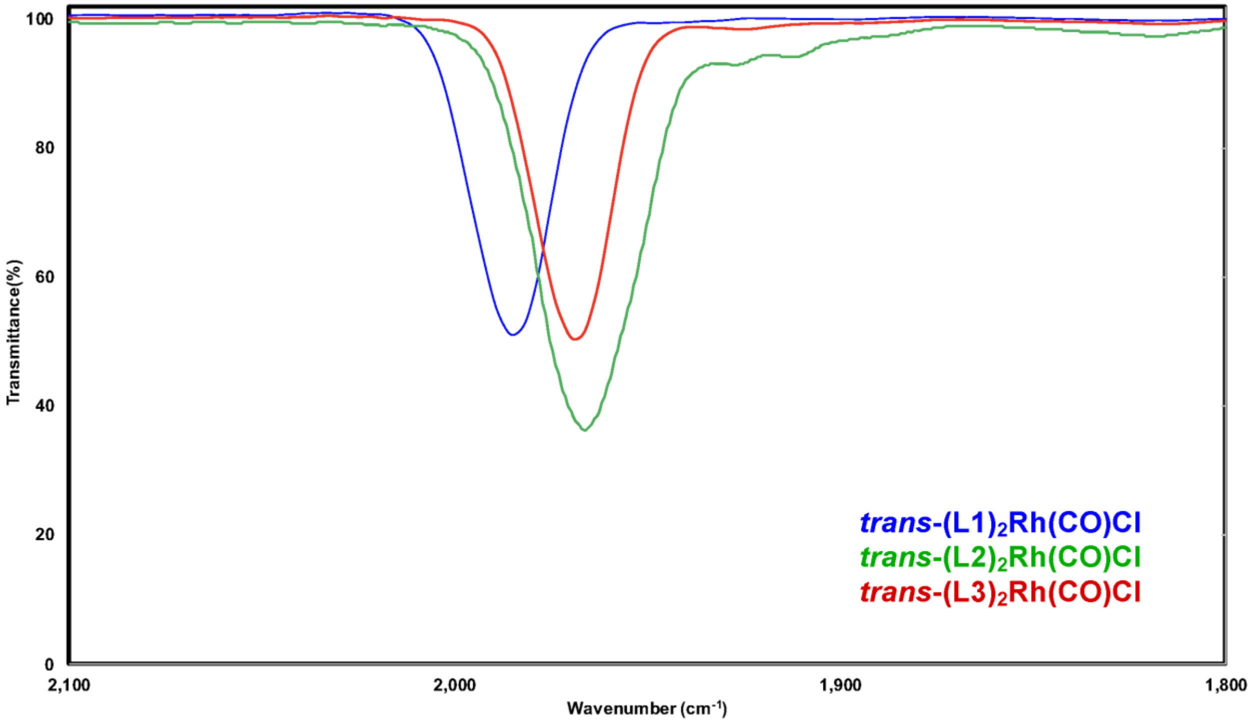

Figure S5. Superimposed spectra of *trans*-(**L**)$_2$Rh(CO)Cl (X = 1, 2, 3).

XI. Cyclic Voltammograms of (η^6-p-cymene)Ru(L)Cl_2

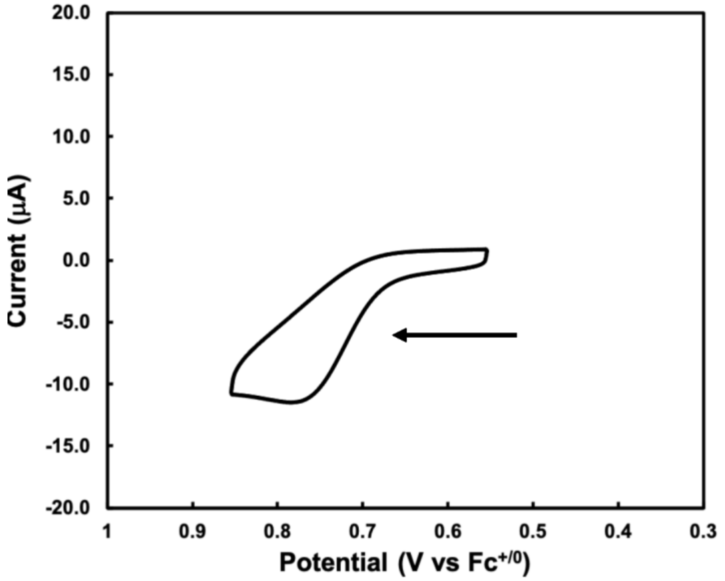

Figure S6. Cyclic voltammogram of (η^6-*p*-cymene)Ru(**L1**)Cl_2 DCM (1 mM). Conditions: scan rate: 100 mv/s; supporting electrolyte: 0.1 M [*n*-Bu_4N][PF_6]; working electrode: glass carbon; auxiliary electrode: platinum wire; reference: silver wire referenced to the $Fc^{+/0}$ couple.

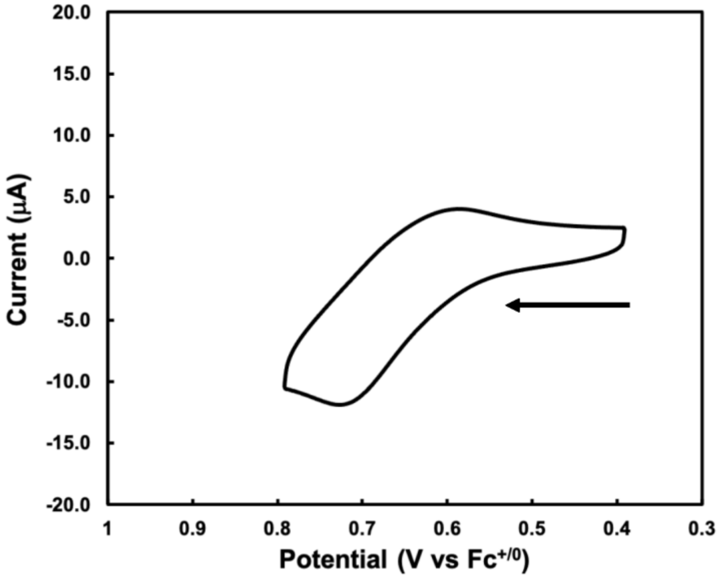

Figure S7. Cyclic voltammogram of (η^6-*p*-cymene)Ru(**L2**)Cl_2 DCM (1 mM). Conditions: scan rate: 100 mv/s; supporting electrolyte: 0.1 M [*n*-Bu_4N][PF_6]; working electrode: glass carbon; auxiliary electrode: platinum wire; reference: silver wire referenced to the $Fc^{+/0}$ couple.

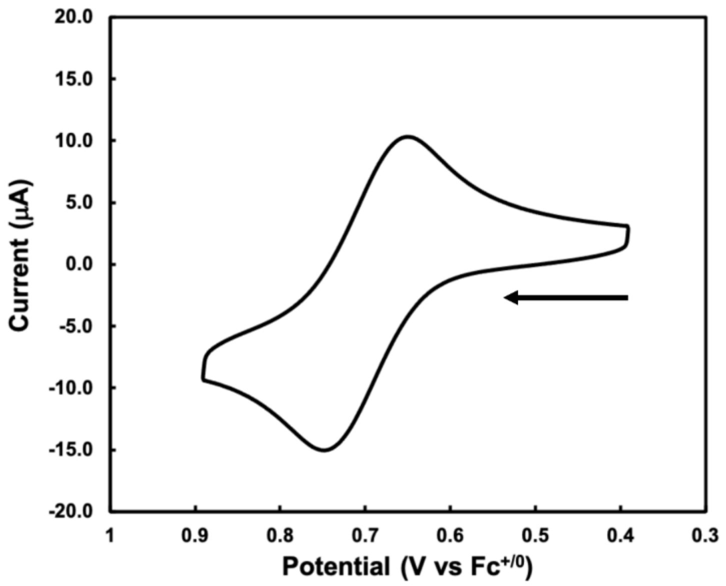

Figure S8. Cyclic voltammogram of (η^6-*p*-cymene)Ru(**L3**)Cl_2 DCM (1 mM). Conditions: scan rate: 100 mv/s; supporting electrolyte: 0.1 M [*n*-Bu_4N][PF_6]; working electrode: glass carbon; auxiliary electrode: platinum wire; reference: silver wire referenced to the $Fc^{+/0}$ couple.

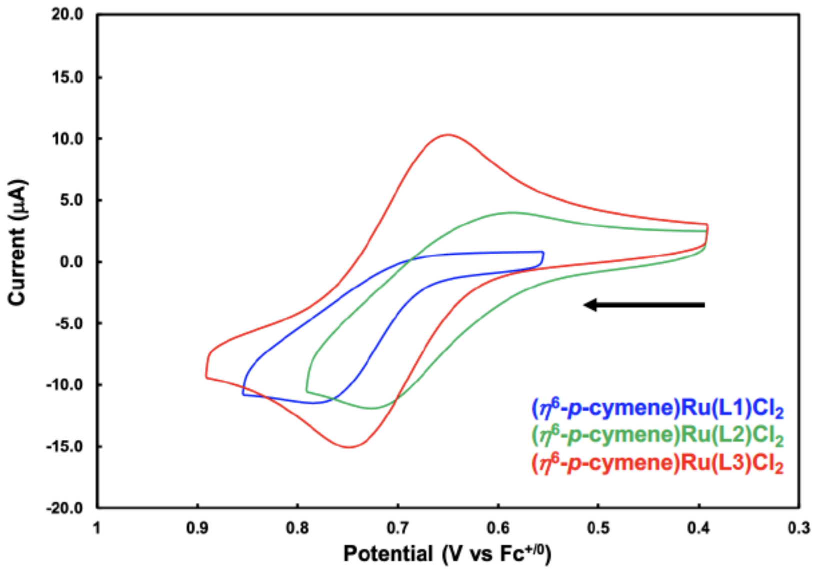

Figure S9. Superimposed cyclic voltammograms of (η^6-*p*-cymene)Ru(**L1**)Cl_2, (η^6-*p*-cymene)-Ru(**L2**)Cl_2, and (η^6-*p*-cymene)Ru(**L3**)Cl_2.

XII. Buried Volume Calculation Details for (L)AuCl

Calculations were performed using the SambVca 2.1 web application[9] based on the cartesian coordinates for the crystal structures for the complexes below. A M–P bond length of 2.28 Å was set for each complex for consistency of comparison between the three analyzed complexes and because this value is a standard distance adopted in the literature for normalizing buried volume calculations.[10]

The following parameters were used:

1. **Load file:** .cif file of complex.
2. **Select atoms coordinated to the center of the sphere:** Phosphorus atom selected.
3. **Select atoms for z-axis definition:** Gold atom selected and set to z-positive.
4. **Select atoms for the xz-plane definition:** Looking down the Au–P axis, the *ipso* carbon of the phenyl group that is counterclockwise from the non-phenyl aryl group selected.
5. **Select the atoms to be deleted:** Gold and chlorine selected.
6. **Check the chosen orientation:** Phosphorus and gold reside on the z-axis, P and *ipso* carbon define x-plane.
7. **Select the atomic radii:** Bond scaled by 1.17
8. **Sphere radius:** 3.5 Å
9. **Distance of the coordination point from the center of the sphere:** -2.28 Å
10. **Mesh spacing for numerical integration:** 0.10
11. **Inclusion of H atoms:** H atoms included

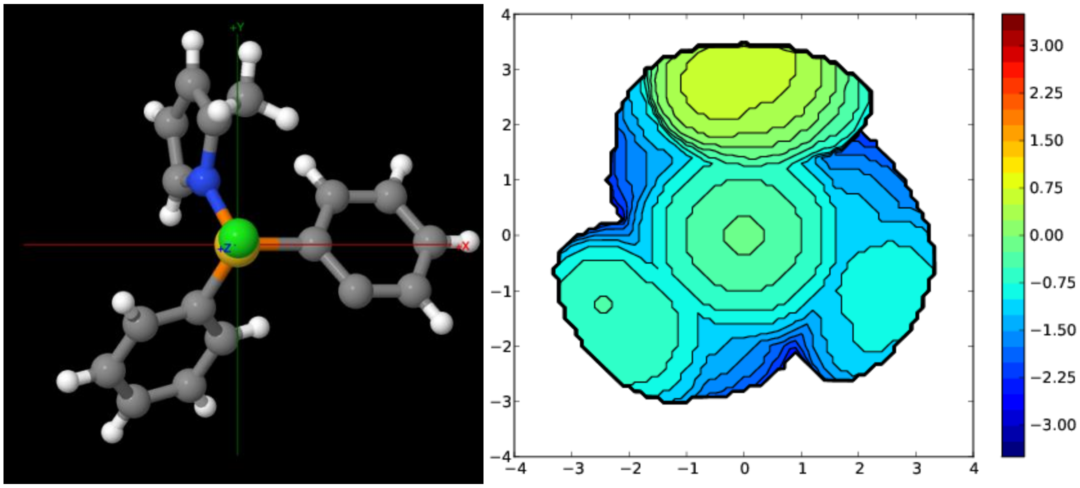

Figure S10: Steric map of (L1)AuCl; %V_{bur} = 33.9%

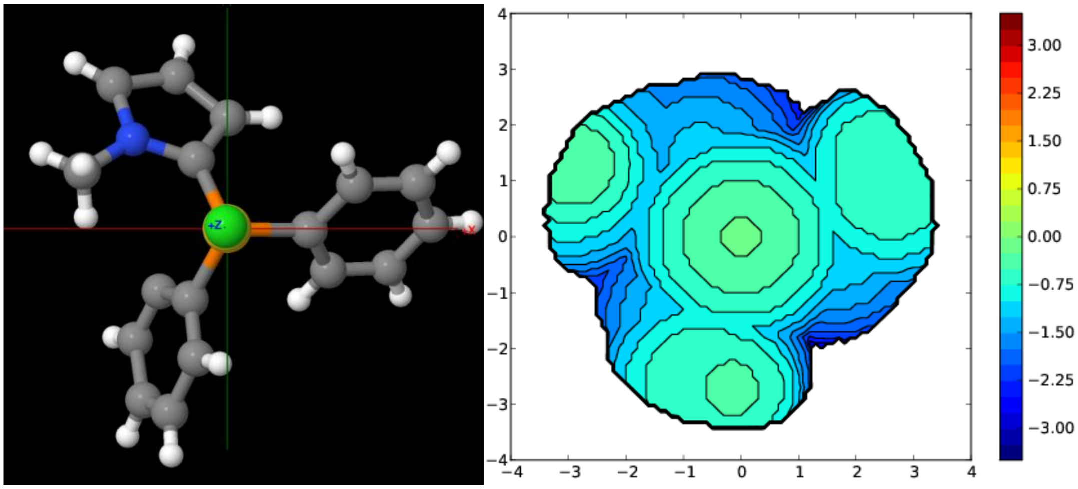

Figure S11: Steric map of (L2)AuCl; %V_{bur} = 30.4%

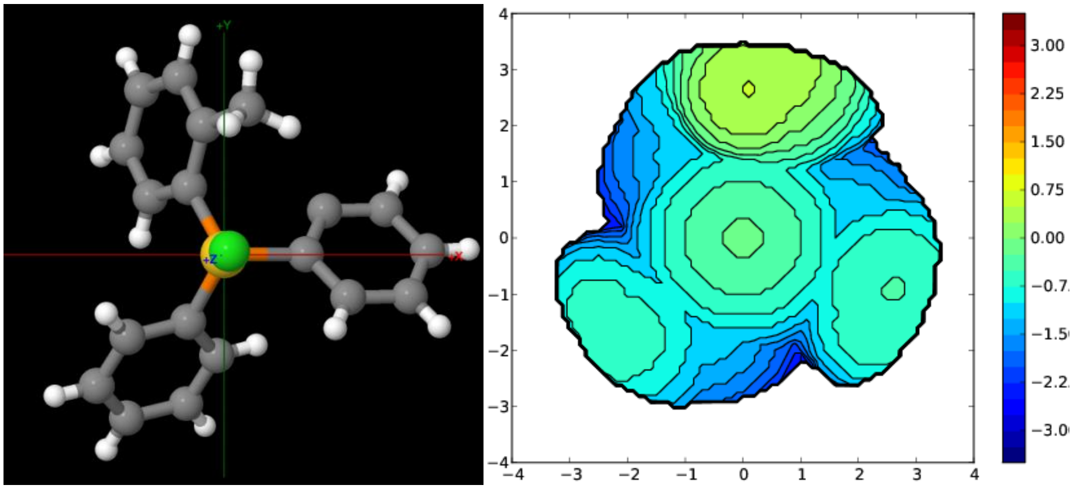

Figure S12: Steric map of (L3)AuCl; %V_{bur} = 33.8%

XIII. Summary of Comparative Data

Table S2. Comparison of key metrics

**L–X**		**L1**	**L2**	**L3**
**X** = Se	$J_{P–Se}$ (Hz)	813	726	730
**X** = *cis*-$PtCl_2L$	$d_{P–Pt}$ (Å)	-	2.276(1)<br>2.246(1)<br>2.261(1)[a]	2.271(1)<br>2.258(1)<br>2.265(1)[a]
	$J_{P–Pt}$ (Hz)	3,934	3,660	3,670
	$d_{Pt–Cl}$ (Å)	-	2.360(1)<br>2.345(1)<br>2.353(1)[a]	2.357(1)<br>2.349(1)<br>2.353(1)[a]
**X** = *trans*-Rh(CO)(Cl	ν_{CO} (cm^{-1})	1,986	1,966	1,969
	$d_{P–Rh}$ (Å)	2.3120(7)<br>2.3123(7)<br>2.3122(7)[a]	2.3258(9)<br>2.3278(9)<br>2.3289(9)[a]	2.3275(6)<br>2.3417(5)<br>2.3346(6)[a]
	$J_{Rh–P}$ (Hz)	138.9	124.3	125.3
**X** = AuCl	$\%V_{bur}$	33.9	30.4	33.8
	$d_{P–Au}$ (Å)	2.220(1)	2.243(9)	2.233(2)
	$d_{Au–Cl}$ (Å)	2.279(1)	2.295(5)	2.289(2)
**X** = (η^6-*p*-cymene)$RuCl_2$	E_{ox} (V)	0.78	0.73	0.75

[a]Average of two bond lengths.

XIV. NMR Spectra of Lithium 2-Methylpyrrolide

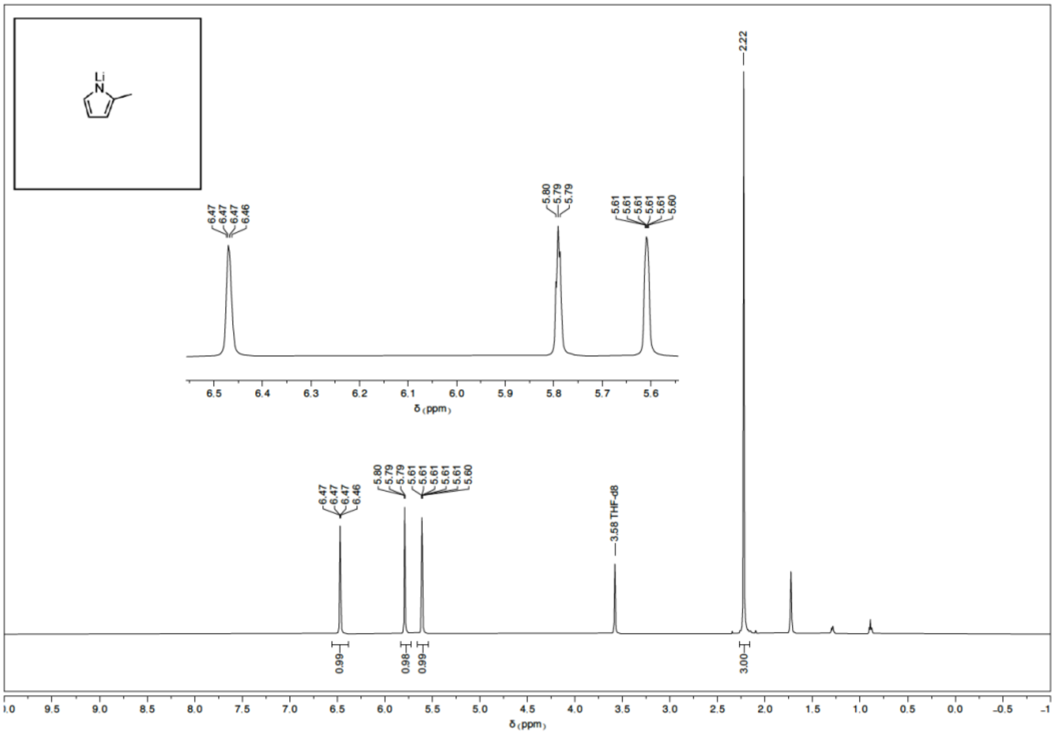

Figure S13. ^{1}H NMR spectrum of lithium 2-methylpyrrolide in THF-d_8 (500 MHz).

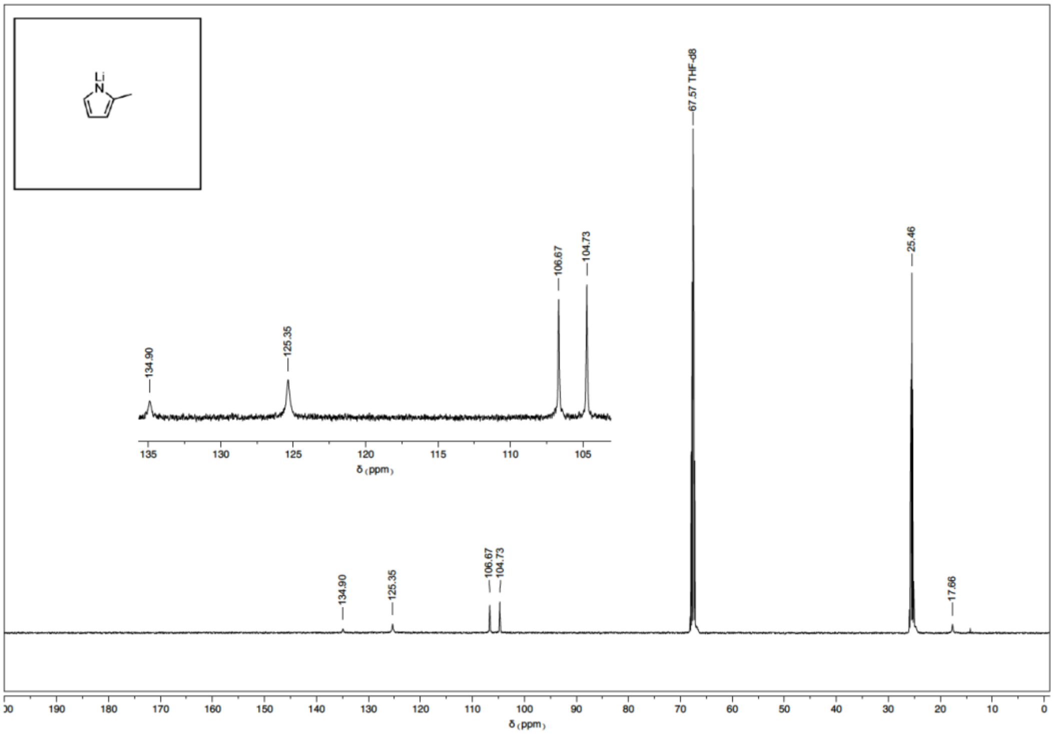

Figure S14. $^{13}C\{^{1}H\}$ NMR spectrum of lithium 2-methylpyrrolide in THF-d_8 (126 MHz).

XV. NMR Spectra of Phosphines

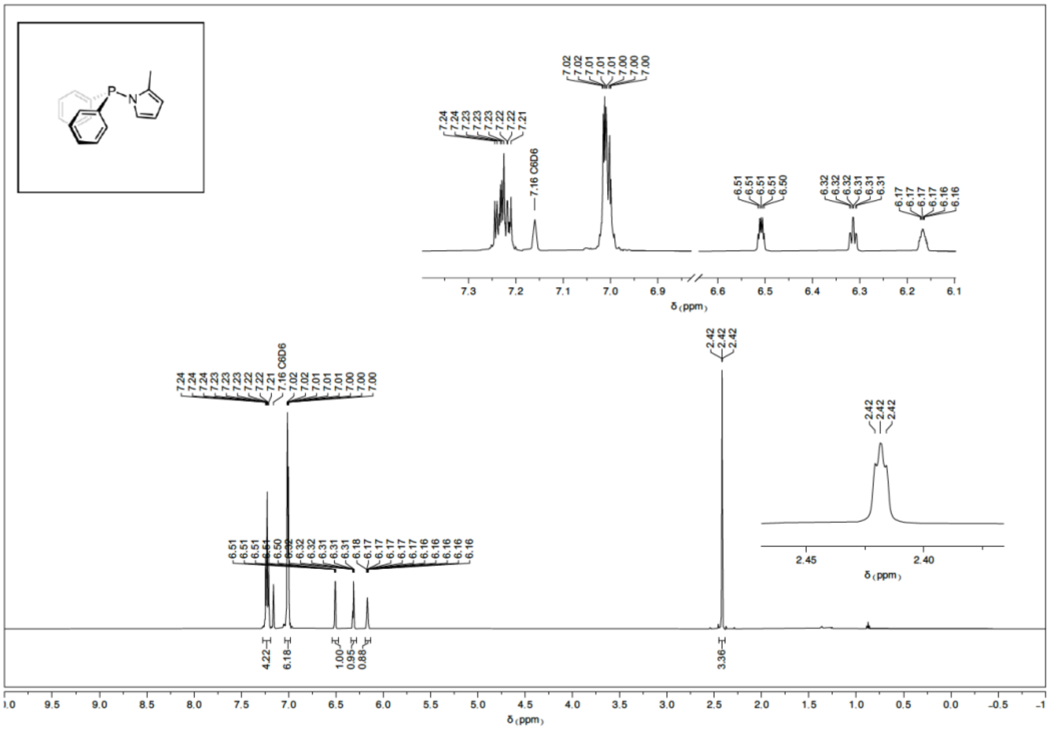

Figure S15. ^{1}H NMR spectrum of 1-(diphenylphosphino)-2-methylpyrrole in C_6D_6 (500 MHz).

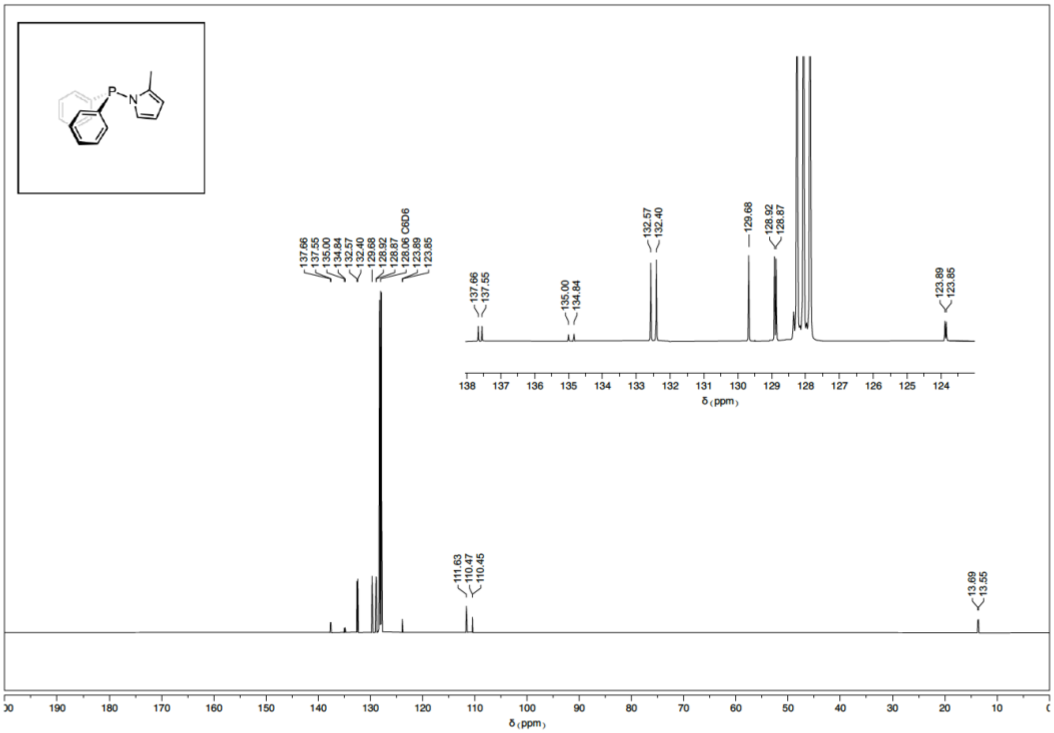

Figure S16. $^{13}C\{^{1}H\}$ NMR spectrum of 1-(diphenylphosphino)-2-methylpyrrole in C_6D_6 (126 MHz).

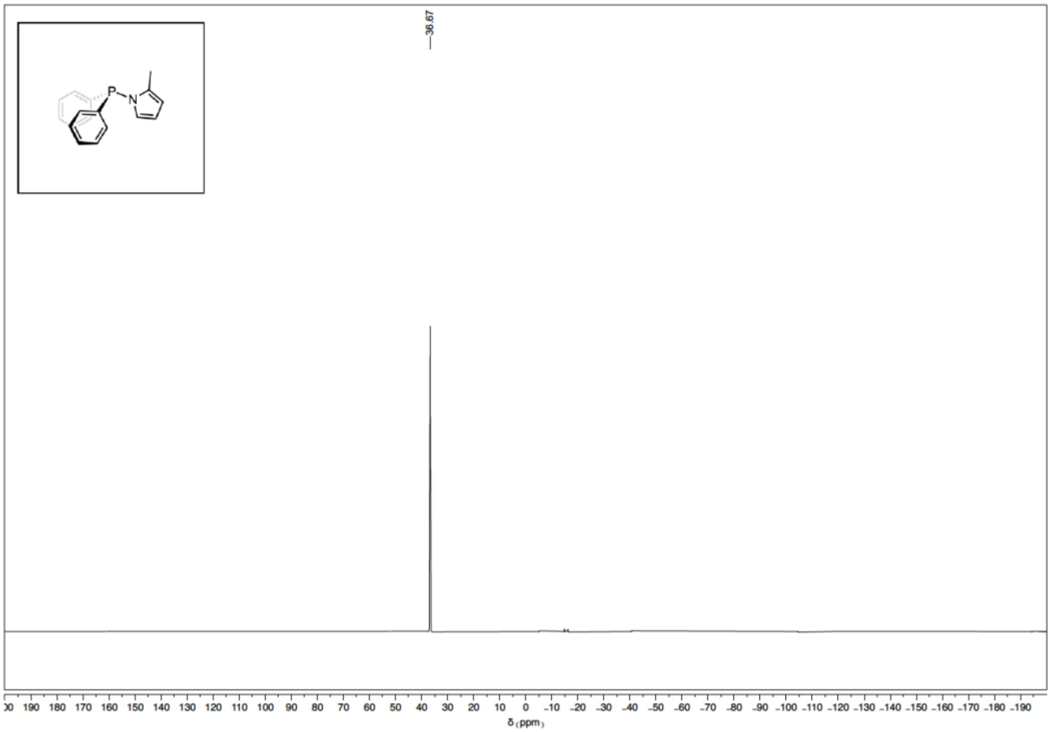

Figure S17. ^{31}P {^{1}H} NMR spectrum of 1-(diphenylphosphino)-2-methylpyrrole in C_6D_6 (202MHz).

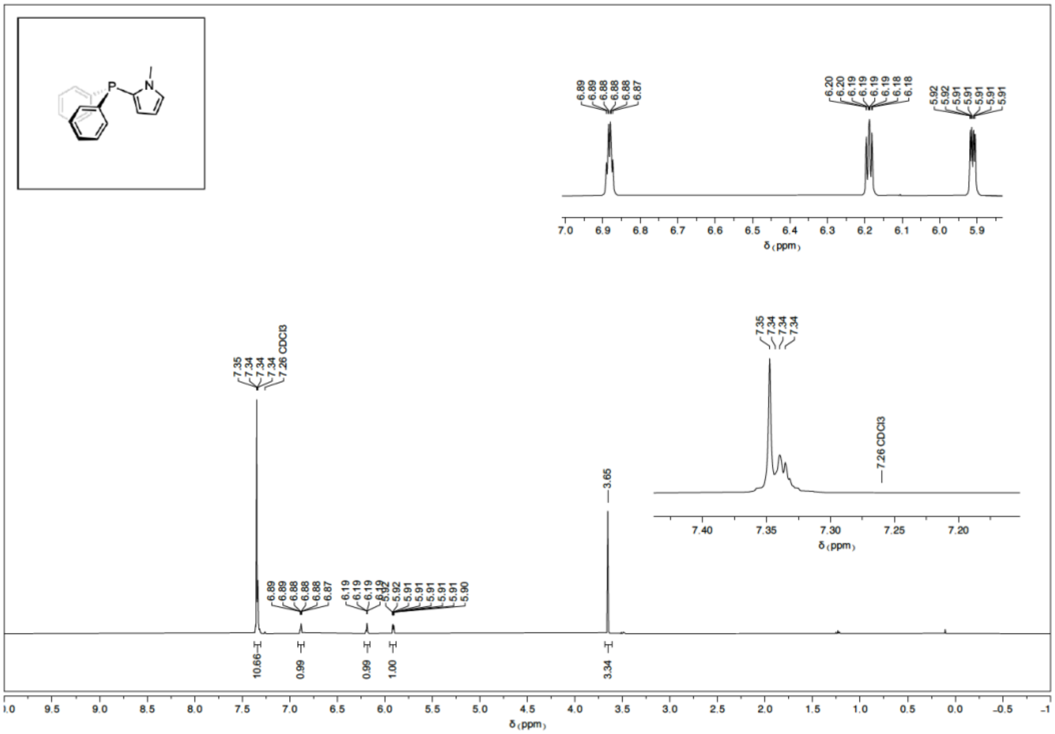

Figure S18. ^{1}H NMR spectrum of 2-(diphenylphosphino)-1-methylpyrrole in $CDCl_3$ (400 MHz).

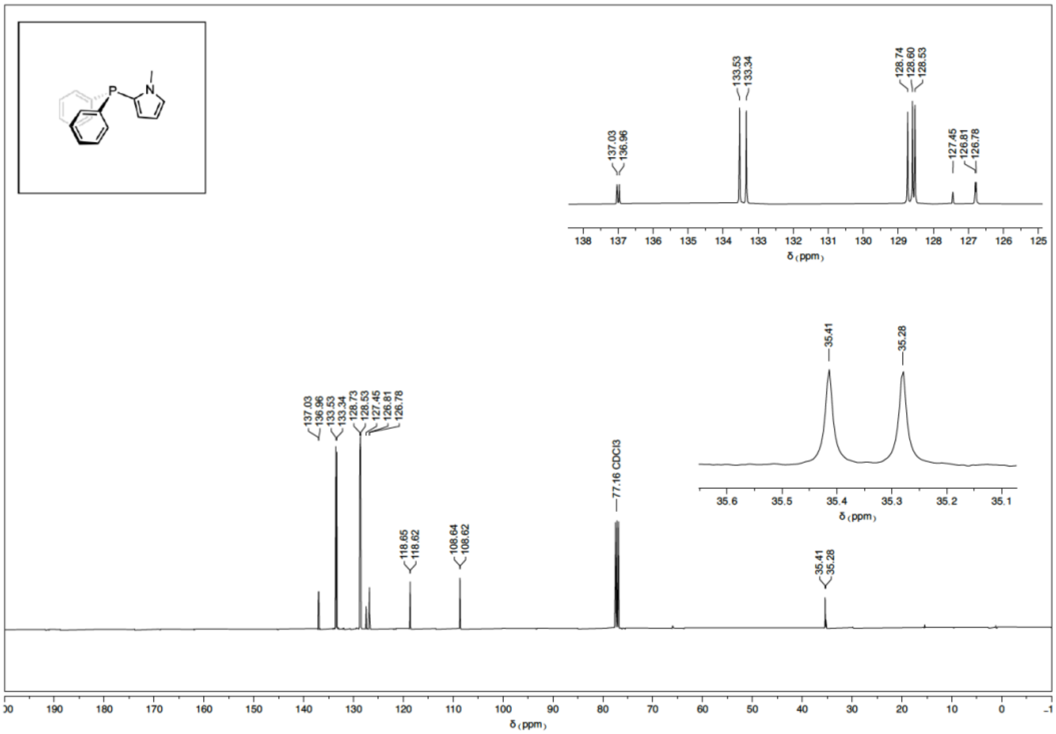

Figure S19. $^{13}C\{^{1}H\}$ NMR spectrum of 2-(diphenylphosphino)-1-methylpyrrole in $CDCl_3$ (126 MHz).

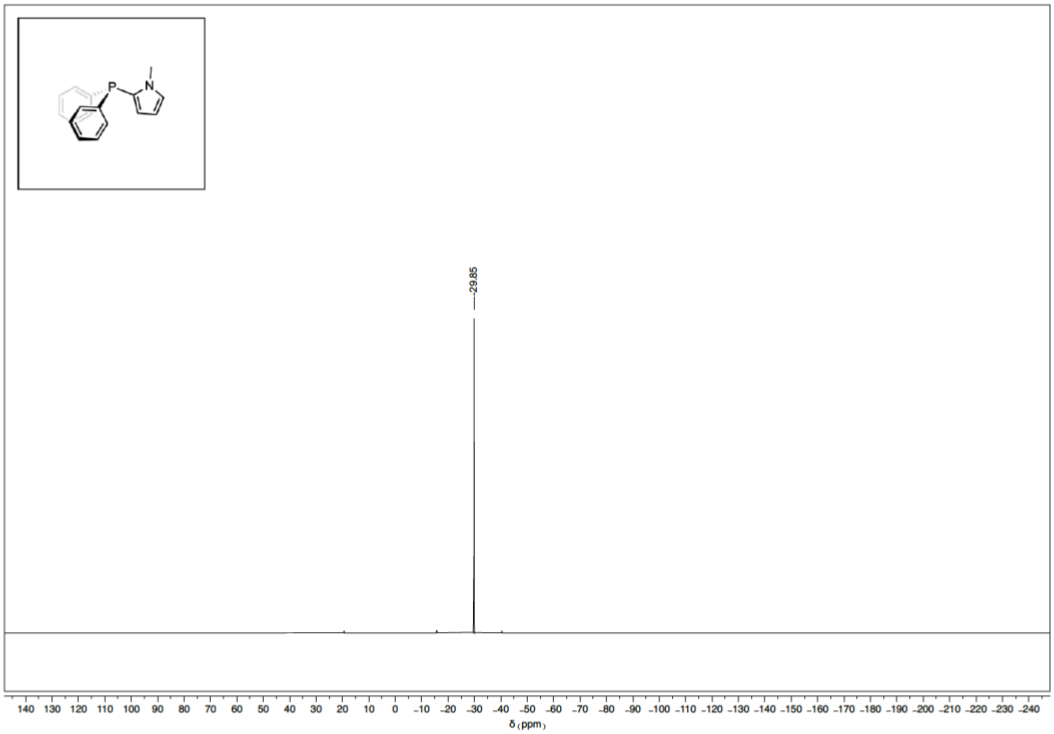

Figure S20. $^{31}P\{^{1}H\}$ NMR spectrum of 2-(diphenylphosphino)-1-methylpyrrole in $CDCl_3$ (202 MHz).

XVI. NMR Spectra of Phosphine Selenides

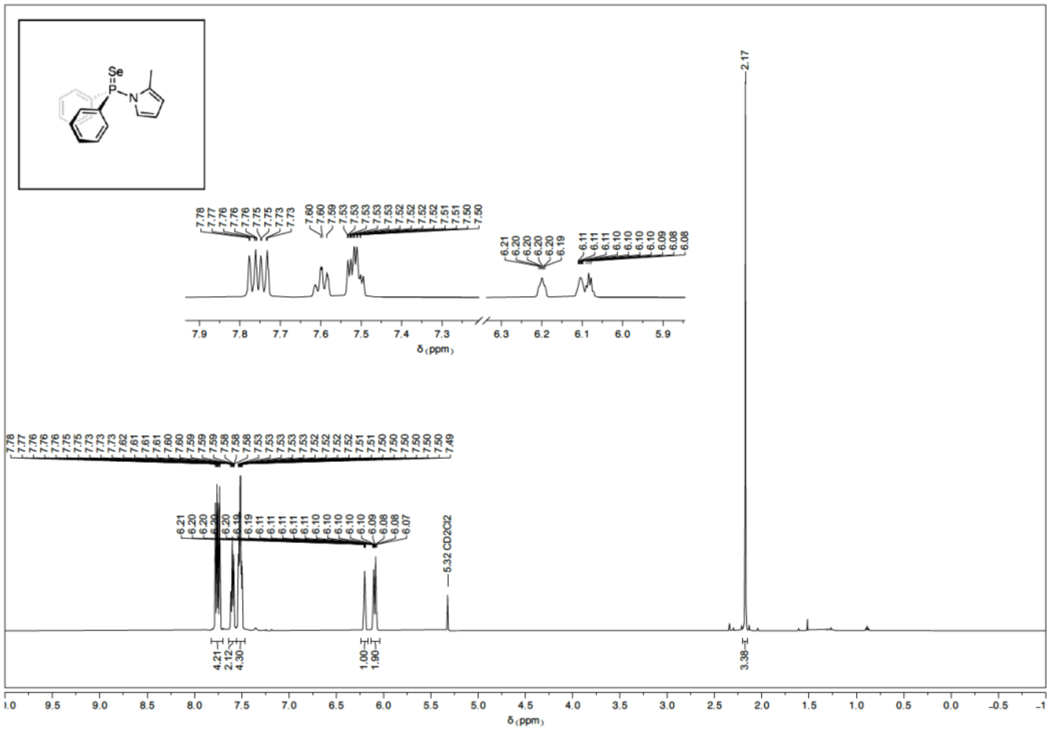

Figure S21. ^{1}H NMR spectrum **L1^{Se}** in CD_2Cl_2 (500 MHz)

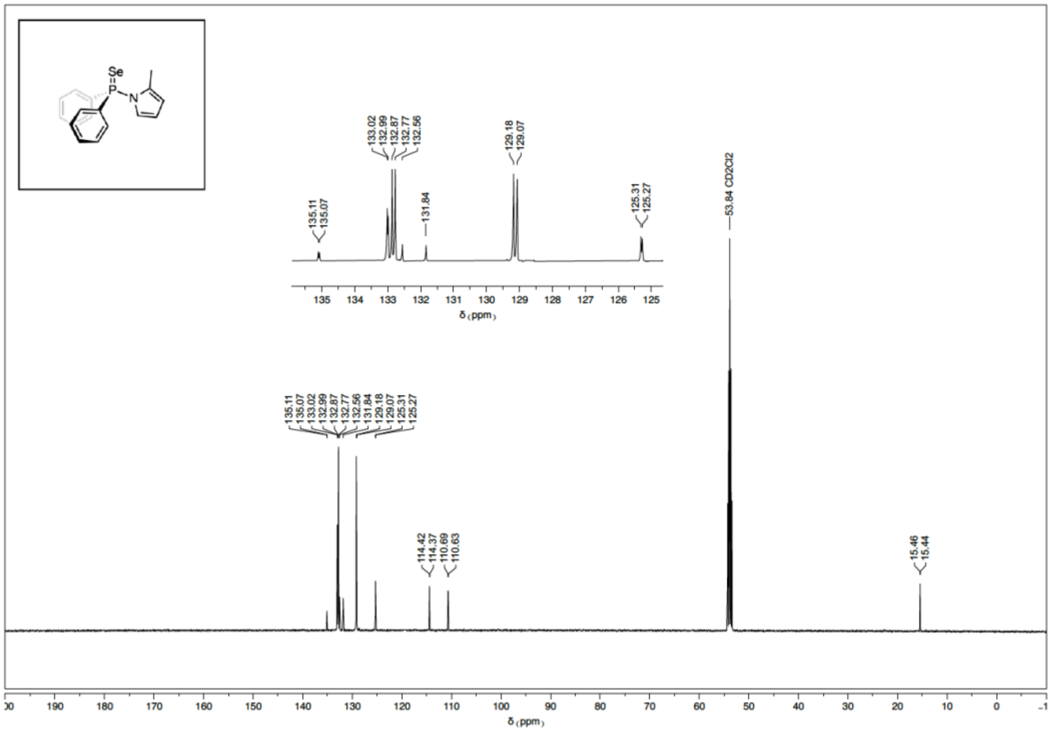

Figure S22. $^{13}C\{^{1}H\}$ NMR spectrum **L1^{Se}** in CD_2Cl_2 (126 MHz)

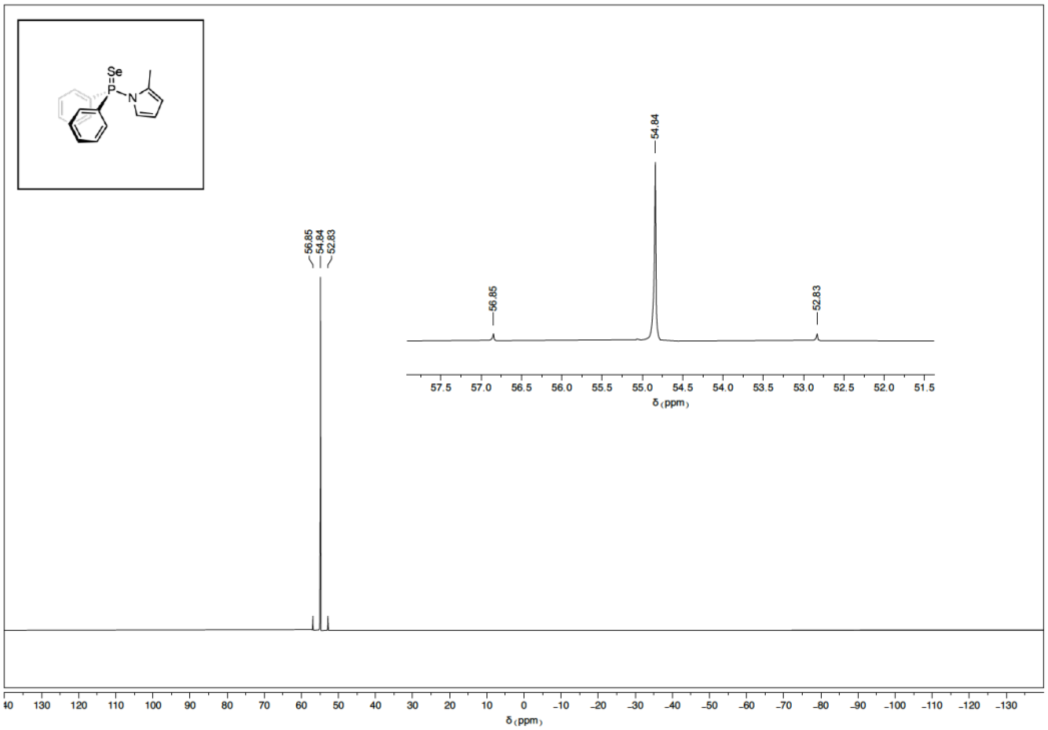

Figure S23. $^{31}P\{^{1}H\}$ NMR spectrum **L1^{Se}** in CD_2Cl_2 (202 MHz)

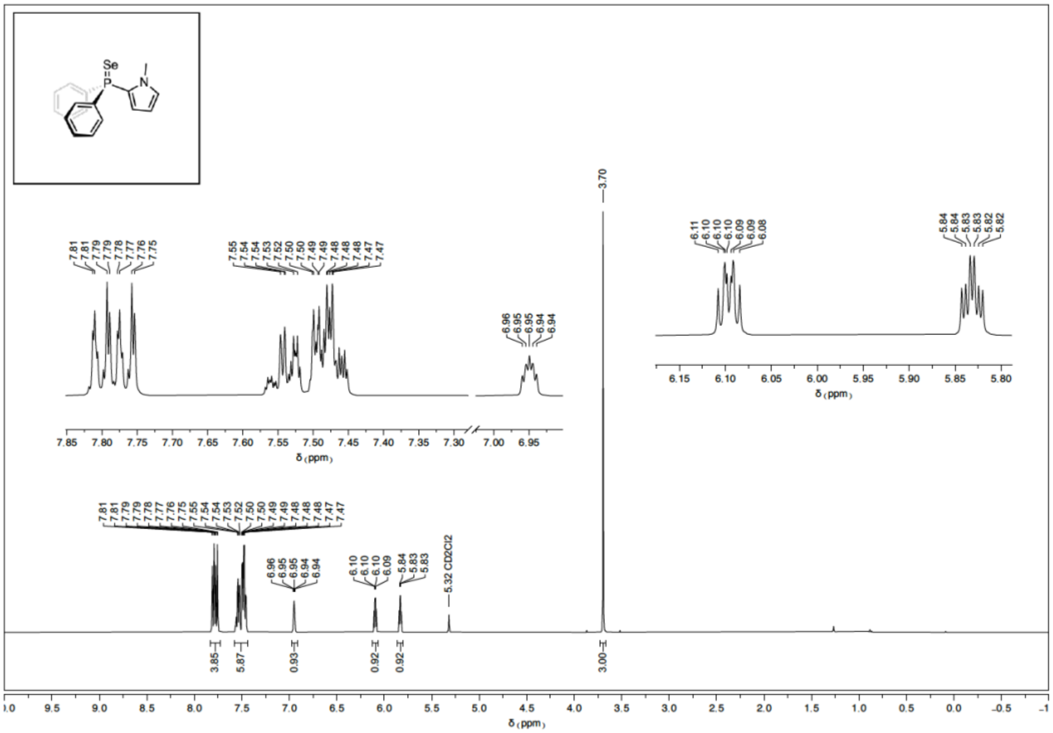

Figure S24. ^{1}H NMR spectrum **L2^{Se}** in CD_2Cl_2 (400 MHz).

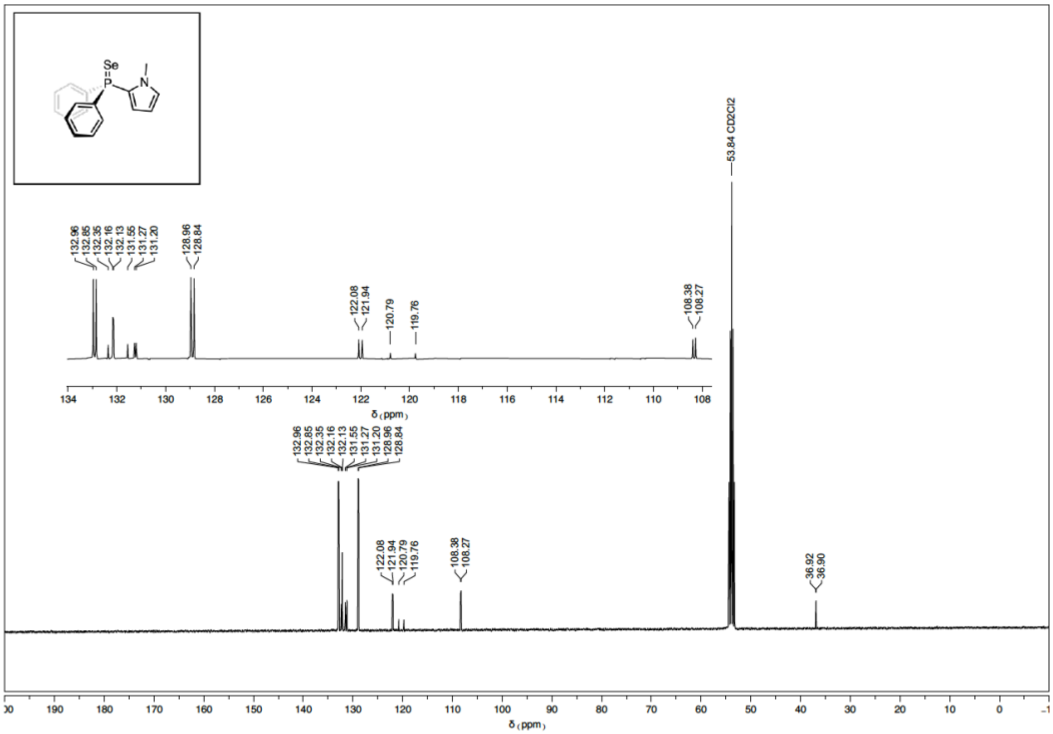

Figure S25. $^{13}C\{^{1}H\}$ NMR spectrum **L2^{Se}** in CD_2Cl_2 (101 MHz).

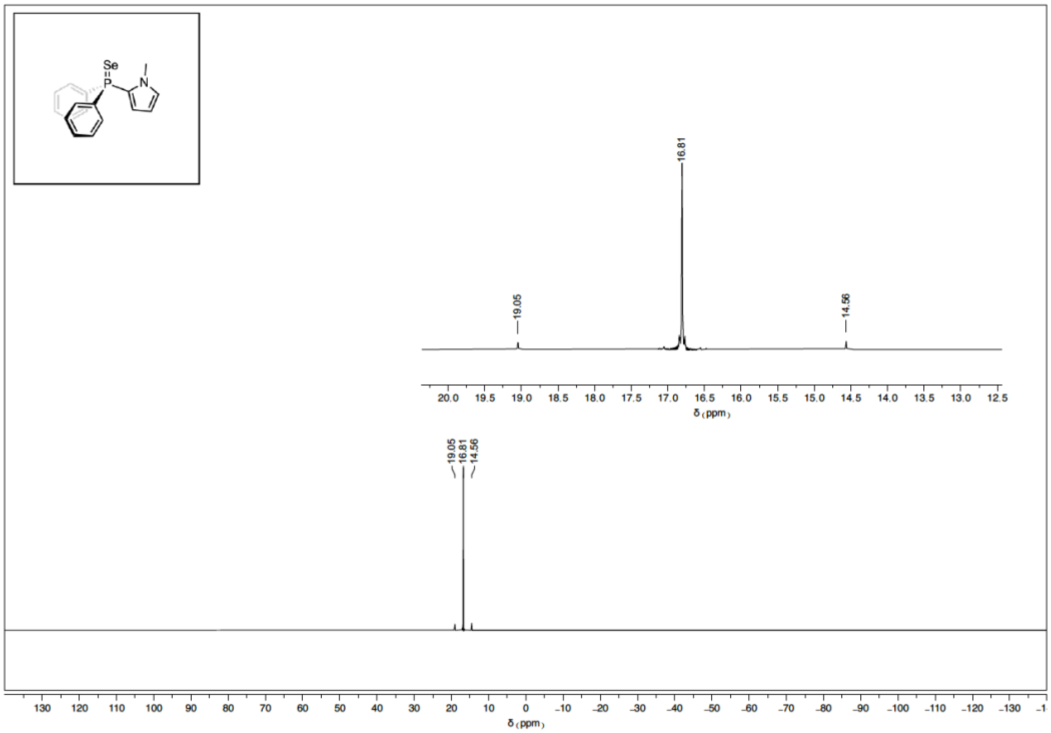

Figure S26. $^{31}P\{^{1}H\}$ NMR spectrum **L2^{Se}** in CD_2Cl_2 (162 MHz).

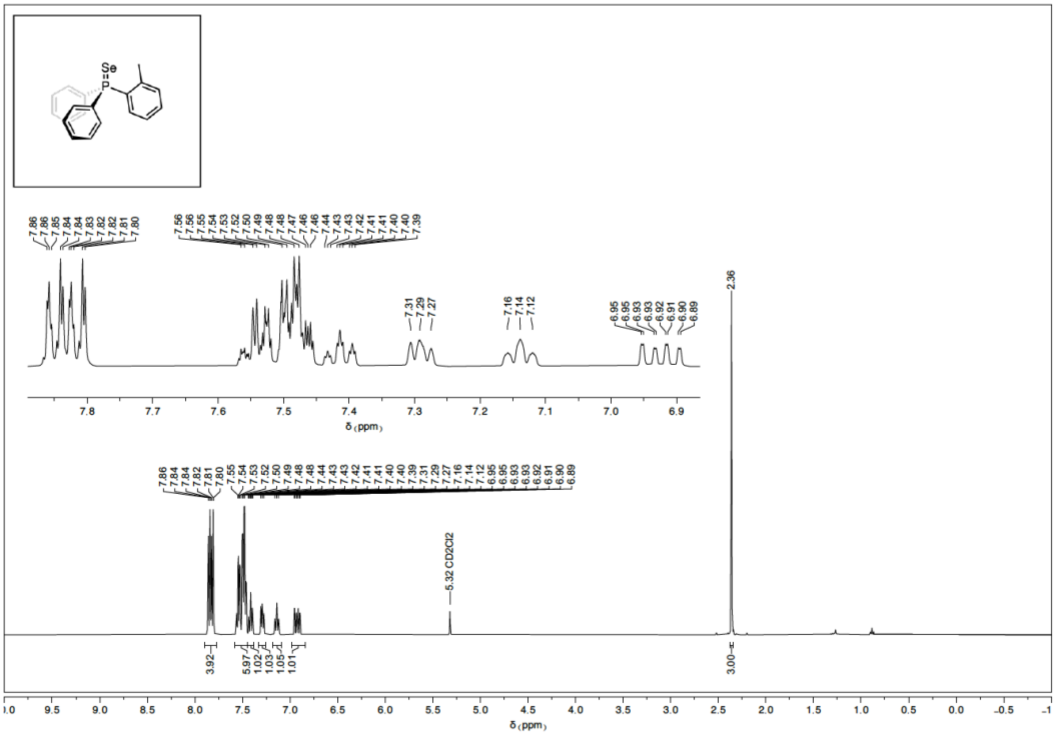

Figure S27. ^{1}H NMR spectrum **L3^{Se}** in CD_2Cl_2 (400 MHz).

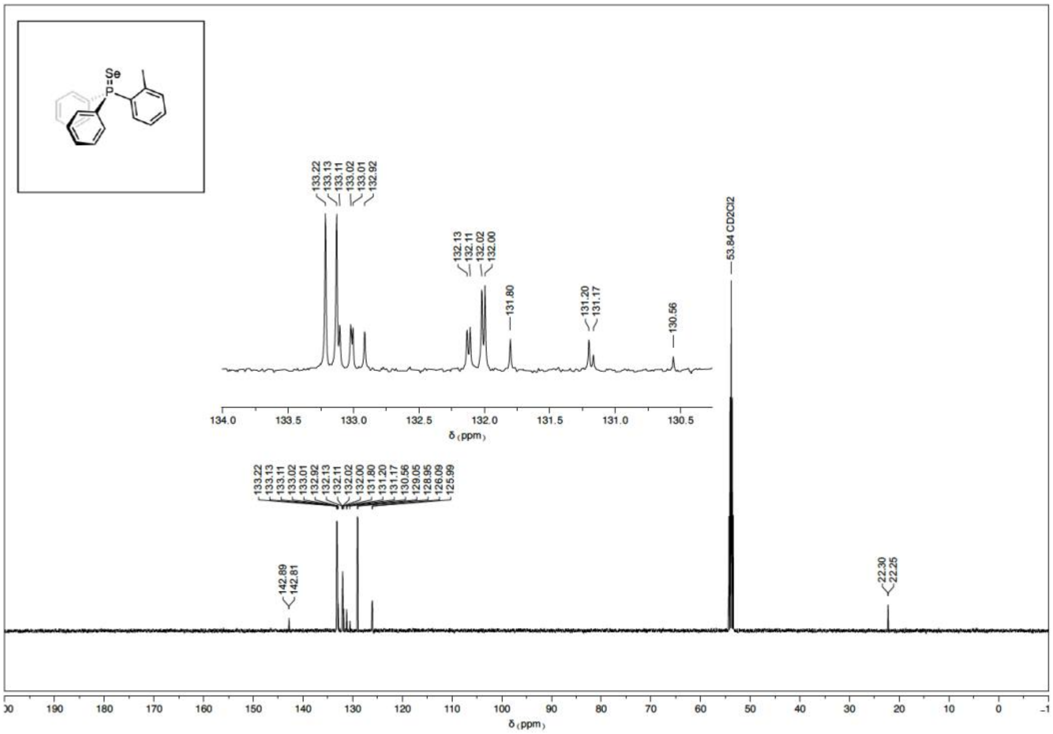

Figure S28. $^{13}C\{^{1}H\}$ NMR spectrum **L3^{Se}** in CD_2Cl_2 (126 MHz).

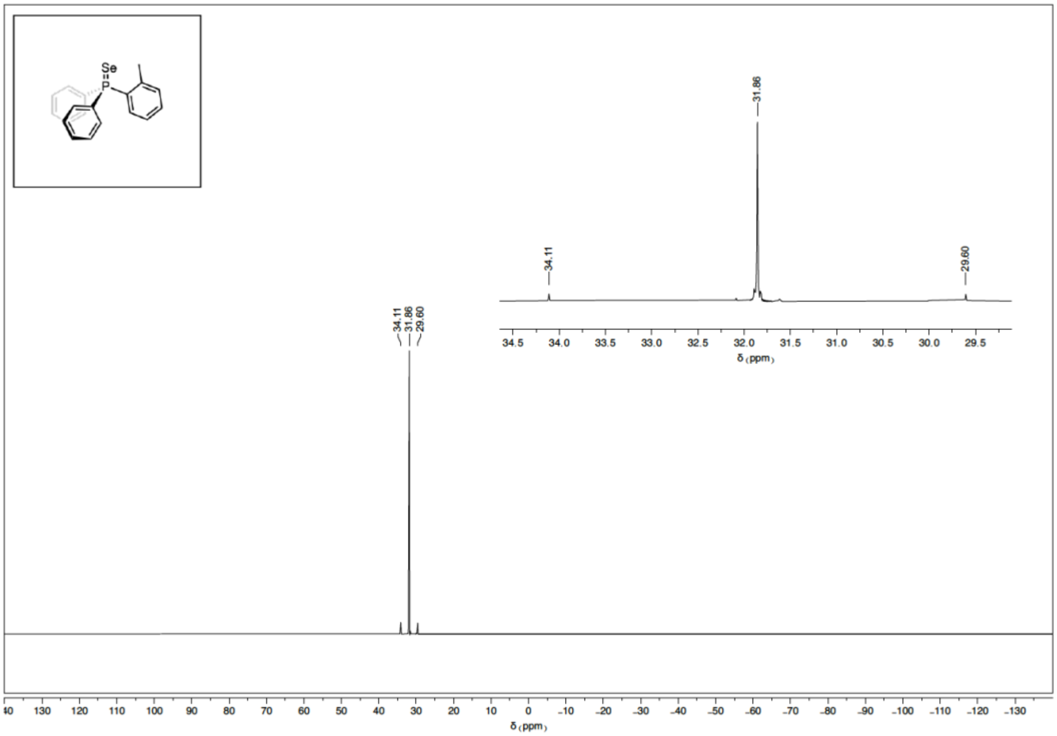

Figure S29. $^{31}P\{^{1}H\}$ NMR spectrum **L3^{Se}** in CD_2Cl_2 (162 MHz).

XVII. NMR Spectra of Complexes (η^6-*p*-cymene)Ru(L)Cl_2

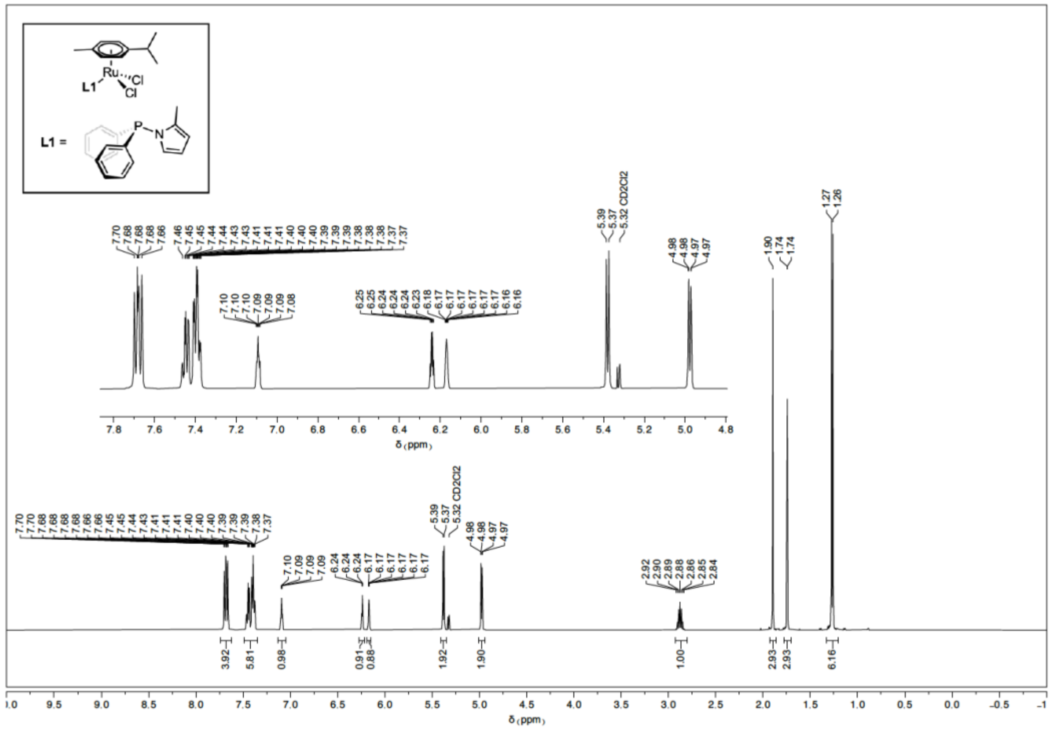

Figure S30. ^1H NMR spectrum of (η^6-p-cymene)Ru(**L1**)Cl_2 in CD_2Cl_2 (500 MHz).

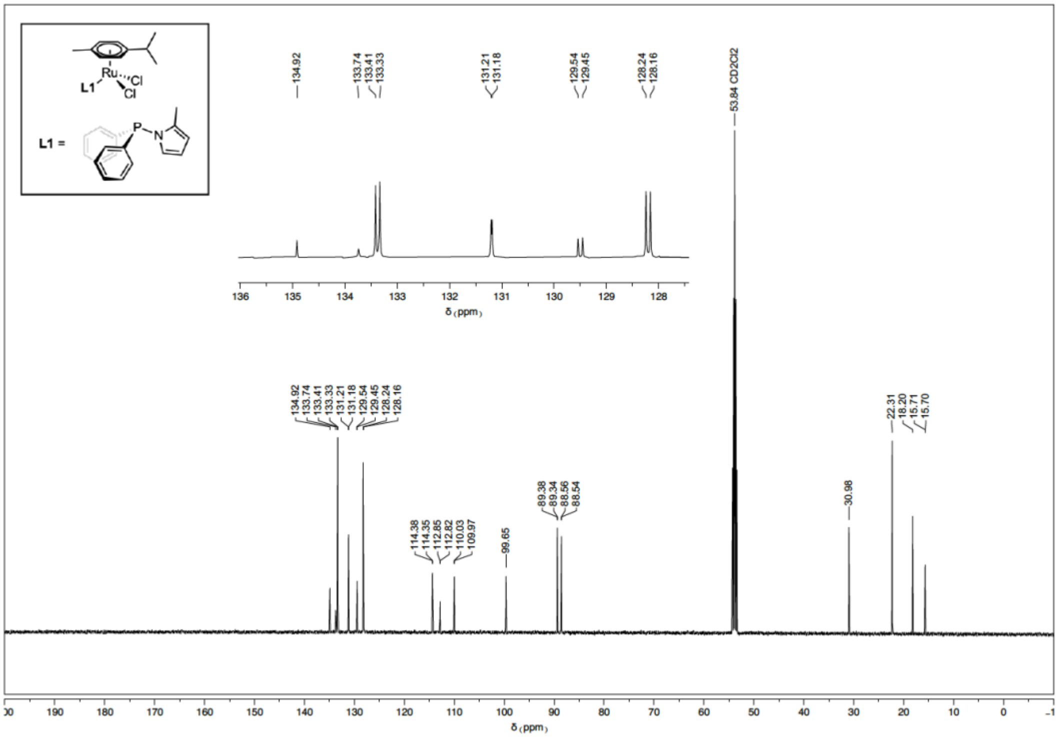

Figure S31. $^{13}C\{^1H\}$ NMR spectrum of (η^6-p-cymene)Ru(**L1**)Cl_2 in CD_2Cl_2 (126 MHz).

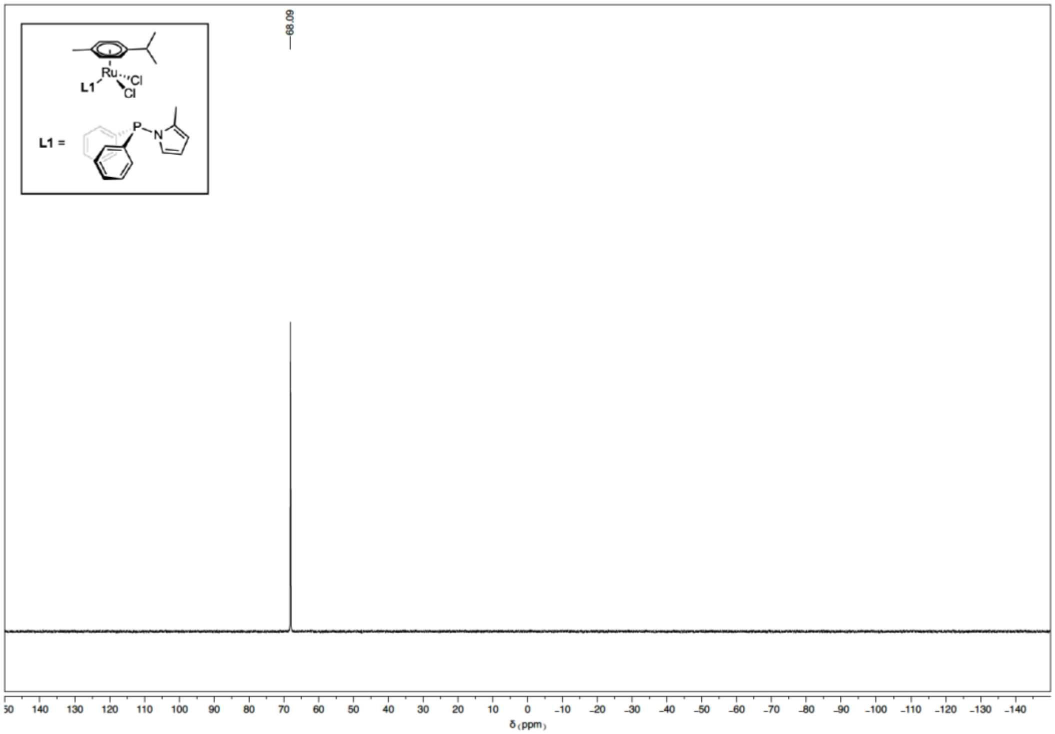

Figure S32. $^{31}P\{^{1}H\}$ NMR spectrum of (η^6-p-cymene)Ru(**L1**)Cl_2 in CD_2Cl_2 (202 MHz).

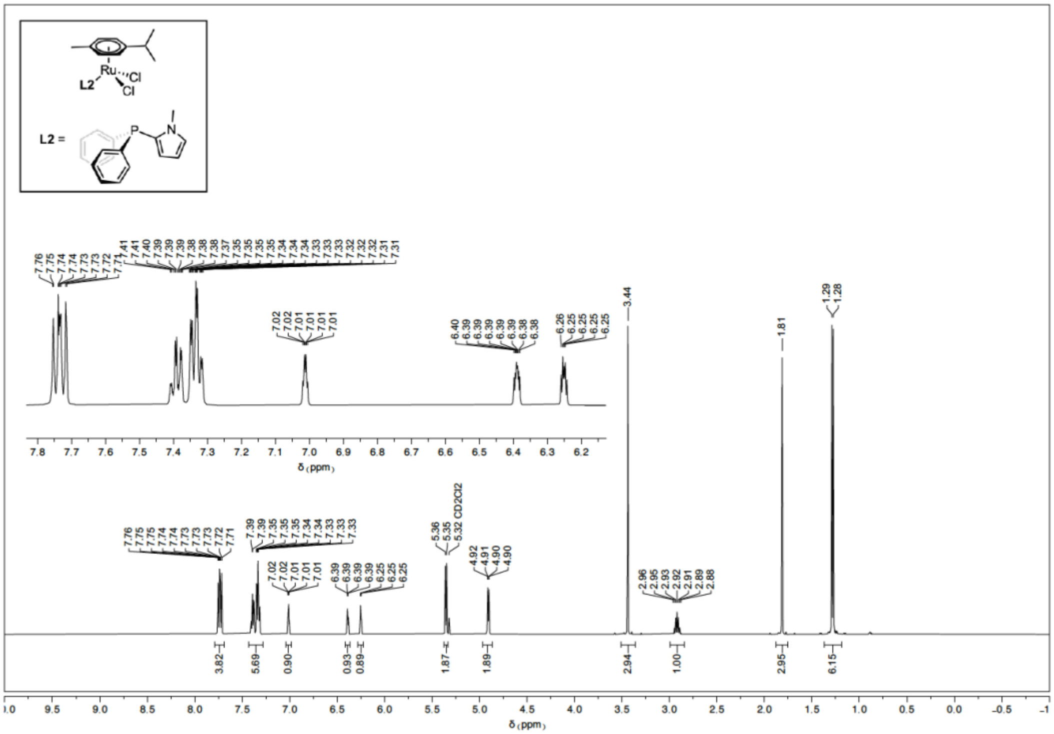

Figure S33. ^{1}H NMR spectrum of (η^6-p-cymene)Ru(**L2**)Cl_2 in CD_2Cl_2 (500 MHz).

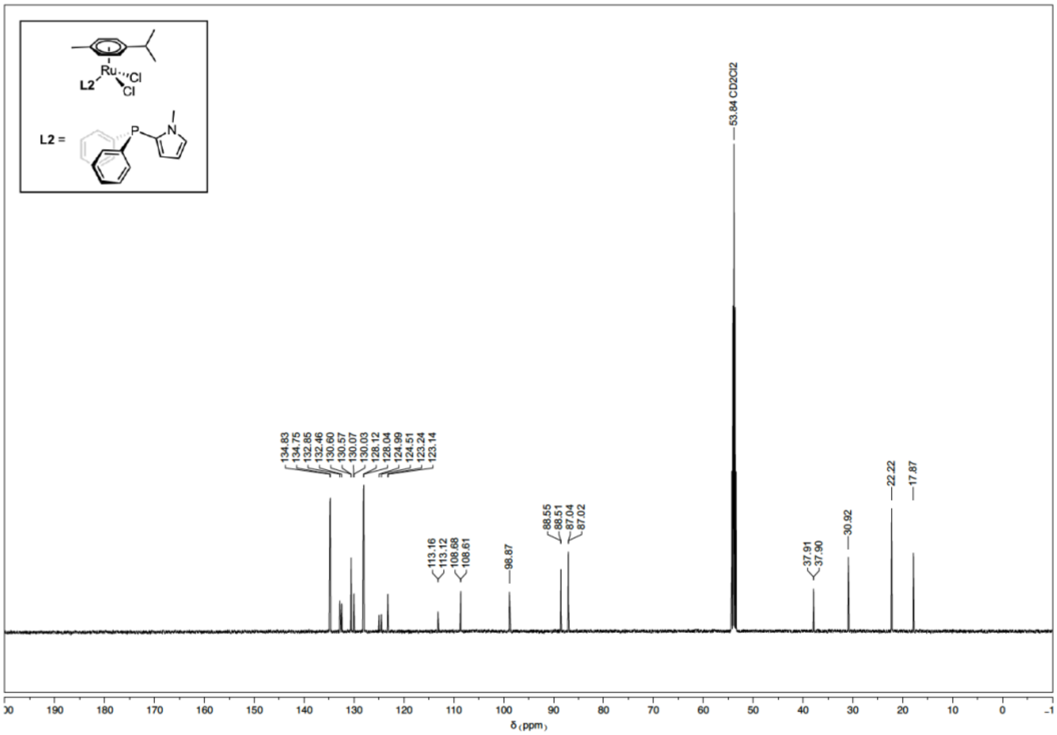

Figure S34. $^{13}C\{^1H\}$ NMR spectrum of (η^6-p-cymene)Ru(**L2**)Cl_2 in CD_2Cl_2 (126 MHz).

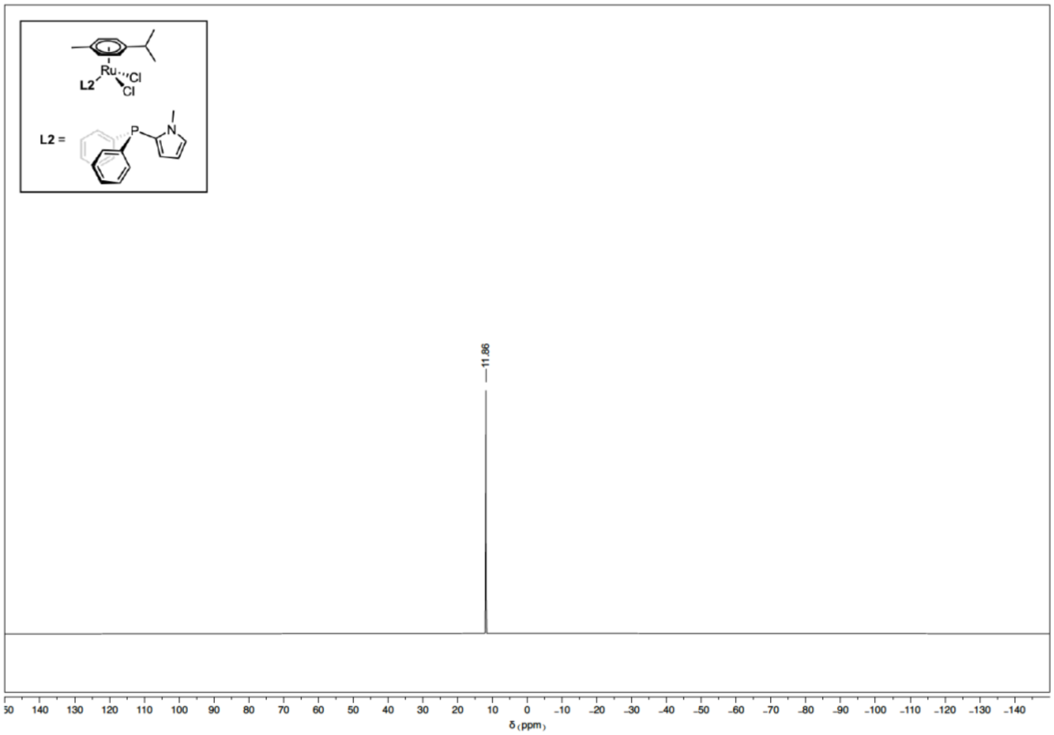

Figure S35. $^{31}P\{^1H\}$ NMR spectrum of (η^6-p-cymene)Ru(**L2**)Cl_2 in CD_2Cl_2 (202 MHz).

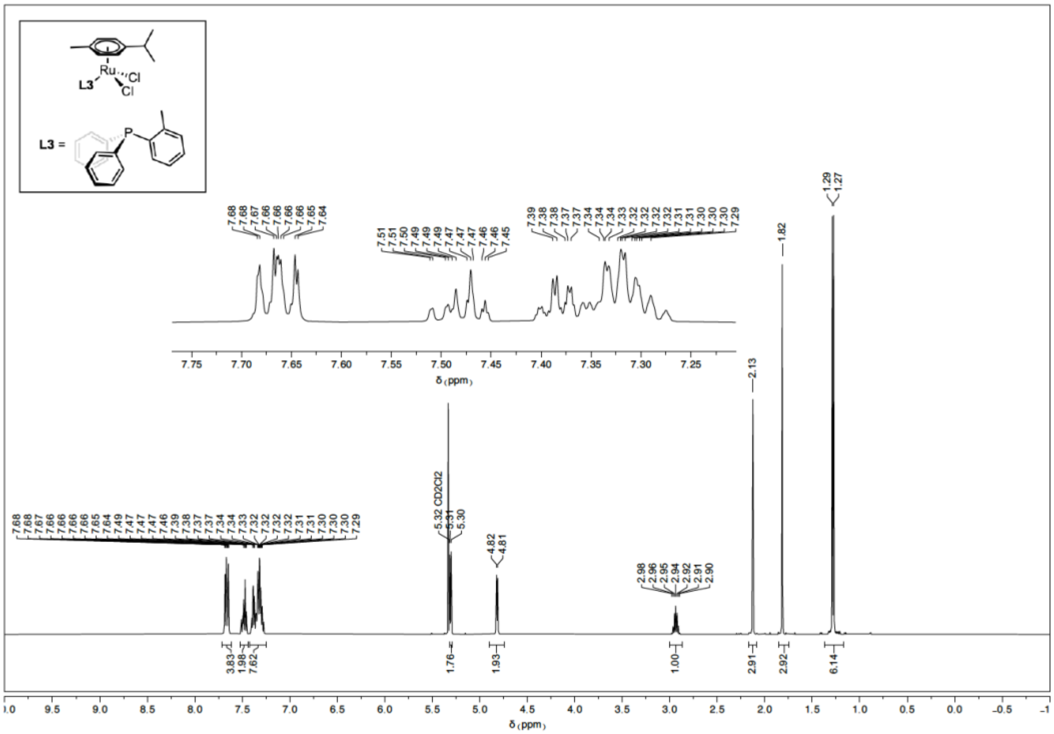

Figure S36. ^{1}H NMR spectrum of (η^{6}-p-cymene)Ru(**L3**)Cl_2 in CD_2Cl_2 (500 MHz).

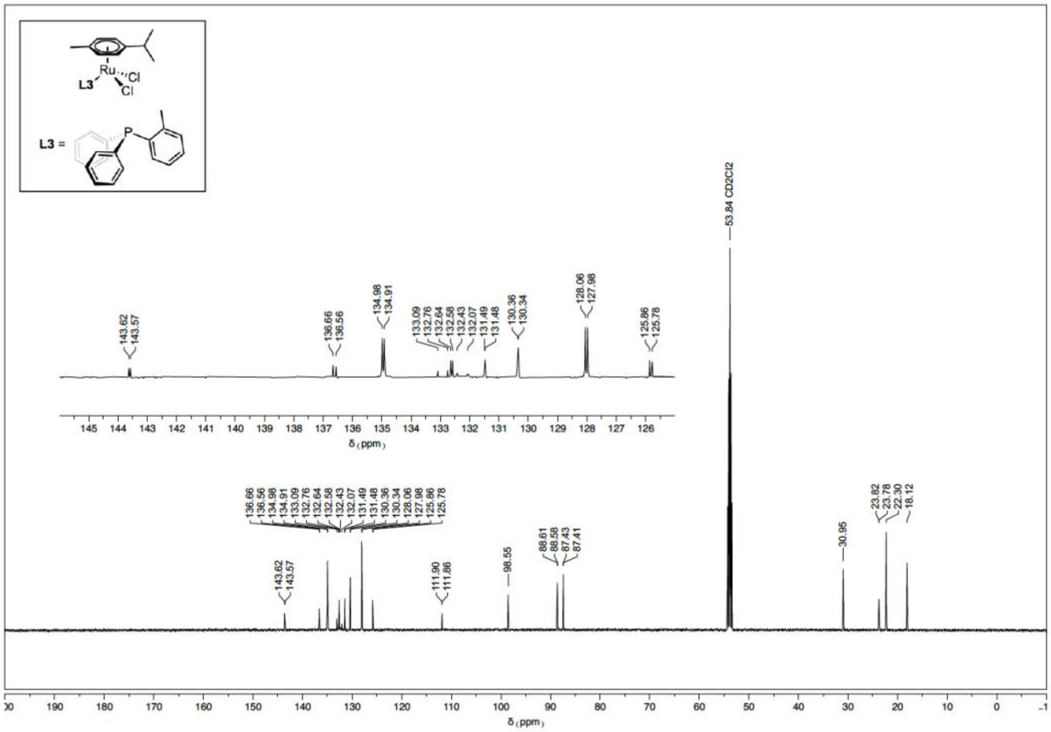

Figure S37. $^{13}C\{^{1}H\}$ NMR spectrum of (η^{6}-p-cymene)Ru(**L3**)Cl_2 in CD_2Cl_2 (126 MHz).

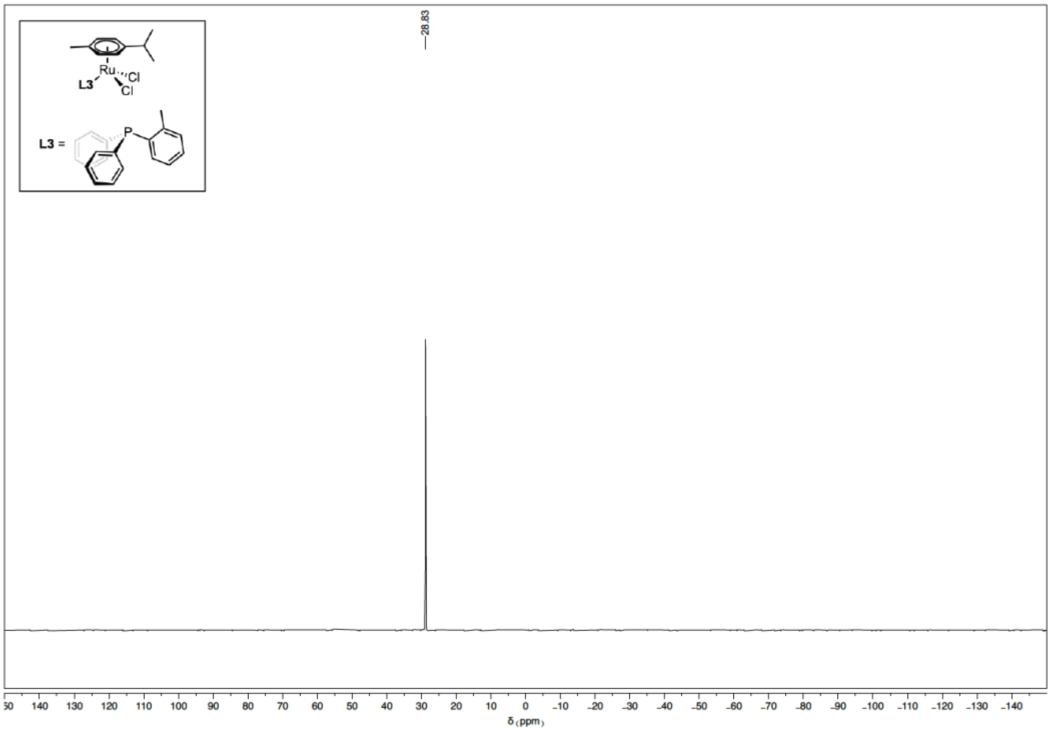

Figure S38. $^{31}P\{^{1}H\}$ NMR spectrum of (η^6-p-cymene)Ru(**L3**)Cl_2 in CD_2Cl_2 (202 MHz).

XVIII. NMR Spectra of Complexes *trans*-(L)$_2$Rh(CO)Cl

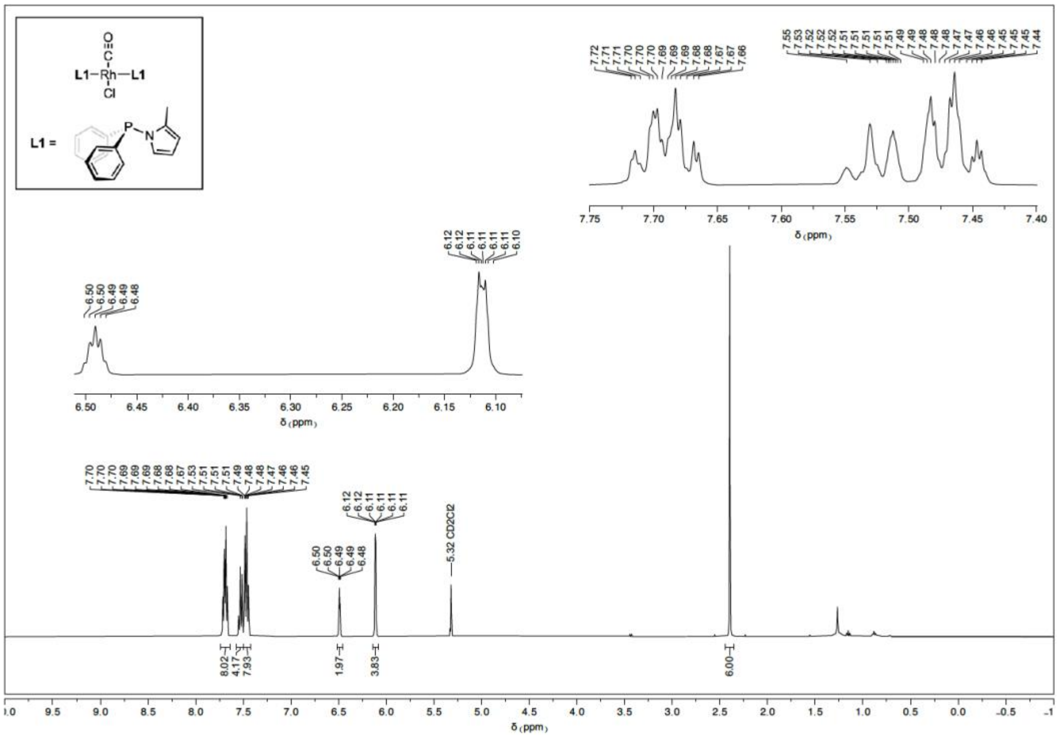

Figure S39. ^{1}H NMR spectrum of *trans*-(**L1**)$_2$Rh(CO)Cl in CD_2Cl_2 (400 MHz).

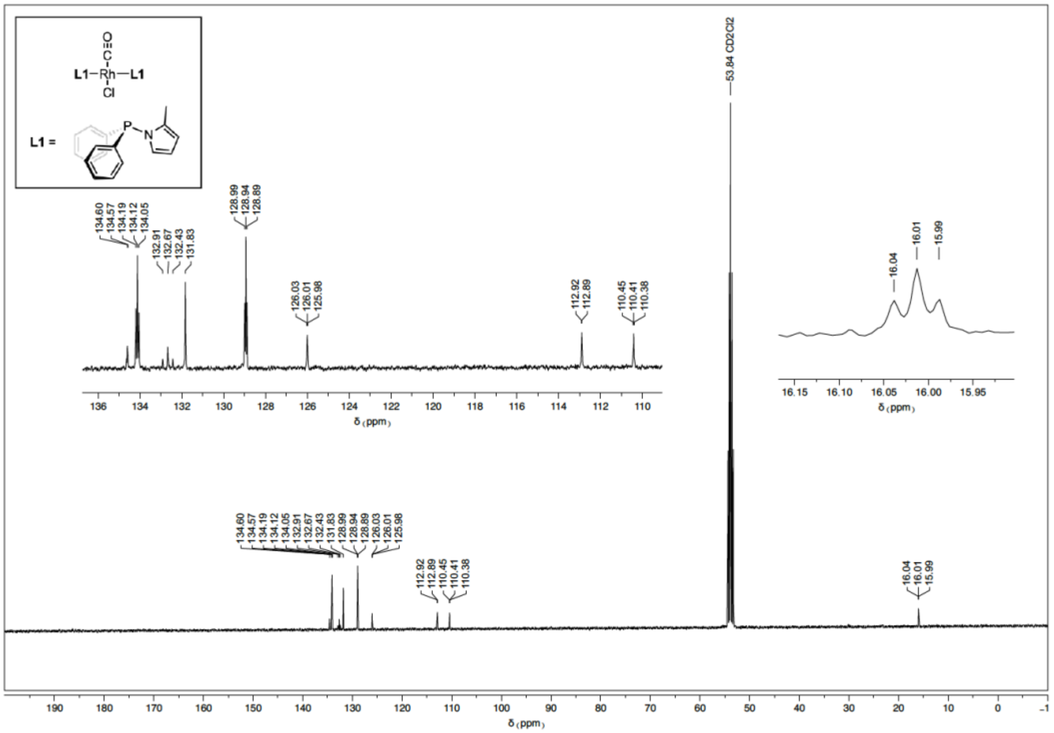

Figure S40. ^{13}C{^{1}H} NMR spectrum of *trans*-(**L1**)$_2$Rh(CO)Cl in CD_2Cl_2 (101 MHz).

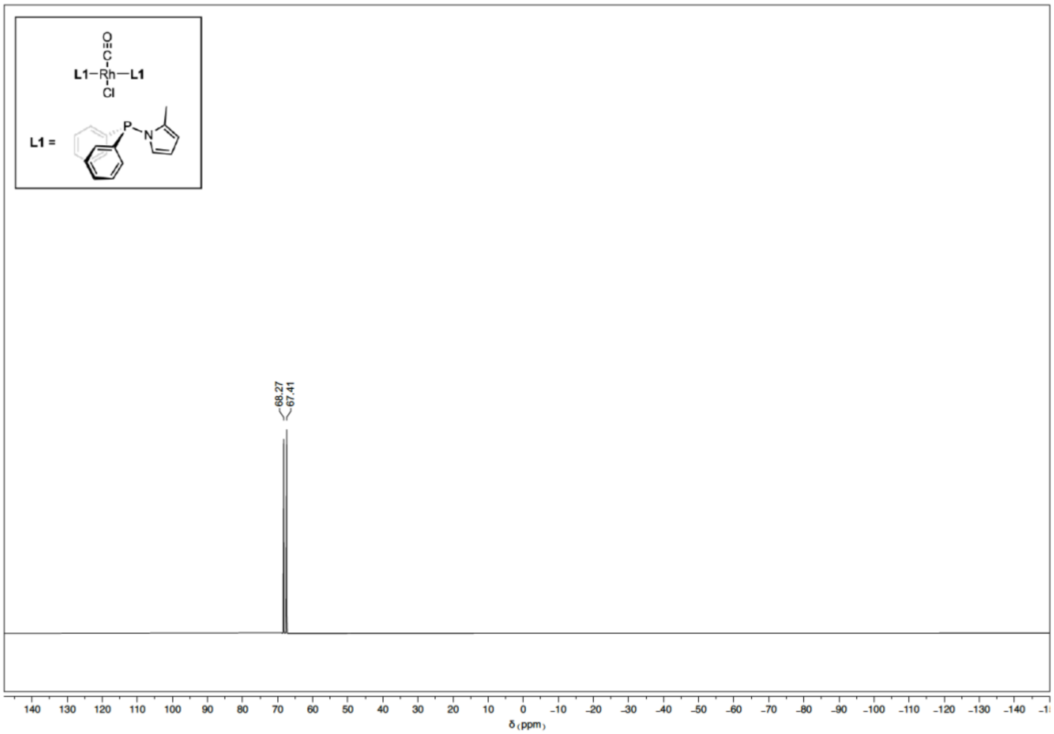

Figure S41. $^{31}P\{^1H\}$ NMR spectrum of *trans*-(**L1**)$_2$Rh(CO)Cl in CD_2Cl_2 (162 MHz).

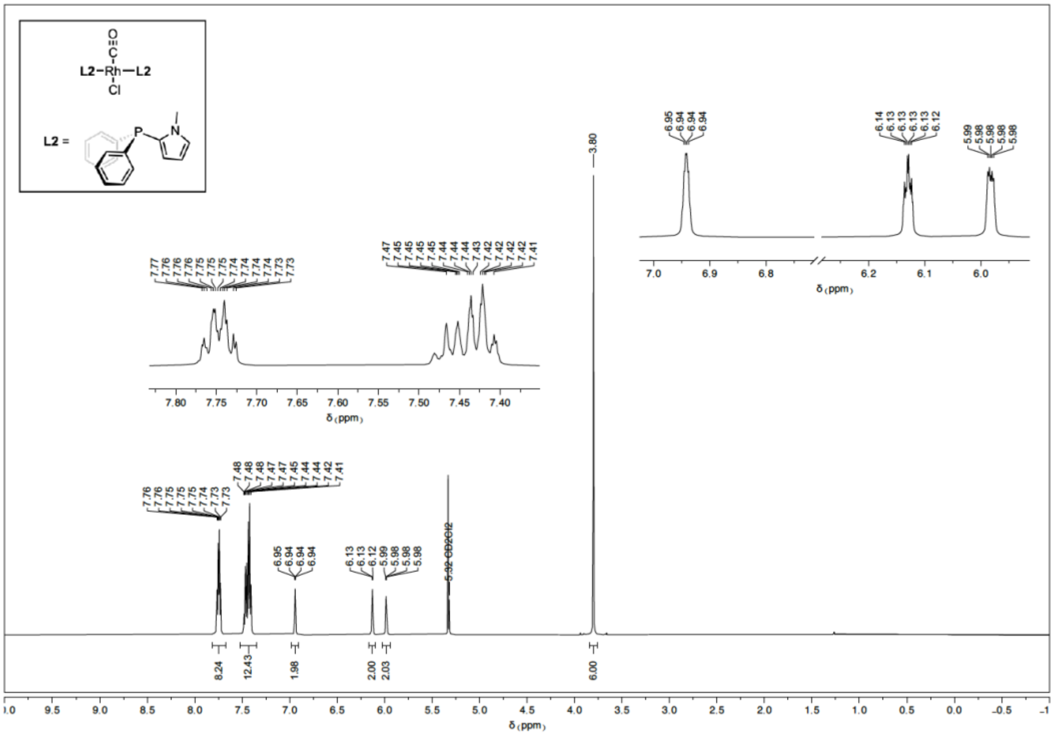

Figure S42. ^{1}H NMR spectrum of *trans*-(**L2**)$_2$Rh(CO)Cl in CD_2Cl_2 (500 MHz).

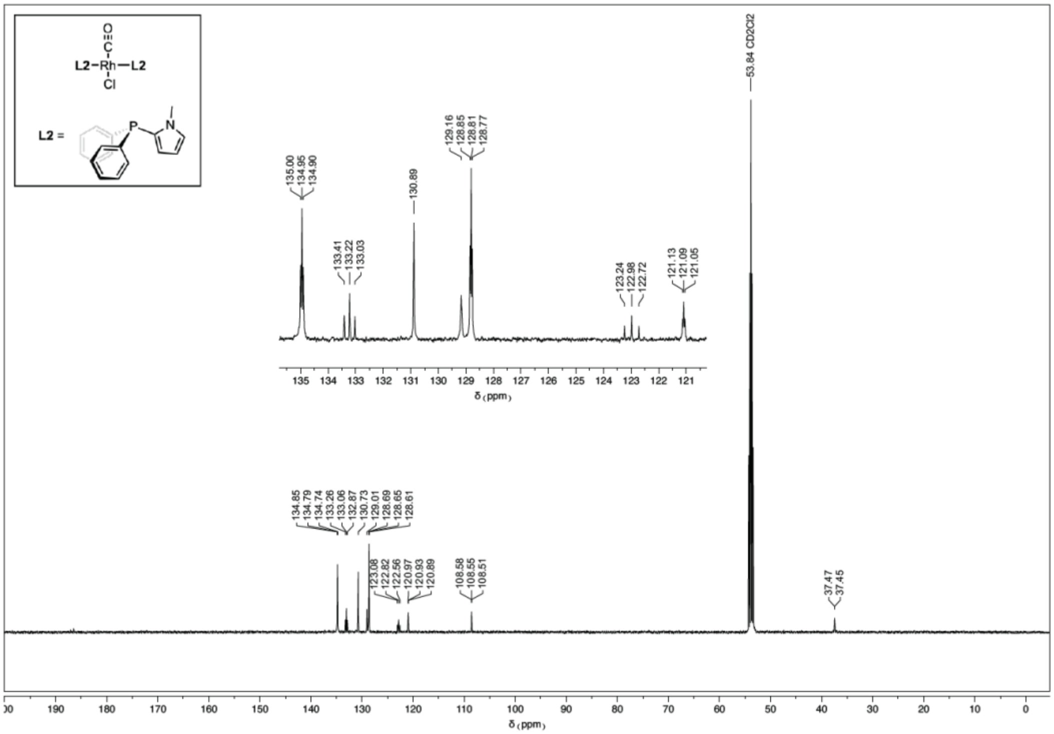

Figure S43. ^{13}C{^{1}H} NMR spectrum of *trans*-(**L2**)$_2$Rh(CO)Cl in CD_2Cl_2 (126 MHz).

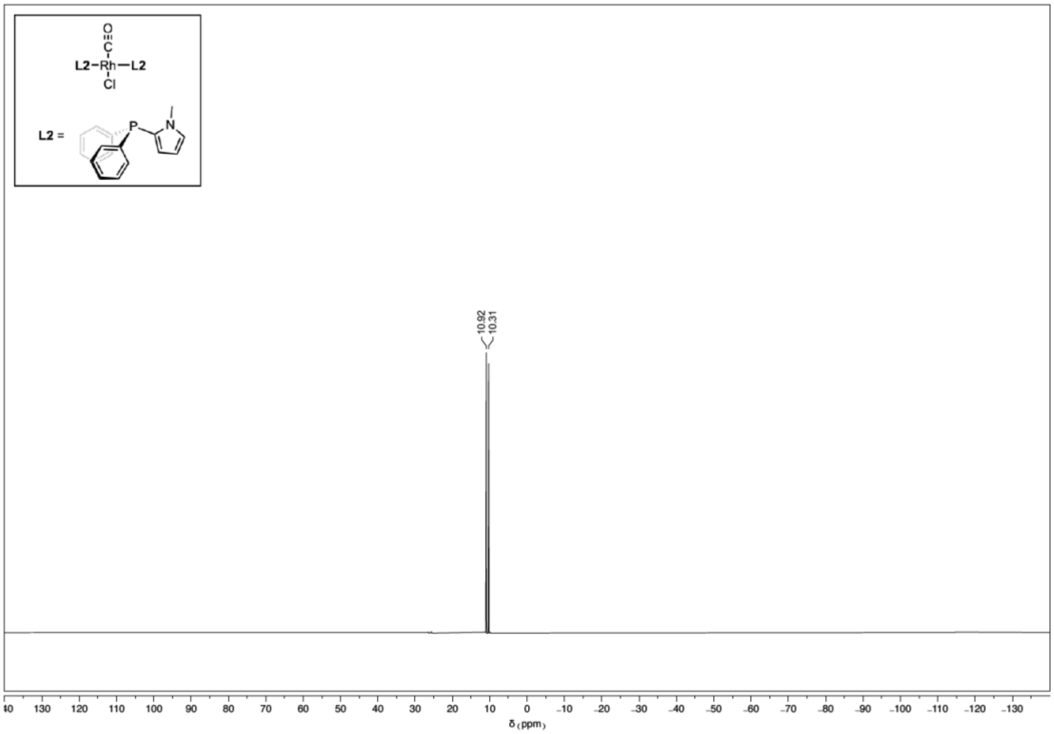

Figure S44. $^{31}P\{^{1}H\}$ NMR spectrum of *trans*-(**L2**)$_2$Rh(CO)Cl in CD_2Cl_2 (202 MHz).

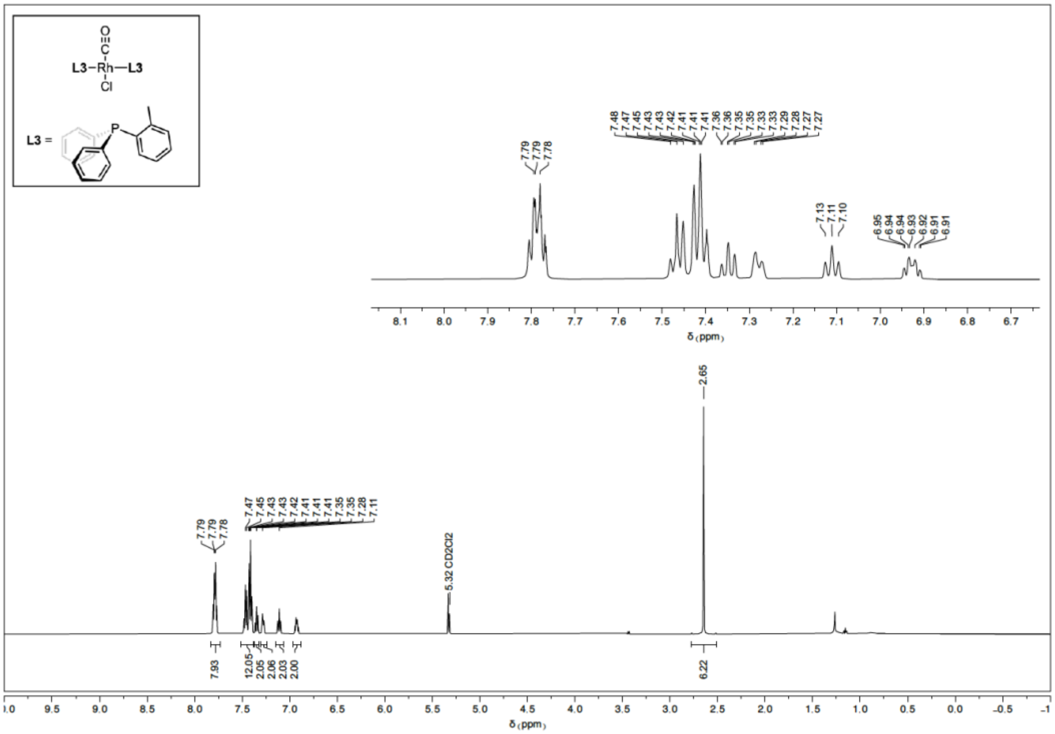

Figure S45. ^{1}H NMR spectrum of *trans*-(**L3**)$_2$Rh(CO)Cl in CD_2Cl_2 (500 MHz).

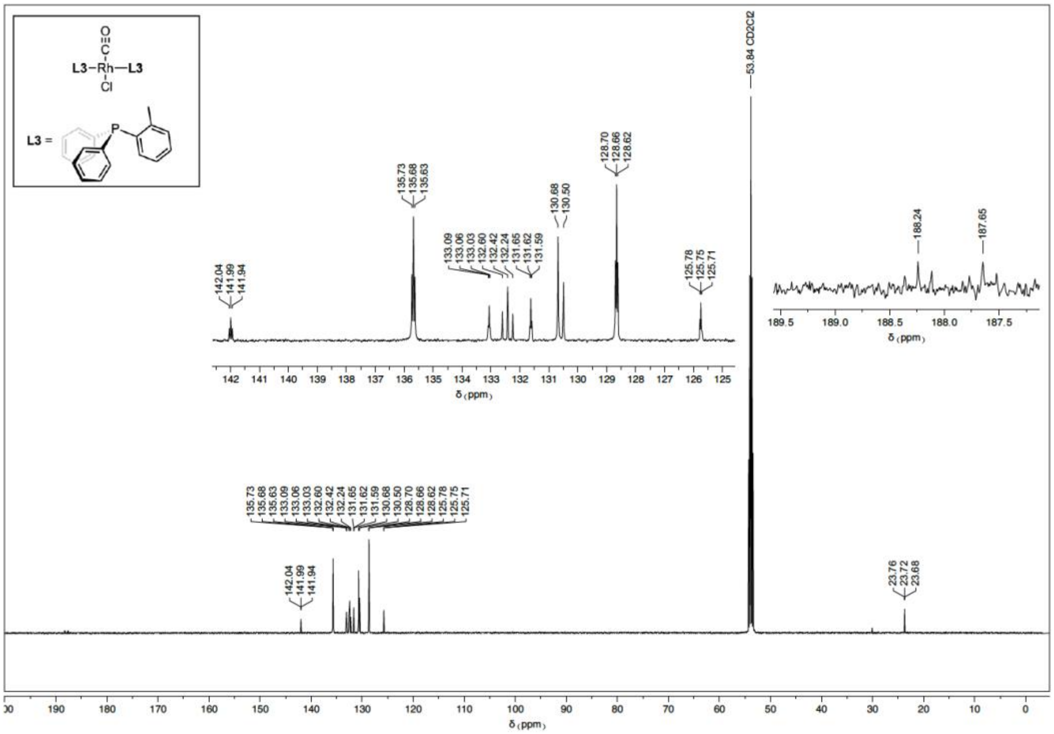

Figure S46. ^{13}C{^{1}H} NMR spectrum of *trans*-(**L3**)$_2$Rh(CO)Cl in CD_2Cl_2 (126 MHz).

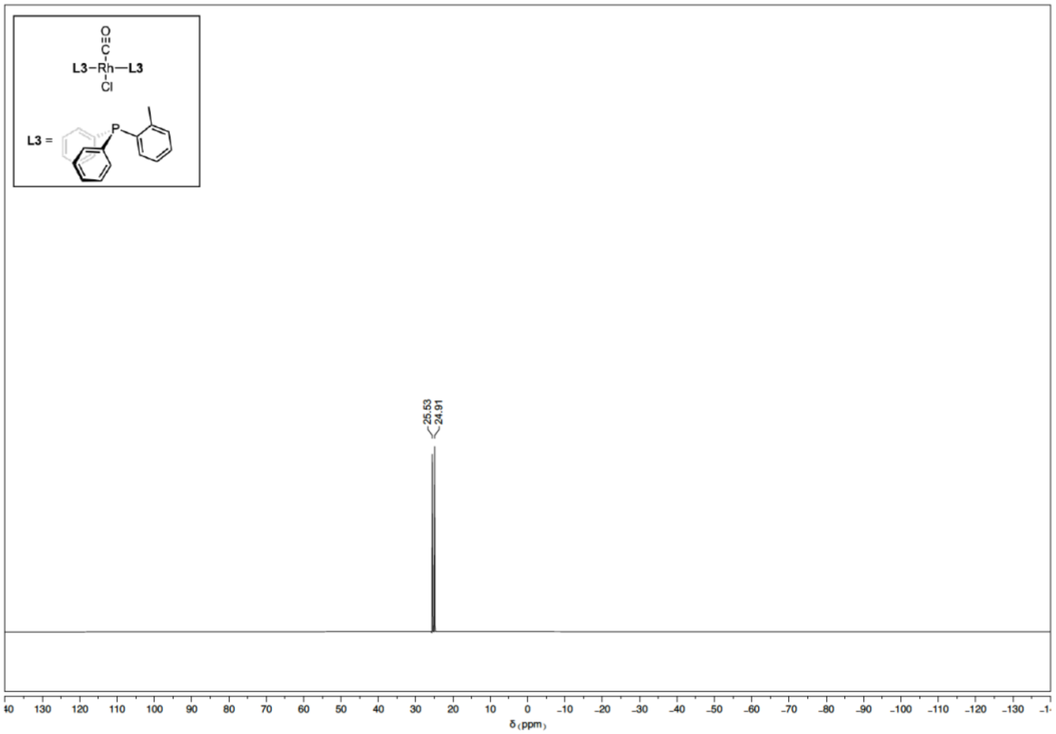

Figure S47. $^{31}P\{^{1}H\}$ NMR spectrum of *trans*-(**L3**)$_2$Rh(CO)Cl in CD_2Cl_2 (202 MHz).

XIX. NMR Spectra of Complexes *cis*-(L)$_2$PtCl$_2$

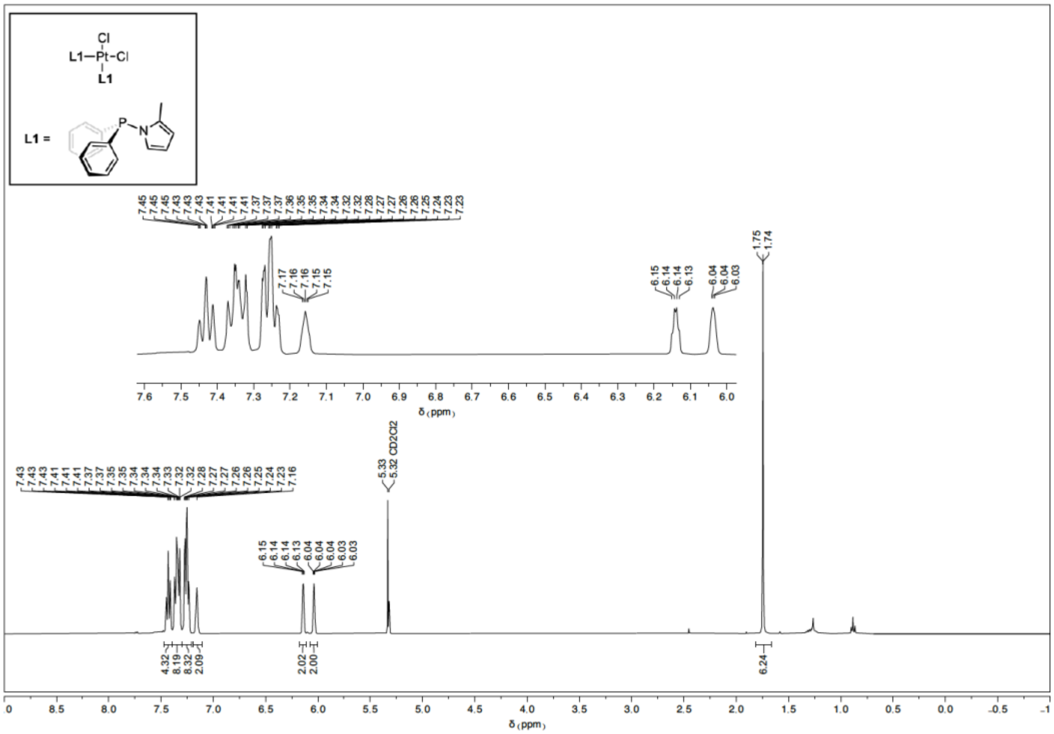

Figure S48. ^{1}H NMR spectrum of *cis*-(**L1**)$_2$PtCl$_2$ in CD_2Cl_2 (400 MHz).

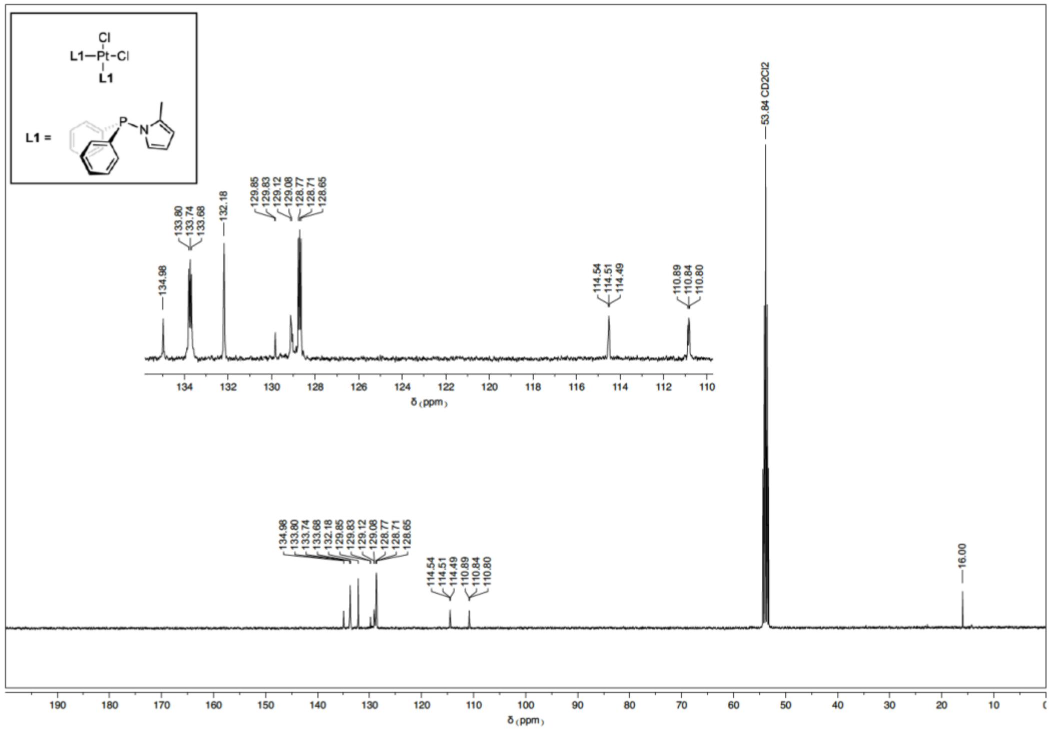

Figure S49. ^{13}C{^{1}H} NMR spectrum of *cis*-(**L1**)$_2$PtCl$_2$ in CD_2Cl_2 (101 MHz).

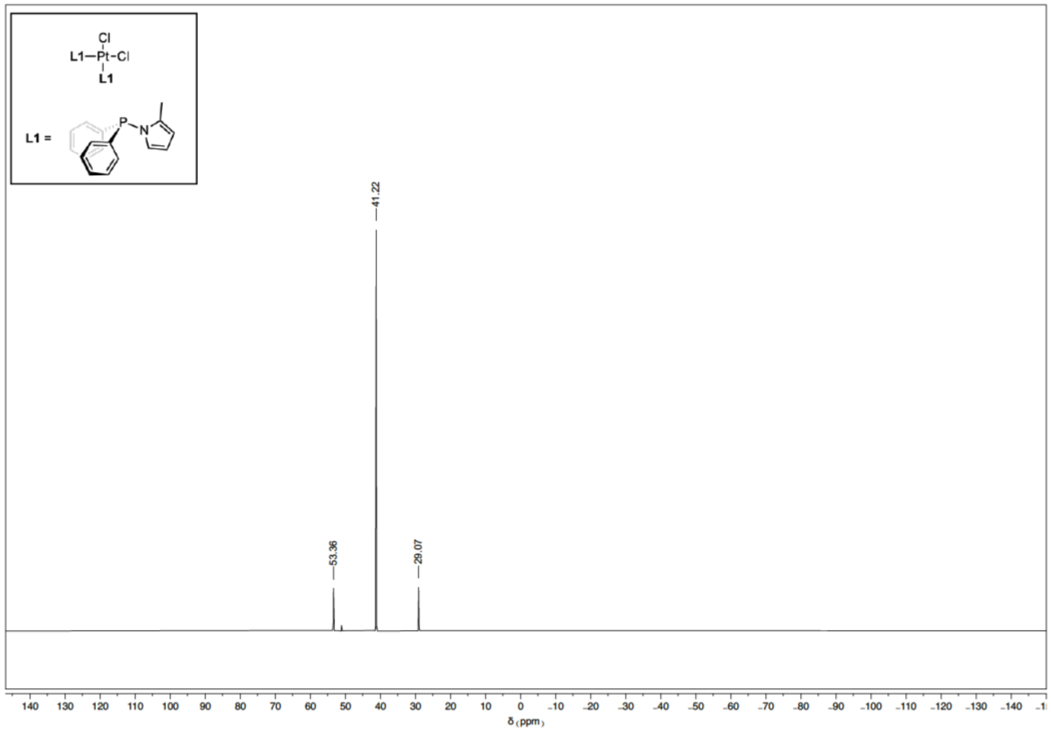

Figure S50. $^{31}P\{^{1}H\}$ NMR spectrum of *cis*-(**L1**)$_2$PtCl$_2$ in CD_2Cl_2 (162 MHz).

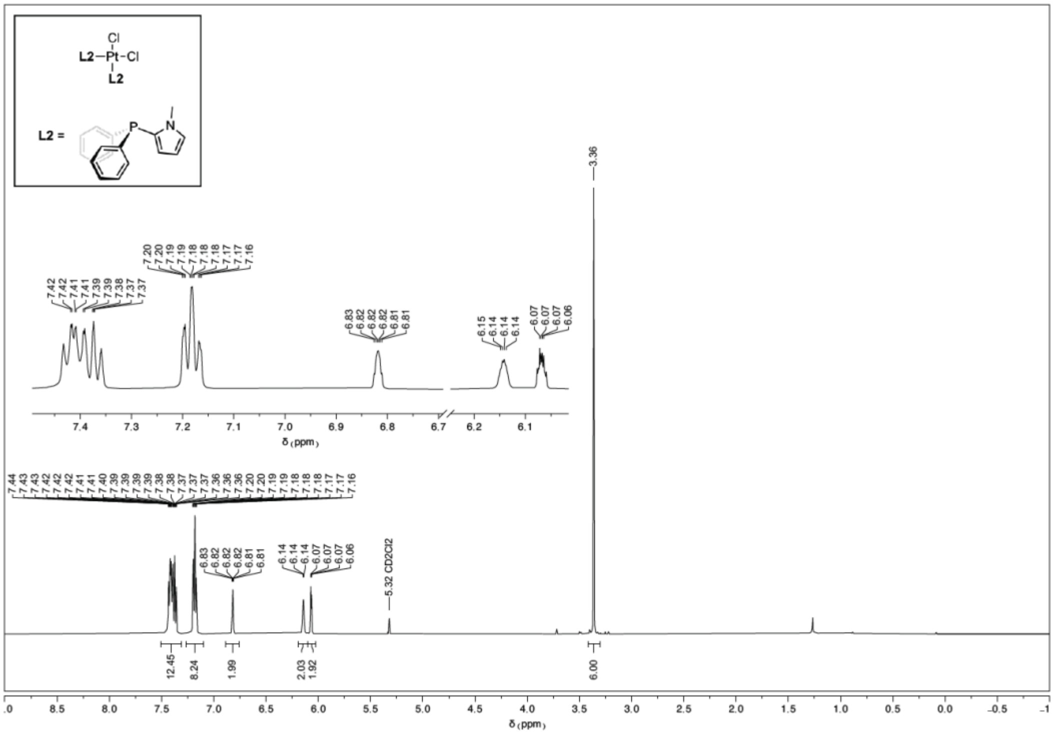

Figure S51. ^{1}H NMR spectrum of *cis*-(**L2**)$_2$PtCl$_2$ in CD$_2$Cl$_2$ (500 MHz).

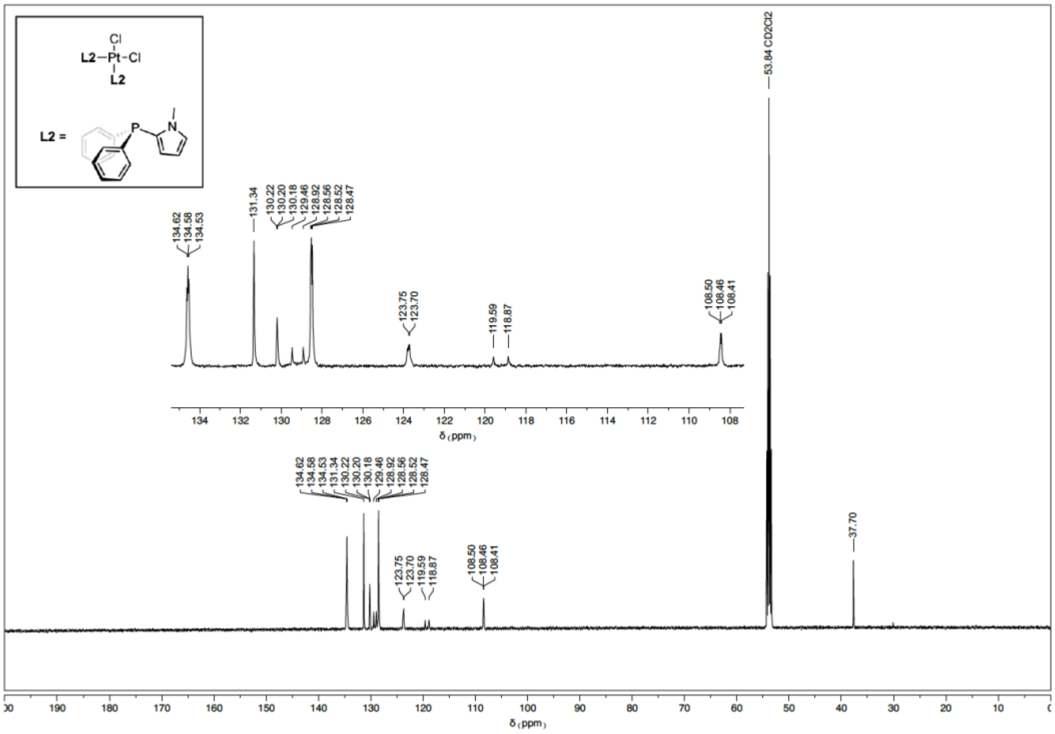

Figure S52. ^{13}C{^{1}H} NMR spectrum of *cis*-(**L2**)$_2$PtCl$_2$ in CD$_2$Cl$_2$ (126 MHz).

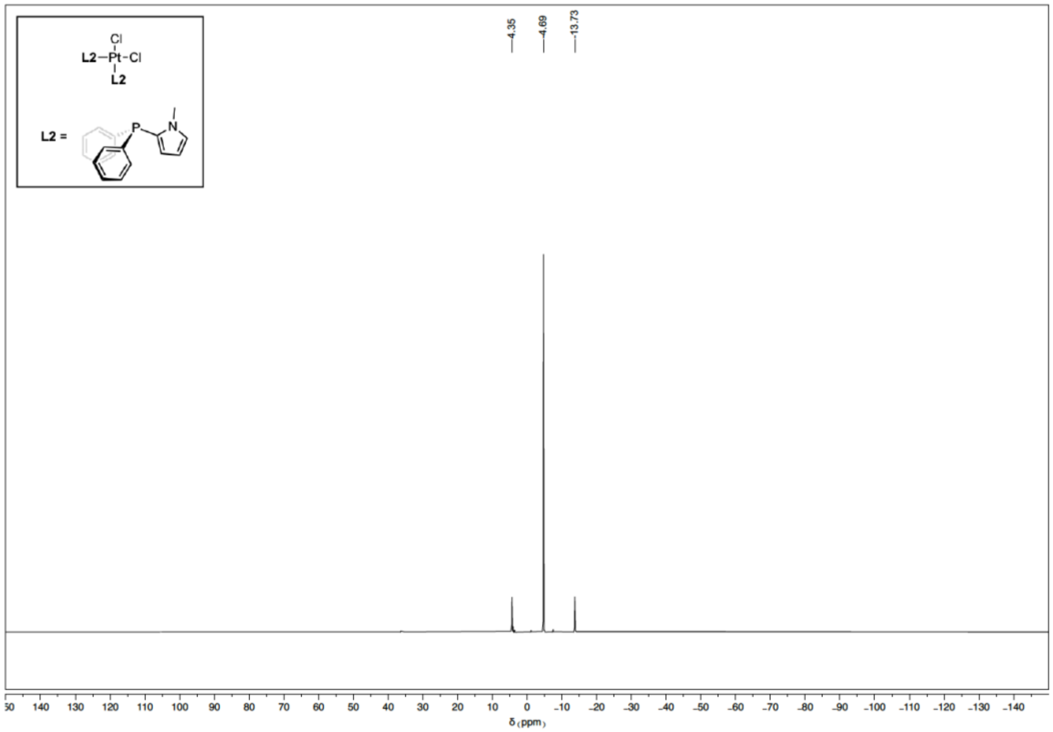

Figure S53. $^{31}P\{^1H\}$ NMR spectrum of *cis*-(**L2**)$_2$PtCl$_2$ in CD_2Cl_2 (202 MHz).

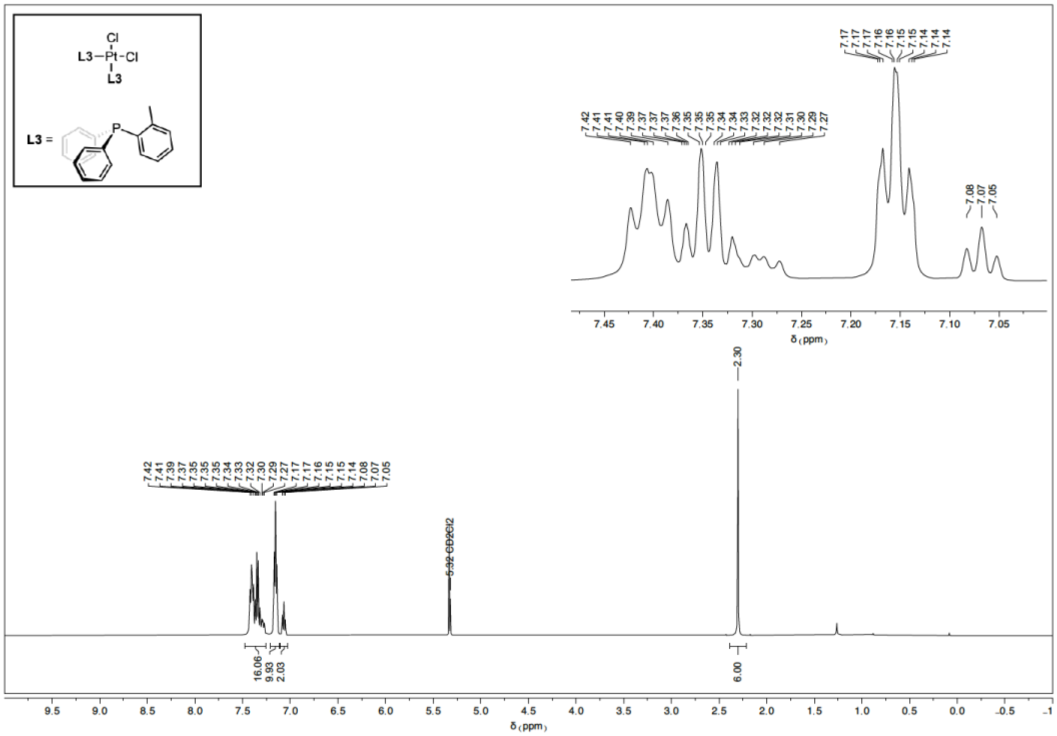

Figure S54. ^{1}H NMR spectrum of *cis*-(**L3**)$_2$PtCl$_2$ in CD_2Cl_2 (500 MHz).

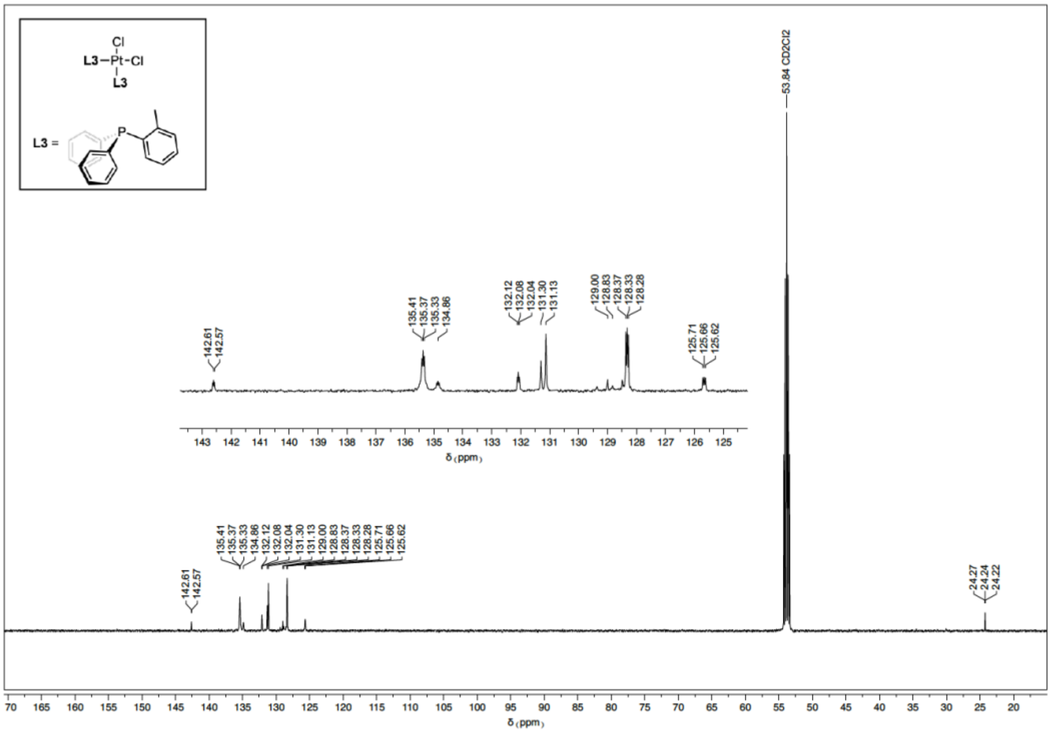

Figure S55. $^{13}C\{^{1}H\}$ NMR spectrum of *cis*-(**L3**)$_2$PtCl$_2$ in CD_2Cl_2 (126 MHz).

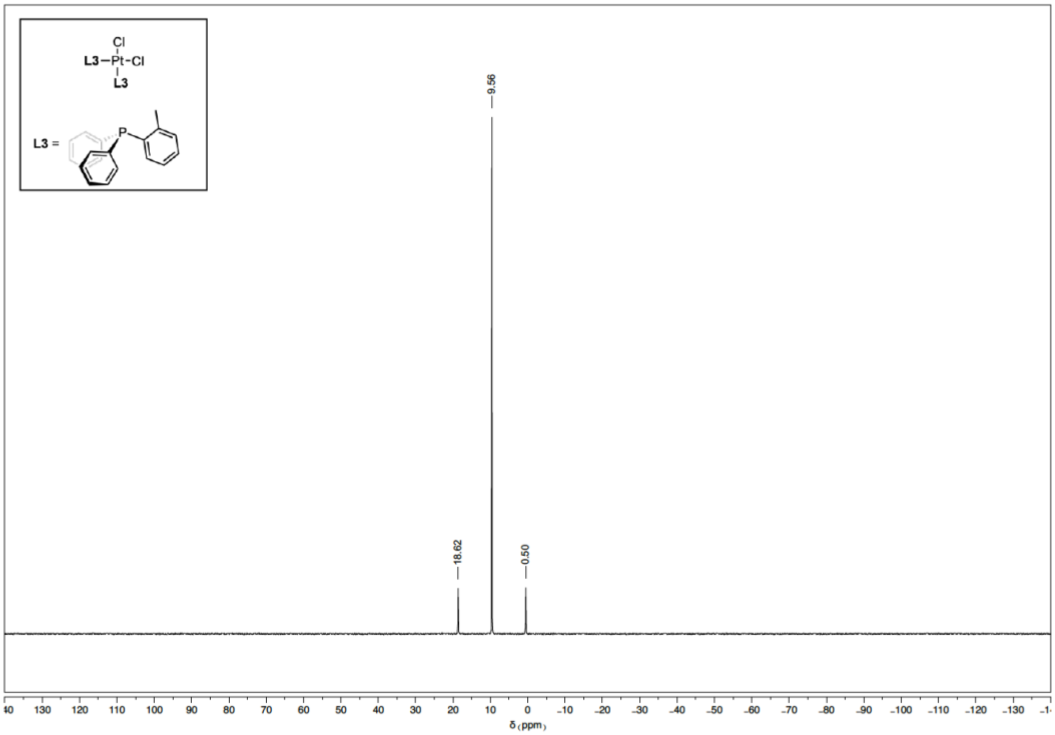

Figure S56. $^{31}P\{^{1}H\}$ NMR spectrum of *cis*-(**L3**)$_2$PtCl$_2$ in CD_2Cl_2 (202 MHz).

XX. NMR Spectra of Complexes (L)AuCl

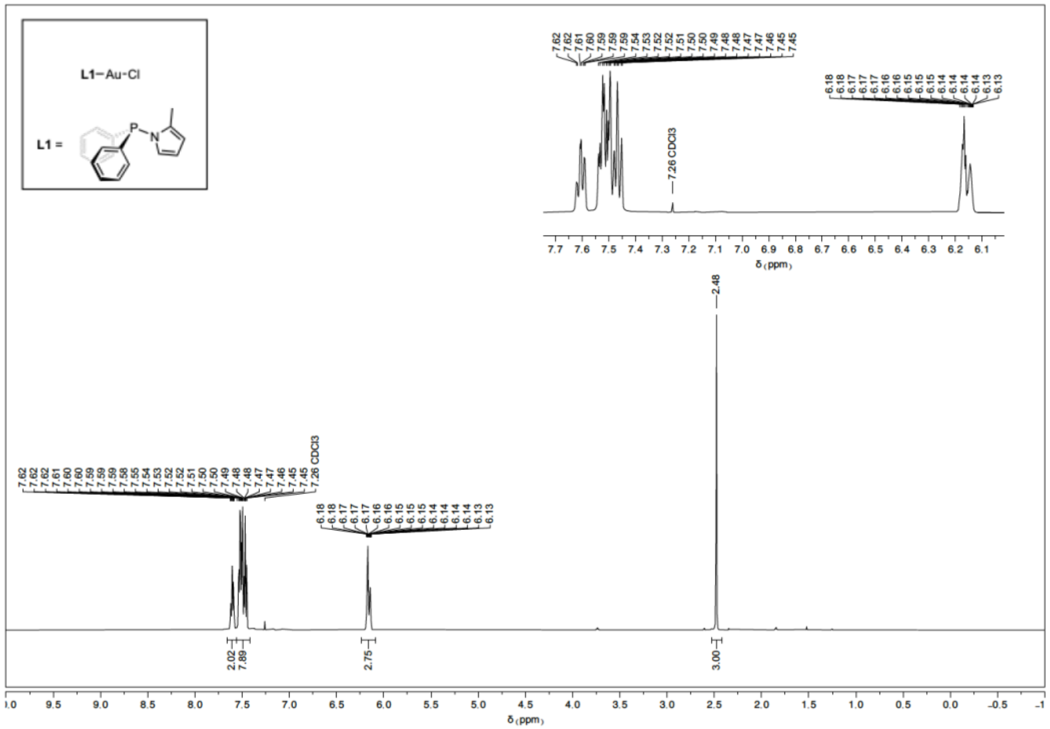

Figure S57. ^{1}H NMR spectrum of (**L1**)AuCl in $CDCl_3$ (500 MHz).

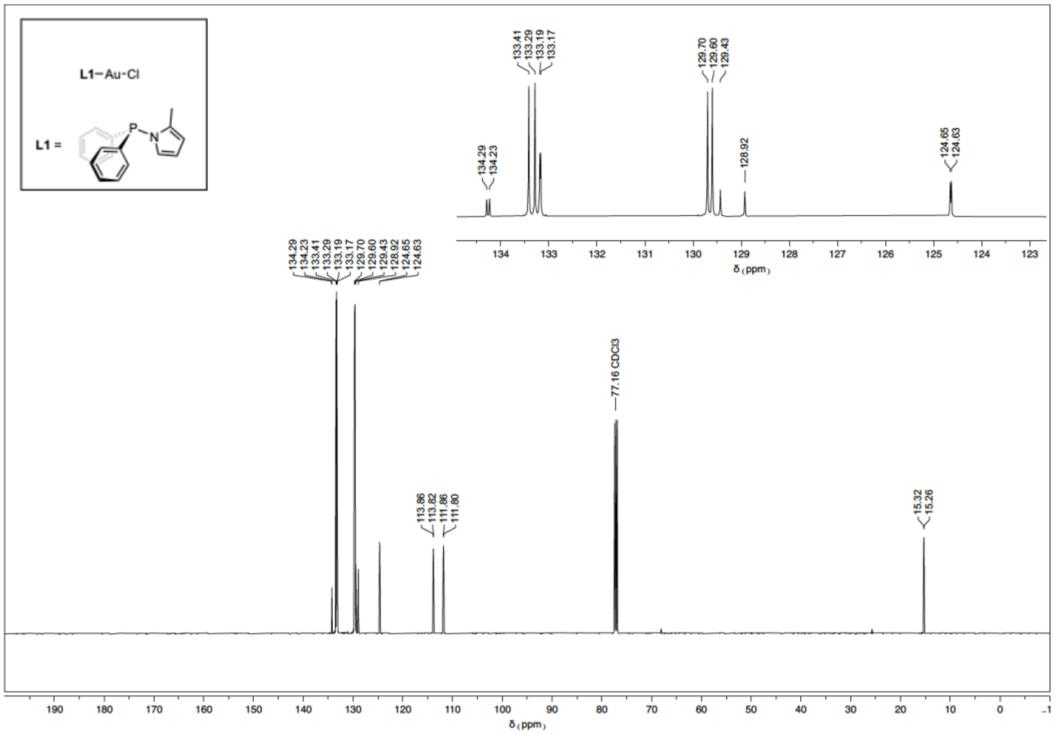

Figure S58. $^{13}C\{^{1}H\}$ NMR spectrum of (**L1**)AuCl in $CDCl_3$ (126 MHz).

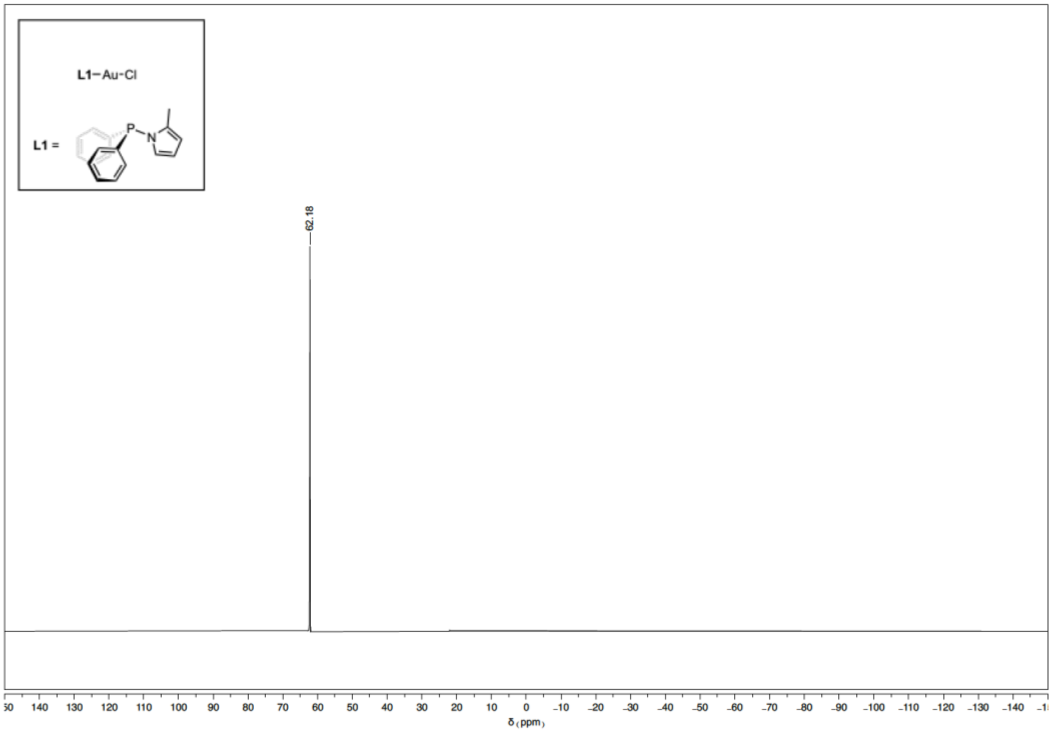

Figure S59. $^{31}P\{^{1}H\}$ NMR spectrum of (**L1**)AuCl in $CDCl_3$ (202 MHz).

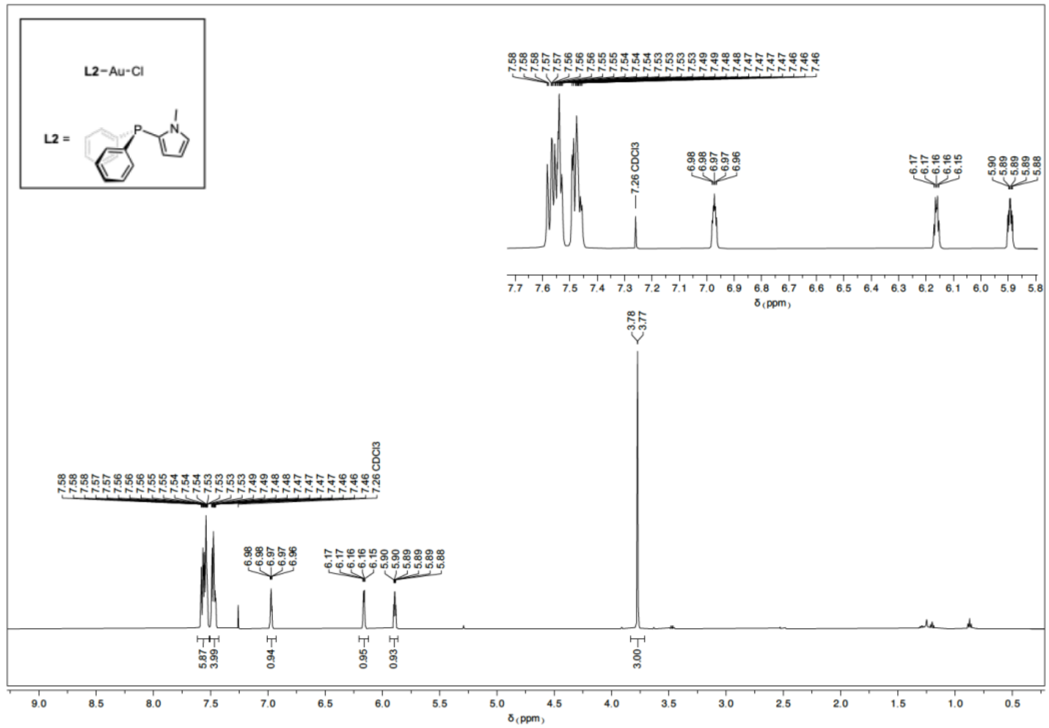

Figure S60. ^{1}H NMR spectrum of (**L2**)AuCl in $CDCl_3$ (500 MHz).

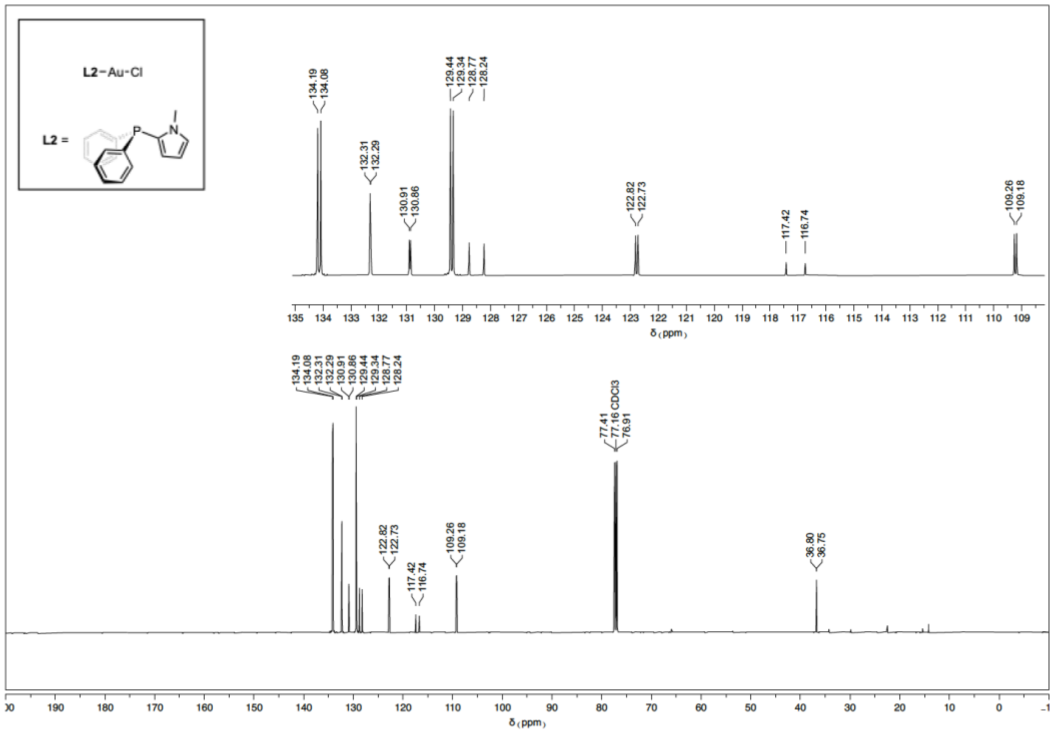

Figure S61. $^{13}C\{^{1}H\}$ NMR spectrum of (**L2**)AuCl in $CDCl_3$ (126 MHz).

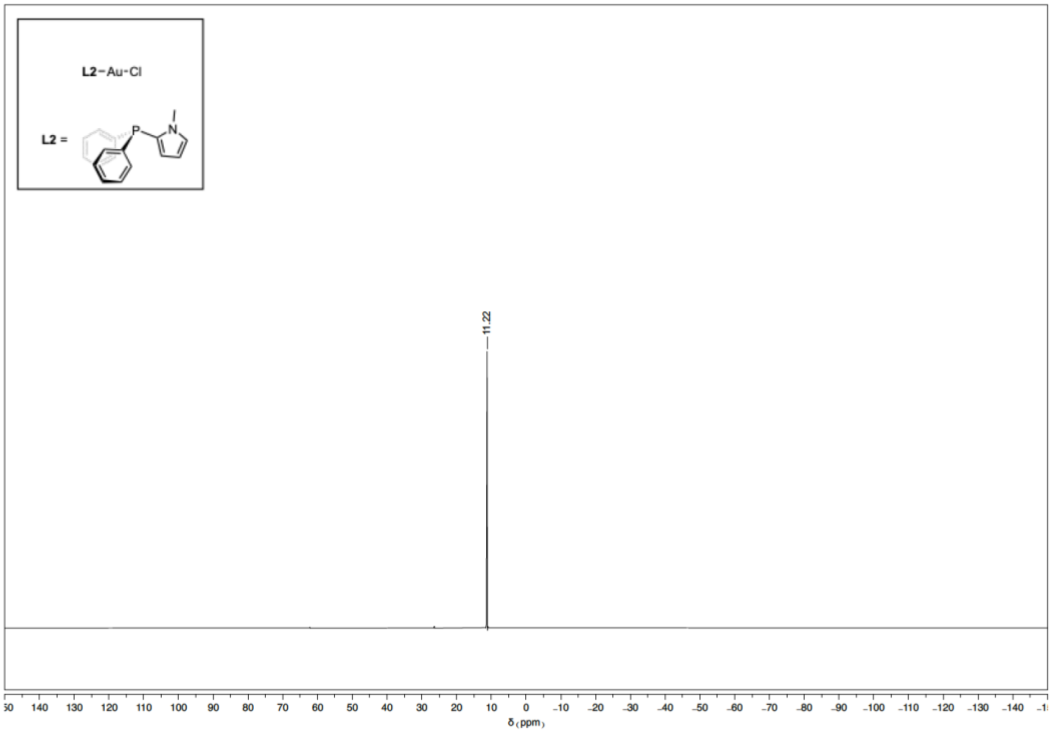

Figure S62. $^{31}P\{^{1}H\}$ NMR spectrum of (**L2**)AuCl in $CDCl_3$ (202 MHz).

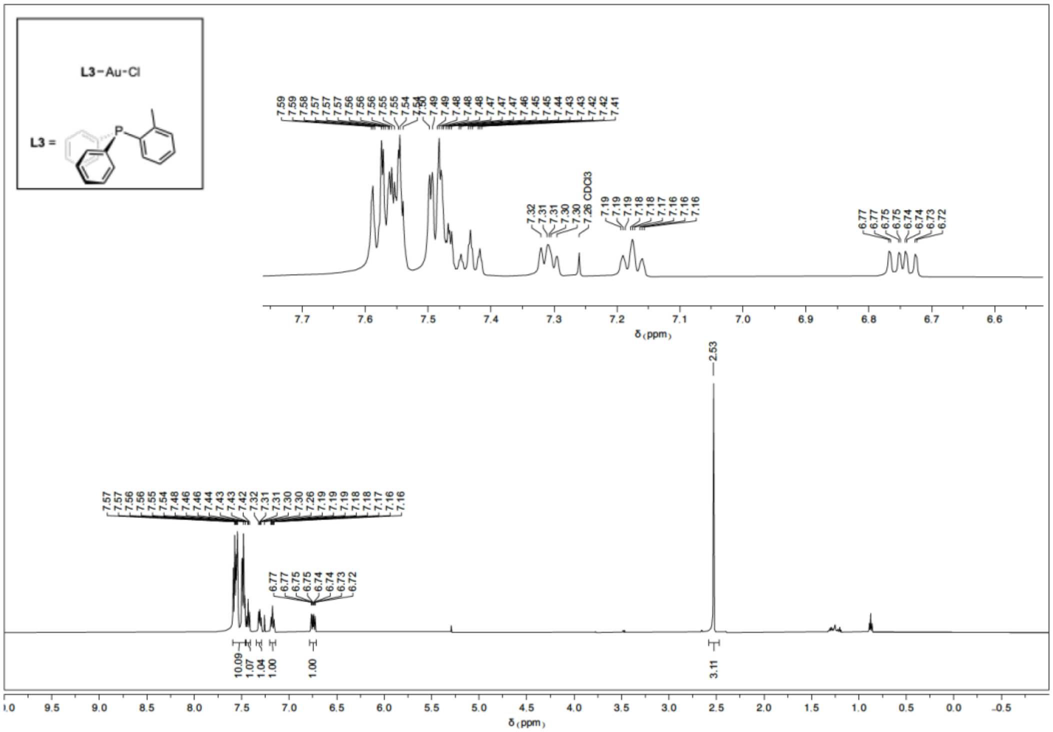

Figure S63. ^{1}H NMR spectrum of (**L3**)AuCl in $CDCl_3$ (500 MHz).

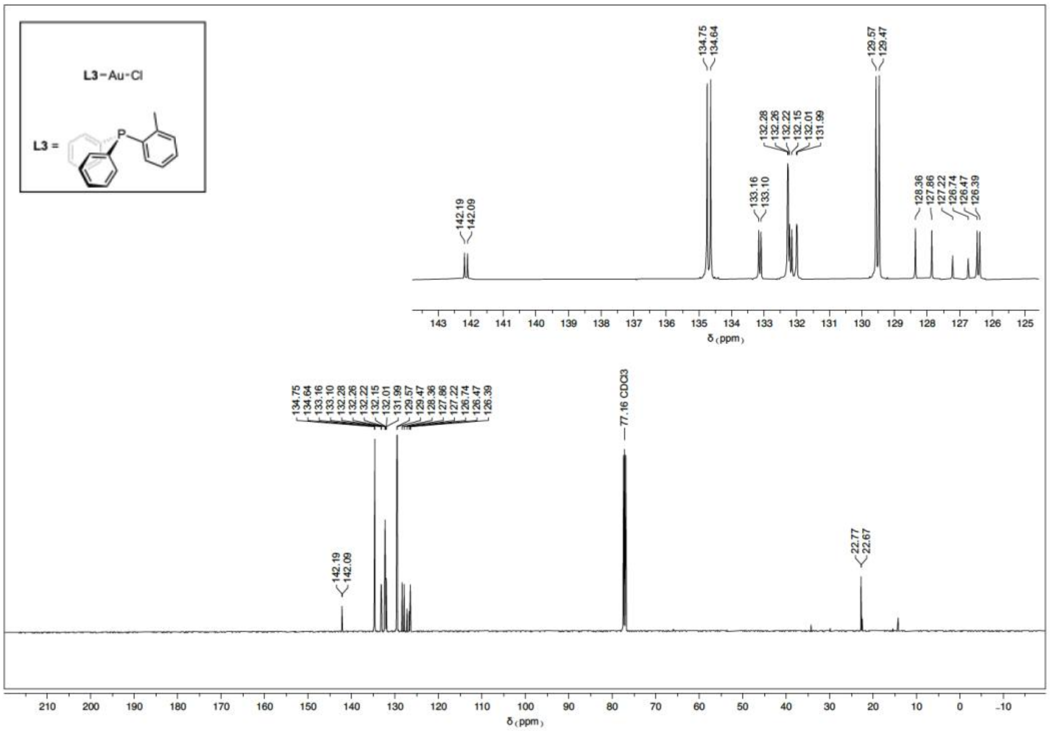

Figure S64. $^{13}C\{^{1}H\}$ NMR spectrum of (**L3**)AuCl in $CDCl_3$ (126 MHz).

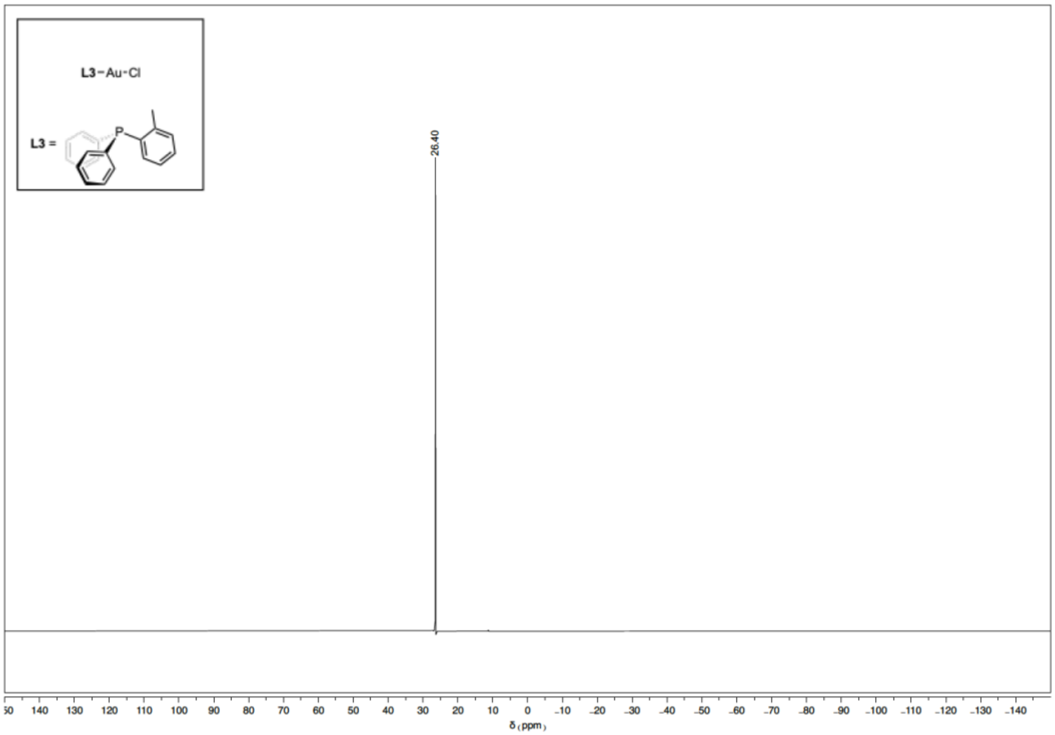

Figure S65. $^{31}P\{^1H\}$ NMR spectrum of (**L3**)AuCl in $CDCl_3$ (202 MHz).

XXI. X-Ray Crystallographic Data

Details of crystallographic refinement

General Methods. A suitable crystal of each sample was selected for analysis and mounted in a polyimide loop. Crystal samples were handled under immersion oil and quickly transferred to a cold nitrogen stream. All measurements were made on a Rigaku Oxford Diffraction Supernova Eos CCD with filtered Mo-Kα or Cu-Kα radiation at a temperature of 100 K. Using Olex2,[11] the structure was solved with the ShelXT structure solution program using Direct Methods and refined with the ShelXL refinement package[12] using Least Squares minimization.

(**L1**)$_2$Rh(CO)Cl
This structure was refined without additional restraints.

(**L2**)$_2$Rh(CO)Cl
This compound crystalized as a two-component non-merohedral twin. The components were integrated and refined against an hlkf 5 format reflection file. The refined ratio of the two components was 0.719(2) : 0.281(2). A disordered methylene chloride was modeled over two positions with similarity restraints placed on the bond distances and atomic thermal parameters.

(**L3**)$_2$Rh(CO)Cl
Positional disorder between the carbonyl and chloride was modeled over two positions with similarity restraints placed on the atomic thermal parameters and bond lengths.

(**L2**)$_2$PtCl$_2$
This structure was refined without additional restraints.

(**L3**)$_2$PtCl$_2$
This structure was refined without additional restraints.

(**L1**)AuCl
This structure was refined without additional restraints.

(**L2**)AuCl
Disorder was modeled over two positions with similarity restraints placed on atomic thermal parameters and atom distances.

(**L3**)AuCl
This structure was refined without additional restraints.

Table S3. Crystal data and structure refinement for *trans*-(**L1**)$_2$Rh(CO)Cl.

Empirical formula	$C_{35}H_{32}ClN_2OP_2Rh$	
Formula weight	696.92	
Temperature	100.00(10) K	
Wavelength	1.54184 Å	
Crystal system	Triclinic	
Space group	P-1	
Unit cell dimensions	a = 9.7770(2) Å	α = 117.030(2)°
	b = 13.5863(3) Å	β = 91.376(2)°
	c = 13.7992(3) Å	γ = 105.430(2)°
Volume	1551.34(6) $Å^3$	
Z	2	
Density (calculated)	1.492 Mg/m^3	
Absorption coefficient	6.461 mm^{-1}	
F(000)	712	
Crystal size	0.164 x 0.117 x 0.089 mm^3	
Theta range for data collection	3.648 to 71.755°.	
Index ranges	-12<=h<=11, -13<=k<=16, -16<=l<=16	
Reflections collected	23047	
Independent reflections	5985 [R(int) = 0.0318]	
Completeness to theta = 67.684°	99.9 %	
Absorption correction	Gaussian	
Max. and min. transmission	0.834 and 0.508	
Refinement method	Full-matrix least-squares on F^2	
Data / restraints / parameters	5985 / 0 / 381	
Goodness-of-fit on F^2	1.036	
Final R indices [I>2sigma(I)]	R1 = 0.0239, wR2 = 0.0607	
R indices (all data)	R1 = 0.0248, wR2 = 0.0614	
Absolute structure parameter	0.01(2)	
Extinction coefficient	n/a	
Largest diff. peak and hole	0.678 and -0.606 $e/Å^{-3}$	

Table S4. Crystal data and structure refinement for *trans*-(**L2**)$_2$Rh(CO)Cl.

Empirical formula	$C_{36}H_{34}Cl_3N_2OP_2Rh$	
Formula weight	781.85	
Temperature	100.00(10) K	
Wavelength	1.54184 Å	
Crystal system	Monoclinic	
Space group	P 1 21/n 1	
Unit cell dimensions	a = 13.01860(10) Å	α = 90°
	b = 13.12390(10) Å	β = 90.6310(10)°
	c = 20.1521(2) Å	γ = 90°
Volume	3442.87(5) $Å^3$	
Z	4	
Density (calculated)	1.508 Mg/m^3	
Absorption coefficient	7.283 mm^{-1}	
F(000)	1592	
Crystal size	0.268 x 0.212 x 0.143 mm^3	
Theta range for data collection	4.020 to 71.638°.	
Index ranges	-15<=h<=15, -16<=k<=14, -24<=l<=24	
Reflections collected	8268	
Independent reflections	8268 [R(int) = ?]	
Completeness to theta = 67.684°	99.9 %	
Absorption correction	Gaussian	
Max. and min. transmission	0.687 and 0.314	
Refinement method	Full-matrix least-squares on F^2	
Data / restraints / parameters	8268 / 69 / 437	
Goodness-of-fit on F^2	1.114	
Final R indices [I>2sigma(I)]	R1 = 0.0394, wR2 = 0.1369	
R indices (all data)	R1 = 0.0421, wR2 = 0.1391	
Absolute structure parameter	0.01(2)	
Extinction coefficient	n/a	
Largest diff. peak and hole	0.615 and -1.226 $e/Å^{-3}$	

Table S5. Crystal data and structure refinement for *trans*-(**L3**)$_2$Rh(CO)Cl.

Empirical formula	$C_{39.5}H_{35}Cl_2OP_2Rh$	
Formula weight	761.42	
Temperature	100.01(10) K	
Wavelength	1.54184 Å	
Crystal system	Monoclinic	
Space group	P 1 21/n 1	
Unit cell dimensions	a = 11.74930(10) Å	α = 90°
	b = 18.92450(10) Å	β = 107.9070(10)°
	c = 16.39690(10) Å	γ = 90°
Volume	3469.23(4) Å^3	
Z	4	
Density (calculated)	1.458 Mg/m^3	
Absorption coefficient	6.505 mm^{-1}	
F(000)	1556	
Crystal size	0.233 x 0.143 x 0.125 mm^3	
Theta range for data collection	3.672 to 71.777°.	
Index ranges	-14<=h<=14, -22<=k<=23, -19<=l<=19	
Reflections collected	33044	
Independent reflections	6735 [R(int) = 0.0321]	
Completeness to theta = 67.684°	100.0 %	
Absorption correction	Gaussian	
Max. and min. transmission	0.872 and 0.420	
Refinement method	Full-matrix least-squares on F^2	
Data / restraints / parameters	6735 / 13 / 454	
Goodness-of-fit on F^2	1.128	
Final R indices [I>2sigma(I)]	R1 = 0.0250, wR2 = 0.0588	
R indices (all data)	R1 = 0.0262, wR2 = 0.0594	
Absolute structure parameter	0.01(2)	
Extinction coefficient	n/a	
Largest diff. peak and hole	0.347 and -0.485 e/Å^{-3}	

Table S6. Crystal data and structure refinement for *cis*-(**L2**)$_2$PtCl$_2$.

Empirical formula	$C_{35}H_{34}Cl_4N_2P_2Pt$	
Formula weight	881.47	
Temperature	100.00(10) K	
Wavelength	0.71073 Å	
Crystal system	Monoclinic	
Space group	P 1 21/n 1	
Unit cell dimensions	a = 15.0677(4) Å	α = 90°
	b = 11.6161(2) Å	β = 109.257(3)°
	c = 20.5254(5) Å	γ = 90°
Volume	3391.51(15) $Å^3$	
Z	4	
Density (calculated)	1.726 Mg/m^3	
Absorption coefficient	4.576 mm^{-1}	
F(000)	1736	
Crystal size	0.32 x 0.18 x 0.14 mm^3	
Theta range for data collection	2.264 to 29.426°.	
Index ranges	-20<=h<=16, -15<=k<=14, -27<=l<=28	
Reflections collected	30660	
Independent reflections	8201 [R(int) = 0.0517]	
Completeness to theta = 25.242°	99.9 %	
Absorption correction	Gaussian	
Max. and min. transmission	1.000 and 0.389	
Refinement method	Full-matrix least-squares on F^2	
Data / restraints / parameters	8201 / 0 / 399	
Goodness-of-fit on F^2	1.072	
Final R indices [I>2sigma(I)]	R1 = 0.0414, wR2 = 0.0816	
R indices (all data)	R1 = 0.0515, wR2 = 0.0863	
Absolute structure parameter	0.01(2)	
Extinction coefficient	n/a	
Largest diff. peak and hole	1.718 and -1.660 $e/Å^{-3}$	

Table S7. Crystal data and structure refinement for *cis*-(**L3**)$_2$PtCl$_2$.

Empirical formula	$C_{40}H_{36}Cl_8P_2Pt$	
Formula weight	1057.32	
Temperature	100.01(10) K	
Wavelength	1.54184 Å	
Crystal system	Monoclinic	
Space group	P 1 21/n 1	
Unit cell dimensions	a = 20.0571(4) Å	α = 90°
	b = 11.7046(2) Å	β = 118.503(2)°
	c = 20.4281(4) Å	γ = 90°
Volume	4214.43(15) $Å^3$	
Z	4	
Density (calculated)	1.666 Mg/m^3	
Absorption coefficient	11.835 mm^{-1}	
F(000)	2080	
Crystal size	0.37 x 0.29 x 0.02 mm^3	
Theta range for data collection	2.540 to 72.055°.	
Index ranges	-20<=h<=24, -14<=k<=12, -25<=l<=24	
Reflections collected	30345	
Independent reflections	8110 [R(int) = 0.0491]	
Completeness to theta = 67.684°	99.9 %	
Absorption correction	Gaussian	
Max. and min. transmission	1.000 and 0.101	
Refinement method	Full-matrix least-squares on F^2	
Data / restraints / parameters	8110 / 0 / 462	
Goodness-of-fit on F^2	1.051	
Final R indices [I>2sigma(I)]	R1 = 0.0382, wR2 = 0.0904	
R indices (all data)	R1 = 0.0429, wR2 = 0.0935	
Absolute structure parameter	0.01(2)	
Extinction coefficient	n/a	
Largest diff. peak and hole	1.528 and -1.400 $e/Å^{-3}$	

Table S8. Crystal data and structure refinement for (**L1**)AuCl.

Empirical formula	$C_{17}H_{16}AuClNP$	
Formula weight	497.69	
Temperature	100.01(10) K	
Wavelength	1.54184 Å	
Crystal system	Monoclinic	
Space group	P 1 21/c 1	
Unit cell dimensions	a = 10.07620(10) Å	α = 90°
	b = 18.4440(3) Å	β = 92.6400(10)°
	c = 8.67590(10) Å	γ = 90°
Volume	1610.67(4) $Å^3$	
Z	4	
Density (calculated)	2.052 Mg/m^3	
Absorption coefficient	19.553 mm^{-1}	
F(000)	944	
Crystal size	0.16 x 0.09 x 0.04 mm^3	
Theta range for data collection	4.393 to 71.841°.	
Index ranges	-12<=h<=12, -22<=k<=22, -10<=l<=7	
Reflections collected	12261	
Independent reflections	3108 [R(int) = 0.0321]	
Completeness to theta = 67.684°	99.9 %	
Absorption correction	Gaussian	
Max. and min. transmission	0.585 and 0.201	
Refinement method	Full-matrix least-squares on F^2	
Data / restraints / parameters	3108 / 0 / 191	
Goodness-of-fit on F^2	1.084	
Final R indices [I>2sigma(I)]	R1 = 0.0241, wR2 = 0.0596	
R indices (all data)	R1 = 0.0259, wR2 = 0.0606	
Extinction coefficient	n/a	
Largest diff. peak and hole	0.955 and -1.294 $e/Å^{-3}$	

Table S9. Crystal data and structure refinement for (**L2**)AuCl.

Empirical formula	$C_{17}H_{16}AuClNP$	
Formula weight	497.69	
Temperature	100.00(10) K	
Wavelength	1.54184 Å	
Crystal system	Orthorhombic	
Space group	$P2_12_12_1$	
Unit cell dimensions	a = 9.9767(7) Å	α = 90°
	b = 12.1287(6) Å	β = 90°
	c = 13.3028(8) Å	γ = 90°
Volume	1609.70(17) $Å^3$	
Z	4	
Density (calculated)	2.054 Mg/m^3	
Absorption coefficient	19.564 mm^{-1}	
F(000)	944	
Crystal size	0.15 x 0.13 x 0.06 mm^3	
Theta range for data collection	4.934 to 71.644°.	
Index ranges	-12<=h<=11, -14<=k<=14, -16<=l<=14	
Reflections collected	8575	
Independent reflections	2985 [R(int) = 0.0374]	
Completeness to theta = 67.684°	99.9 %	
Absorption correction	Gaussian	
Max. and min. transmission	0.459 and 0.134	
Refinement method	Full-matrix least-squares on F^2	
Data / restraints / parameters	2985 / 470 / 310	
Goodness-of-fit on F^2	1.088	
Final R indices [I>2sigma(I)]	R1 = 0.0534, wR2 = 0.1159	
R indices (all data)	R1 = 0.0556, wR2 = 0.1175	
Absolute structure parameter	0.01(2)	
Extinction coefficient	n/a	
Largest diff. peak and hole	0.862 and -2.032 $e/Å^{-3}$	

Table S10. Crystal data and structure refinement for (**L3**)AuCl.

Empirical formula	$C_{19}H_{17}AuClP$	
Formula weight	508.71	
Temperature	100.01(10) K	
Wavelength	1.54184 Å	
Crystal system	Triclinic	
Space group	P-1	
Unit cell dimensions	a = 8.9634(3) Å	α = 116.941(4)°
	b = 10.3176(4) Å	β = 90.669(3)°
	c = 10.5187(3) Å	γ = 91.438(3)°
Volume	866.64(6) $Å^3$	
Z	2	
Density (calculated)	1.949 Mg/m^3	
Absorption coefficient	18.171 mm^{-1}	
F(000)	484	
Crystal size	0.27 x 0.07 x 0.05 mm^3	
Theta range for data collection	4.717 to 71.730°.	
Index ranges	-11<=h<=10, -9<=k<=12, -12<=l<=11	
Reflections collected	9483	
Independent reflections	3322 [R(int) = 0.0266]	
Completeness to theta = 67.684°	99.8 %	
Absorption correction	Gaussian	
Max. and min. transmission	0.627 and 0.098	
Refinement method	Full-matrix least-squares on F^2	
Data / restraints / parameters	3322 / 0 / 200	
Goodness-of-fit on F^2	1.090	
Final R indices [I>2sigma(I)]	R1 = 0.0302, wR2 = 0.0721	
R indices (all data)	R1 = 0.0305, wR2 = 0.0723	
Extinction coefficient	n/a	
Largest diff. peak and hole	2.173 and -1.977 e/$Å^{-3}$	

XXII. References

(1) Gagne, R. R.; Koval, C. A.; Lisensky, G. C. Ferrocene as an Internal Standard for Electrochemical Measurements. *Inorg. Chem.* **1980**, *19*, 2854–2855.

(2) Allen, D. W.; Hutley, B. G.; Mellor, M. T. J. The Chemistry of Heteroarylphosphorus Compounds. Part VI. Alkaline Hydrolysis of 1-Methylpyrrol-2-yl- and 1-Methylpyrrol-2-yl-Methyl-Phosphonium Salts. A Comparison with 2-Furyl, 2-Thienyl, Phenyl, and Related Heteroarylmethyl and Benzyl Derivatives. Relative Stabilities of Forming Carbanions. *J. Chem. Soc. Perkin Trans. 2* **1974**, 1690–1694.

(3) Chantson, J.; Görls, H.; Lotz, S. Heteroarylphosphorus Ligands in Platinum(II) Complexes. The Structure of Trans-[Pt(P{2-(N-Methylpyrrolyl)}iPr$_2$)$_2$Cl$_2$]. *Inorganica Chim. Acta* **2000**, *305*, 32–37.

(4) Allen, D. W.; Taylor, B. F. The Chemistry of Heteroarylphosphorus Compounds. Part 15. Phosphorus-31 Nuclear Magnetic Resonance Studies of the Donor Properties of Heteroarylphosphines towards Selenium and Platinum(II). *J. Chem. Soc. Dalton Trans.* **1982**, 51–54.

(5) Dean, P. A. W.; Polensek, L. A ^{31}P Nuclear Magnetic Resonance Spectroscopic Study of Some 2:1, 3:1, and 4:1 Complexes of Phosphine Sulfides and Selenides with Cadmium(II), Including Some Complexes with Mixed Ligands. *Can. J. Chem.* **1980**, *58*, 1627–1632.

(6) Bennett, M. A.; Longstaff, P. A. Reaction of Rhodium Halides with Tri-*o*-Tolylphosphine and Related Ligands. Complexes of Divalent Rhodium and Chelate Complexes Containing Rhodium-Carbon σ and μ Bonds. *J. Am. Chem. Soc.* **1969**, *91*, 6266–6280.

(7) Suomalainen, P.; Riihimäki, H.; Jääskeläinen, S.; Haukka, M.; Pursiainen, J. T.; Pakkanen, T. A. Structural and Catalytic Properties of Alkyl-Substituted Phosphanes. Effect of Ortho-Modification on Rhodium-Catalyzed 1-Hexene Hydroformylation. *Catal. Lett.* **2001**, *77*, 125–130.

(8) Nishina, N.; Yamamoto, Y. Gold-Catalyzed Intermolecular Hydroamination of Allenes: First Example of the Use of an Aliphatic Amine in Hydroamination. *Synlett* **2007**, 1767–1770.

(9) Falivene, L.; Cao, Z.; Petta, A.; Serra, L.; Poater, A.; Oliva, R.; Scarano, V.; Cavallo, L. Towards the Online Computer-Aided Design of Catalytic Pockets. *Nat. Chem.* **2019**, *11*, 872–879.

(10) Clavier, H.; Nolan, S. P. Percent Buried Volume for Phosphine and N-Heterocyclic Carbene Ligands: Steric Properties in Organometallic Chemistry. *Chem. Commun.* **2010**, *46*, 841–861.

(11) Dolomanov, O. V.; Bourhis, L. J.; Gildea, R. J.; Howard, J. A. K.; Puschmann, H. OLEX2: A Complete Structure Solution, Refinement and Analysis Program. *J. Appl. Crystallogr.* **2009**, *42*, 339–341.

(12) Sheldrick, G. A Short History of SHELX. *Acta Crystallogr. Sect. A* **2008**, *64*, 112–122.
